# A cross-sectional study of demographic variation in health problem-related limitations in life across 22 countries: a cross-national analysis

**DOI:** 10.1186/s44263-025-00190-6

**Published:** 2025-09-03

**Authors:** Jason Paltzer, Erik W. Carter, Elizabeth Kwon, Chukwuemeka N. Okafor, R Noah Padgett, Jessica Benfer, Tyler J. VanderWeele, Byron R. Johnson

**Affiliations:** 1 The Meros Center, Wisconsin Lutheran College, Milwaukee, WI USA; 2 Center for Developmental Disabilities, Baylor University, Waco, TX USA; 3 Department of Public Health, Baylor University, Waco, TX USA; 4 Division of Infectious Diseases, Long School of Medicine, Department of Medicine, University of Texas Health Science Center San Antonio, San Antonio, TX USA; 5 Department of Epidemiology, Human Flourishing Program, Harvard University, Harvard T.H. Chan School of Public Health, Boston, MA USA; 6 Department of Epidemiology and Biostatistics, Human Flourishing Program, Harvard University, Harvard T.H. Chan School of Public Health, Boston, MA USA; 7 Baylor University, Institute for Studies of Religion, Waco, TX USA; 8 Human Flourishing Program, Harvard University, Cambridge, MA USA

**Keywords:** Health problems, Health limitations, Global Flourishing Study, Global Health, Epidemiology

## Abstract

**Background:**

Health limitations, defined as problems in life that limit an individual from activities of daily living, have been associated with human flourishing, globally. However, how health problems leading to functional limitations vary across multiple demographic characteristics and how these vary across countries requires additional exploration.

**Methods:**

The Global Flourishing Study is a 5-year longitudinal study of human flourishing among 202,898 individuals across 22 countries. The purpose of this analysis was to assess the regional and demographic variation of health limitations in life associated with health problems in wave 1 of the Global Flourishing Study. The study explored the distributions and descriptive statistics of key demographic features (age, gender, marital status, employment, religious service attendance, education, immigration status) across and within countries. The following hypotheses were tested: (1) the distributions and descriptive statistics of key demographic factors will reveal diverse patterns across the international sample, (2) the proportions of health limitations will vary meaningfully across different countries, and (3) health limitations will exhibit variations across different demographic categories such as age, gender, marital status, employment, religious service attendance, education, and immigration status. These differences across demographic categories will vary by country.

**Results:**

The proportion of health limitations was greater among older age individuals and those with fewer years of education. Being widowed and retired showed higher proportions of having a health limitation compared to those married and working, respectively. The Philippines (0.34), United Kingdom (0.31), and Germany (0.30) had the highest proportions of health problems, while Israel (0.14), Turkey (0.14), and Poland (0.13) had the lowest.

**Conclusions:**

Understanding key demographic associations of such health problems and daily functioning could help inform policy, programs, and practices aimed at addressing the well-being of individuals who experience health limitations.

**Supplementary Information:**

The online version contains supplementary material available at 10.1186/s44263-025-00190-6.

## Background

Health problems resulting in limitations that hinder an individual from conducting activities of daily living are critical to understanding human flourishing. Such problems can lead to functional limitations in seeing, hearing, mobility, communication, cognition, and self-care [1]. There are a variety of health problems leading to limitations, including neurological disorders. The Global Burden of Disease project recently found nervous disorders were the leading determinants of Disability Adjusted Life Years in 2021 [2]. Given the scope of such health problems and the significant impact of health limitations on daily functioning, it is critical to study cross-cultural risk factors associated with such problems and how they influence human flourishing.

Health problems are linked to human flourishing, defined as living the good life and achieving well-being in all areas [3]. Mental, physical, and social health limitations influence other flourishing domains, including “happiness and satisfaction, meaning and purpose, character and virtue, close social relationships” and financial and material stability [3]. In addition, the attitudes and stigma associated with some health limitations can further marginalize people with underlying health problems [4, 5]. Understanding health limitations in relation to demographic risk factors is an essential step in promoting global flourishing.

Health problems associated with limitations can include neurologic disorders, musculoskeletal conditions, chronic conditions, and issues related to physical or cognitive abilities [6]. The limitations associated with such health problems can vary by education, income, cultural perceptions, social policies, and family support mechanisms available [7]. Health limitations have been associated with an increased risk of stroke and mediated overall survival time [8, 9].

Several factors are known to be highly associated with increased risk for health limitations. Education is associated with health limitations, especially among older adults [10]. Lower educational status is associated with higher levels of health problems and related functional limitations. The mechanism linking education and health limitations, however, is complex and might vary across countries [11]. Age is also associated with increasing limitations in daily functioning. Over 40% of the adult US population reported having difficulty with routine activities of daily living, with increasing levels of functional limitations reported by individuals 65 years and older [1]. Of note is that the rates of limitations were more prevalent among middle-aged, female, Hispanic, and non-Hispanic Black adults [12].

Evidence suggests the need for understanding common risk factors for health problems and related limitations in the context of different socioeconomic, cultural, and religious backgrounds. Religiosity and spirituality show protective effects by reducing the impact of health problems on functional limitations leading to reduced mortality and morbidity [13]. In a repeated mixed measures study, religious service attendance was associated with fewer limitations in activities of daily living and mobility 3–4 years later [14]. Service attendance and religious coping support the influence of hope on well-being [15]. Hope can provide a buffer in times of stress often encountered when dealing with a health limitation, such as was observed during the COVID-19 pandemic [15]. The interaction between hope and spirituality influences an individual’s level of flourishing in the context of health limitations. However, when a health limitation minimizes social participation, hopelessness might result. Hopelessness was found to be a mediator between social disconnection and flourishing [16]. It is important to recognize the influence of such participation leading to flourishing and the impact of health limitations on participation.

Prior research indicates there are demographic variations across countries regarding functional health limitations. For example, studies show commonalities across countries by income, multiple comorbidities, and depression associated with disabilities in activities of daily living [17]. Studies also reveal ethnicity, access to social capital, marital status, and years of education lead to differences in functional disability across countries [17–19]. Such studies highlight the need to further explore the direction and strength of the relationship between health limitations and flourishing in the context of different cultures, religious beliefs, and social structures.

The Global Flourishing Study (GFS) involving 202,898 individuals from 22 countries provides a unique opportunity to understand regional and cultural variations in the association between specific demographic factors and health-related limitations. The overall (GFS) project takes a global and longitudinal view of flourishing. Health limitation was included as one indicator of the Human Flourishing Physical and Mental Health domain. The decision to use a single-item question to evaluate health limitations was based on the need to accommodate the multiple domains of flourishing. Our descriptive analysis explores key demographic features (age, gender, marital status, employment, religious service attendance, education, immigration status) across an international sample and their association with health problems linked to limitations. The hypotheses tested in this study are:

- Hypothesis #1: The distributions and descriptive statistics of key demographic features (age, gender, marital status, employment, religious service attendance, education, immigration status) will reveal diverse patterns across the 22 countries.
- Hypothesis #2: The mean levels of health problems associated with limitations will vary meaningfully across different countries.
- Hypothesis #3: Health problems associated with limitations will exhibit variations across different demographic categories such as age, gender, marital status, employment, religious service attendance, education, and immigration status. We hypothesize that these differences across demographic categories will vary by country.

## Methods

The description of the methods below has been adapted from VanderWeele et al. [20]. Further methodological detail is available in more detailed articles describing the overall design of the GFS [21–28].

### Data

The GFS is a study of 202,898 participants from 22 geographically and culturally diverse countries, with national sampling within each country, concerning the distribution of determinants of well-being. Wave 1 of the data included the following countries and territories: Argentina, Australia, Brazil, Egypt, Germany, Hong Kong (Special Administrative Region of China, with mainland China also included from 2024 onwards), India, Indonesia, Israel, Japan, Kenya, Mexico, Nigeria, the Philippines, Poland, South Africa, Spain, Sweden, Tanzania, Turkey, United Kingdom, and the USA. The countries were selected to (a) maximize coverage of the world’s population; (b) ensure geographic, cultural, and religious diversity; and (c) prioritize feasibility and existing data collection infrastructure. Data collection was carried out by Gallup Inc. Data for Wave 1 were collected principally during 2023, with some countries beginning data collection in April 2022 and exact dates varying by country [26]. Four additional waves of panel data on the participants will be collected annually from 2024 to 2027. The precise sampling design and weighting procedures varied by country, and further details are available elsewhere [26]. Survey items included aspects of well-being such as happiness, health, meaning, character, relationships, and financial stability [3], along with other demographic, social, economic, political, religious, personality, childhood, community, health, and well-being variables. The data are publicly available through the Center for Open Science (COS) (https://www.cos.io/gfs) [28]. During the translation process, Gallup adhered to the TRAPD model (translation, review, adjudication, pretesting, and documentation) for cross-cultural survey research (ccsg.isr.umich.edu/chapters/translation/overview) [29].

### Measures

#### Outcome variable(s)

The presence of any health problem associated with limitations was measured as a binary variable [Yes/No] with the question: Do you have any health problems that prevent you from doing any of the things people your age normally can do?

#### Demographics variables

Continuous age was classified as 18–24, 25–29, 30–39, 40–49, 50–59, 60–69, 70–79, and 80 or older. Gender was assessed as male, female, or other. Marital status was assessed as single/never married, married, separated, divorced, widowed, and domestic partner. Employment was assessed as employed, self-employed, retired, student, homemaker, unemployed and searching, and other. Education was assessed as up to 8 years, 9–15 years, and 16 + years. Service attendance was assessed as more than once/week, once/week, one-to-three times/month, a few times/year, or never. Immigration status was dichotomously assessed with: “Were you born in this country, or not?” Religious tradition/affiliation with categories of Christianity, Islam, Hinduism, Buddhism, Judaism, Sikhism, Baha’i, Jainism, Shinto, Taoism, Confucianism, Primal/Animist/Folk religion, Spiritism, African-Derived, some other religion, or no religion/atheist/agnostic; precise response categories varied by country [30]. Racial/ethnic identity was assessed in some, but not all, countries, with response categories varying by country. For additional details on the assessments, see the COS GFS questionnaire development report (https://osf.io/y3t6m/) or elsewhere [21, 22].

### Analysis

Descriptive statistics for the full sample, weighted within each country, were estimated for each of the demographic variables. National means/proportions for the presence of health problems associated with limitations were estimated separately for each country and ordered from highest to lowest, along with 95% confidence intervals. Variation in proportions in health problems across demographic categories was estimated, with all analyses initially conducted by country (Additional file 1). Primary results consisted of a random effects meta-analysis of country-specific means of health problems in each specific demographic category [31, 32] along with 95% confidence intervals, standard errors, upper and upper limits of a 95% prediction interval across countries, heterogeneity (τ), and *I*^2^ for evidence concerning variation within a particular demographic variable across countries [33]. Forest plots of estimates are available in Additional file 1. The random effects meta-analysis approach was selected rather than multilevel modeling because of the variance across countries violating the assumption of invariance required for multilevel modeling. The GFS is, in effect, 22 distinct yet coordinated studies. The random effects meta-analysis allowed us to pool the results across countries, maintaining the heterogeneity at the country level. The country-specific tables in Additional file 1 show the computed within-group weighted proportions as point estimates for each demographic group category. The forest plots display the meta-analyzed effect estimate as well as the country-specific estimates, ordered by magnitude, for each demographic sub-group category. All meta-analyses were conducted in R [34] using the metafor package [35]. Within each country, a global test of variation of outcome across levels of each particular demographic variable was conducted, and a pooled *p*-value [36] across countries was reported concerning evidence for variation within any country. Bonferroni corrected *p*-value thresholds are provided based on the number of demographic variables as a way to account and adjust for multiple testing [37, 38]. Religious affiliation/tradition and race/ethnicity were used, when available, as control variables within countries, but were not included in the meta-analyses since the availability of these response categories varied by country. All reported subgroup means are unadjusted to provide a descriptive view of these data. As a supplementary analysis, population-weighted meta-analyses were also conducted. Model choice and additional analytical methods are further discussed in Padgett et al. (2025) [23]. All analyses were pre-registered with COS prior to data access (https://osf.io/uk9ay) [25]; all code to reproduce analyses are openly available in an online repository (https://osf.io/vbype) [24].

### Missing data

Missing data on all variables were imputed using multivariate imputation by chained equations, and five imputed datasets were used [39–42]. To account for variation in the assessment of certain variables across countries (e.g., religious affiliation/tradition and race/ethnicity), the imputation process was conducted separately in each country. This within-country imputation approach ensured that the imputation models accurately reflected country-specific contexts and assessment methods. Sampling weights were included in the imputation model to account for specific-variable missingness that may have been related to the probability of inclusion in the study.

### Accounting for complex sampling design

The GFS used different sampling schemes across countries based on the availability of existing panels and recruitment needs [26]. All analyses accounted for the complex survey design components by including weights, primary sampling units, and strata. Additional methodological detail, including accounting for the complex sampling design, is provided elsewhere [26, 27].

The STROBE guidelines were followed in preparing the manuscript (Additional file 2) [43].

## Results

Table 1 shows the demographic information of this sample across all countries. The sample was predominantly middle-aged (30–59 years) (53%), female (51%), and married (53%) individuals. Most individuals (78%) had 9 years or more of education, 57% were employed or self-employed, and 32% attended religious services once a week or more. Respondents were predominantly (94%) born in their country of residence.

**Table 1:** Descriptive statistics of the observed sample

Characteristic	*N *= 202,898^a^
Age group	
18–24	27,007 (13%)
25–29	20,700 (10%)
30–39	40,256 (20%)
40–49	34,464 (17%)
50–59	31,793 (16%)
60–69	27,763 (14%)
70–79	16,776 (8.3%)
80 or older	4119 (2.0%)
(Missing)	20 (< 0.1%)
Gender	
Male	98,411 (49%)
Female	103,488 (51%)
Other	602 (0.3%)
(Missing)	397 (0.2%)
Marital status	
Married	107,354 (53%)
Separated	5,195 (2.6%)
Divorced	11,654 (5.7%)
Widowed	9823 (4.8%)
Never	52,115 (26%)
Domestic partner	14,931 (7.4%)
(Missing)	1826 (0.9%)
Employment	
Employed for an employer	78,815 (39%)
Self-employed	36,362 (18%)
Retired	29,303 (14%)
Student	10,726 (5.3%)
Homemaker	21,677 (11%)
Unemployed and looking for a job	16,790 (8.3%)
None of these/other	8431 (4.2%)
(Missing)	793 (0.4%)
Religious service attendance	
At least 1/week	26,537 (13%)
1/week	39,157 (19%)
1–3/month	19,749 (9.7%)
A few times a year	41,436 (20%)
Never	75,297 (37%)
(Missing)	722 (0.4%)
Education	
Up to 8 years	45,078 (22%)
9–15 years	115,097 (57%)
16+ years	42,578 (21%)
(Missing)	146 (< 0.1%)
Immigration	
Born in this country	190,998 (94%)
Born in another country	9791 (4.8%)
(Missing)	2110 (1.0%)
Country	
Argentina	6724 (3.3%)
Australia	3844 (1.9%)
Brazil	13,204 (6.5%)
Egypt	4729 (2.3%)
Germany	9506 (4.7%)
Hong Kong	3012 (1.5%)
India	12,765 (6.3%)
Indonesia	6992 (3.4%)
Israel	3669 (1.8%)
Japan	20,543 (10%)
Kenya	11,389 (5.6%)
Mexico	5776 (2.8%)
Nigeria	6827 (3.4%)
Philippines	5292 (2.6%)
Poland	10,389 (5.1%)
South Africa	2651 (1.3%)
Spain	6290 (3.1%)
Sweden	15,068 (7.4%)
Tanzania	9075 (4.5%)
Turkey	1473 (0.7%)
United Kingdom	5368 (2.6%)
USA	38,312 (19%)

^a^*n *(%)

Table 2 shows the variation in the proportion of health problems by country, with the lowest proportion being 0.13 and the highest being 0.34. The top 5 countries with the highest proportions of health problems were the Philippines (0.34, 95% CI 0.33, 0.36), United Kingdom (0.31, 95% CI 0.29, 0.33), Germany (0.30, 95% CI 0.28, 031), India (0.28, 95% CI 0.27, 0.30), and Australia (0.28, 95% CI 0.26, 0.29). The five countries with the lowest proportions of reported health problems were Poland (0.13, 95% CI 0.12, 0.15), Turkey (0.14, 95% CI 0.11, 0.16), Israel (0.14, 95% 0.12, 016), Nigeria (0.14, 95% CI 0.12, 0.16), and Kenya (0.15, 95% CI 0.14, 0.16).

**Table 2:** Ordered proportions of health problems (yes vs. no)

Country	Proportion	95% CI	SE
Philippines	0.34	(0.33, 0.36)	0.008
United Kingdom	0.31	(0.29, 0.33)	0.009
Germany	0.30	(0.28, 0.31)	0.006
India	0.28	(0.27, 0.30)	0.006
Australia	0.28	(0.26, 0.29)	0.009
Sweden	0.26	(0.25, 0.27)	0.004
Egypt	0.25	(0.24, 0.27)	0.009
USA	0.24	(0.23, 0.26)	0.005
South Africa	0.22	(0.20, 0.24)	0.013
Hong Kong	0.21	(0.19, 0.23)	0.011
Spain	0.20	(0.18, 0.21)	0.007
Brazil	0.19	(0.18, 0.20)	0.005
Argentina	0.19	(0.17, 0.20)	0.007
Japan	0.18	(0.17, 0.18)	0.003
Mexico	0.17	(0.16, 0.19)	0.006
Tanzania	0.16	(0.15, 0.17)	0.006
Indonesia	0.15	(0.14, 0.16)	0.007
Kenya	0.15	(0.14, 0.16)	0.005
Nigeria	0.14	(0.12, 0.16)	0.008
Israel	0.14	(0.12, 0.16)	0.012
Turkey	0.14	(0.11, 0.16)	0.012
Poland	0.13	(0.12, 0.15)	0.008

*N* = 202,898, *95% CI* = 95% confidence interval of the proportion, *SE* = standard error; the combination of small SE and rounding can lead to non-symmetric CI for some countries

Table 3 presents the random effects meta-analysis of health problem proportions by each demographic variable. The overall proportion of health problems across all countries is 0.21 with 95% CI (0.18, 0.24) with an estimated heterogeneity (tau) across countries of 0.06 standard deviations. Health problems associated with limitations increase with age, with higher proportions identified among females and lower levels of education. Being married, single, or having a domestic partner is associated with lower proportions of health problems compared to being widowed, divorced, or separated. Individuals in retirement or working as homemakers tend to have higher reported health problems. Religious service attendance shows slightly higher proportions of health problems among those attending more than once a week and never attending compared to moderate attenders.

**Table 3:** Random effects meta-analysis of health problems (yes vs. no) proportions by demographic category

				Prediction interval				
Variable category	Proportion	95% CI of proportion	SE analog^1^	LL	UL	Heterogeneity (τ)	${I}^{2}$	Global *p*-value
Age group								< .001**
18–24	0.13	(0.10,0.16)	0.01	0.03	0.25	0.06	89.9	
25–29	0.14	(0.11,0.17)	0.01	0.05	0.24	0.06	89.3	
30–39	0.15	(0.12,0.18)	0.01	0.05	0.29	0.06	88.2	
40–49	0.20	(0.17,0.23)	0.02	0.07	0.36	0.07	86.7	
50–59	0.25	(0.22,0.29)	0.02	0.14	0.41	0.07	85.7	
60–69	0.32	(0.29,0.36)	0.02	0.20	0.51	0.09	86.3	
70–79	0.39	(0.35,0.43)	0.02	0.22	0.60	0.08	84.3	
80 or older	0.39	(0.18,0.66)	0.12	0.00	1.00	0.62	99.6	
Gender								< .001**
Male	0.19	(0.17,0.22)	0.01	0.13	0.34	0.05	80.5	
Female	0.22	(0.19,0.25)	0.01	0.15	0.32	0.06	82.3	
Other	0.06	(0.01,0.29)	0.07	0.00	0.64	0.23	99.8	
Marital status								< .001**
Married	0.20	(0.18,0.23)	0.01	0.12	0.36	0.06	83.2	
Separated	0.29	(0.23,0.35)	0.03	0.07	0.81	0.14	94.7	
Divorced	0.28	(0.24,0.32)	0.02	0.12	0.47	0.10	90.4	
Widowed	0.38	(0.35,0.42)	0.02	0.22	0.52	0.08	83.8	
Domestic partner	0.16	(0.09,0.27)	0.04	0.00	0.38	0.20	98.7	
Single, never married	0.16	(0.13,0.19)	0.02	0.07	0.28	0.07	89.8	
Employmentstatus								< .001**
Employed for an employer	0.15	(0.12,0.17)	0.01	0.07	0.31	0.06	87.6	
Self-employed	0.18	(0.15,0.21)	0.01	0.07	0.35	0.07	87.0	
Retired	0.37	(0.33,0.42)	0.02	0.18	0.65	0.11	90.0	
Student	0.11	(0.08,0.15)	0.02	0.01	0.24	0.08	94.4	
Homemaker	0.27	(0.23,0.30)	0.02	0.14	0.42	0.08	85.8	
Unemployed and looking for a job	0.23	(0.19,0.28)	0.02	0.11	0.47	0.10	92.5	
None of these/other	0.38	(0.30,0.47)	0.04	0.14	0.85	0.20	96.6	
Education								< .001**
Up to 8 years	0.29	(0.25,0.32)	0.02	0.18	0.42	0.07	84.0	
9–15 years	0.19	(0.16,0.22)	0.02	0.09	0.31	0.07	88.3	
16 + years	0.14	(0.12,0.17)	0.01	0.07	0.27	0.05	84.7	
Religious service attendance								< .001**
> 1/week	0.23	(0.20,0.27)	0.02	0.09	0.38	0.08	88.2	
1/week	0.21	(0.18,0.24)	0.01	0.12	0.32	0.06	83.2	
1–3/month	0.21	(0.18,0.24)	0.01	0.11	0.32	0.06	81.6	
A few times a year	0.19	(0.17,0.22)	0.01	0.12	0.34	0.06	82.7	
Never	0.22	(0.19,0.25)	0.02	0.13	0.39	0.07	84.7	
Immigration status								0.027*
Born in this country	0.21	(0.18,0.23)	0.01	0.13	0.33	0.06	82.6	
Born in another country	0.21	(0.19,0.23)	0.01	0.14	0.31	0.05	76.9	
Overall	0.21	(0.18,0.24)	0.01	0.13	0.31	0.06	99.0	

*N* = 202,898

*p < .05

**p < .007 (Bonferroni corrected threshold)

ǂGroup is very small (< 0.1% of the observed sample) within several countries leading to large uncertainty in this estimate—be cautious about interpreting this estimate

^1^*SE analog* = CI Width/4, *CI* = confidence interval, *SE* = standard error, *LL* = lower limits of prediction interval, *UL* = upper limit of prediction interval, prediction interval is the range of likely values of the effect for a randomly selected country; τ is the standard deviation of the distribution of proportions across countries, which is an indicator of cross-national heterogeneity; is an estimate of the proportion of variability in means due to heterogeneity across countries vs. sampling variability; the Global *p*-value corresponds to a test of the null hypothesis that there are no differences between the groups for that sociodemographic characteristic in all of the 22 countries or territories; and additional details of heterogeneity of estimates are available in the forest plots in Additional file 1

Comments on variation by country are found in the “ Discussion” section, with tables available in the additional file [see Additional file 1].

## Discussion

The Global Flourishing Study is a longitudinal study that seeks to understand human flourishing across countries and cultures. Flourishing has been defined as living in a state in which all aspects of a person’s life are good, including domains such as happiness and life satisfaction, mental and physical health, meaning and purpose, character and virtue, close social relationships, and financial and material stability [3]. The question of focus for this paper addresses the domain of mental and physical health: “Do you have any health problems that prevent you from doing any of the things people your age normally can do?” Health problems are a ubiquitous aspect of being human. Most people will experience some form of illness, injury, disability, disease, or other chronic condition preventing activities of daily living at some point in their lives [6]. The presence of health problems hinders participation in everyday life activities typical of one’s community and culture [44, 45]. Understanding the extent of these problems globally and the associated factors can help inform health-related policies and practices to support individuals experiencing health-related limitations. In this study, we examined the prevalence of and variations in the presence of self-reported health problems preventing activities of daily living among residents of 22 geographically and culturally diverse countries.

The prevalence of self-reported health problems varied across countries, ranging from proportions of 0.13 in Poland to 0.34 in the Philippines. Although the study did not ask each person about the specific activities they were kept from doing, the disabling effect of these problems prevented their involvement in normative peer experiences (e.g., employment, schooling, community involvement, daily living). This is particularly striking, as these cross-sectional data address only current experiences of health challenges. Other studies have also highlighted the ubiquity of health-related problems around the world in connection with disease [46], disability [47, 48], or chronic health conditions [49, 50]. The United Kingdom and Germany also had higher proportions of 0.3 or higher. In other studies, Germany’s proportion of individuals experiencing limitations was not as high as reported in these data [51]. In another cross-national study, health inequalities played a significant role in the variation of health limitations between high-income and lower-income countries, which needs to be taken into consideration [19].

Across (and within) countries, several demographic factors showed variations in reported health problems, including age, gender, years of education, marital status, and immigration status between subgroups. The impact of age was especially pronounced. Older participants (age 70 and above) reported health problems three times more often than younger participants (below age 30). The effects of aging on daily activities have been well-documented [52]. Moreover, aging likely accounts for some of the variation related to employment status (i.e., being retired versus working) and marital status (i.e., being widowed versus married). However, it is important to note that even among young people, nearly one in seven reported health problems that limit their everyday functioning. This finding is supported in the literature [12] and highlights the importance of effective health promotion and support across the entire lifespan.

Recognizing that health problems can emerge across the lifespan [53], the proportion of global citizens who must navigate these issues at some time in their lives may be much higher. The potential pathways between health-related problems and limited participation are intriguing to consider. Some health problems—such as certain illnesses and physical limitations—can make direct involvement in particular activities difficult or impossible [54, 55]. In other cases, the stigma associated with particular conditions or diseases may hinder participation [4]. In some cultures or contexts, fear and prejudice can discourage or prohibit involvement in community-based activities. For example, negative attitudes toward certain mental health disorders, epilepsy, HIV/AIDS, and chronic conditions or neglected diseases can close off opportunities for participation [56, 57]. Likewise, limited accessibility or support within public spaces or programs can reduce the involvement of people whose health problems impact mobility [58, 59]. In other words, health-related barriers can emerge from within the person and/or the environment. This highlights the importance of both medical- and community-focused interventions.

Education and employment status also showed variation in self-reported health problems. Health problems were the lowest among people who were currently working or attending school. There was substantially higher unemployment among those who were unemployed and looking for a job (23%), those who were homemakers (27%), and those who were otherwise not working (38%). Although these findings are cross-sectional, the impact of health problems on workforce participation is well documented in the literature [60, 61]. Such studies find that individuals who experience chronic illness, disability, injury, or disease often encounter more difficulties finding and maintaining employment. Likewise, the economic and social impact of absence from the workforce may, in turn, elevate risk for mental or physical health-related problems [62].

Health problems varied by religious service attendance, though much more modest. The highest proportions of health problems were evident among those who attended more than once per week (23%) and those who never attended (22%). It may be that some people are drawn to religious services in response to their health problems (e.g., seeking healing, care, or comfort), while others are prevented from participating in religious services because of their health problems (e.g., difficulties going or getting there, community stigma, disillusionment with religion). How religious beliefs and practices relate to health issues is complex and can vary widely across traditions and health conditions [63, 64].

The prevalence of health-related problems across all 22 countries highlights the global relevance of this issue. At the same time, variation was evident across these countries (range, 13% to 32%). Health problems tended to be more commonly reported in countries marked by greater economic resources or happen to be more individualistic, including the United Kingdom, Germany, Australia, Sweden, and the USA. In contrast, some countries categorized as lower-middle income, collectivist countries including Nigeria, Kenya, and Tanzania, reported the lowest rates. Several issues may contribute to these variations in self-reported health problems. Variation in reporting rates could be due to longer life expectancies, variations in awareness and detection of health-related issues across countries, uneven access to healthcare services, different conceptions of what is considered a “health problem,” different reference points regarding what “people your age normally do,” or the stigma attached to disclosing such problems. Higher-income countries tend to have higher rates of sedentary lifestyles that could contribute to a higher prevalence of health problems and functional limitations [47, 65, 66]. Such variations should prompt more focused inquiry into country-specific variations. A higher economic status may provide improved community support, educational attainment, and healthcare access, mitigating the impacts of health conditions on daily functioning. However, a higher life expectancy increases the number of individuals with health problems requiring support.

In additional file 1 (Tables S1a-S22b), we observe variation among demographic factors by country. Gender and religious service attendance showed the most variation between countries. Females, overall, had a higher proportion of health limitations except in Hong Kong, Japan, and the Philippines, where males had higher proportions. Reasons may be related to gender-based disparities in social norms and policies related to healthy aging in countries with a gender difference. Religious service attendance did not appear to influence health problems in Australia, Brazil, Germany, Indonesia, Nigeria, the Philippines, and Turkey. Interestingly, these countries vary regarding primary religion and overall distribution of religious affiliation. Understanding the complex interaction of religious beliefs, cultural values, and social structure is important to identify potential pathways between religious service attendance and self-reported health problems.

Hong Kong showed a different age pattern with the lowest rate among 50–59-year-olds (0.16; 95% CI 0.13, 0.20) relative to the other age groups, including the youngest group of 18–24-year-olds (0.18; 95% CI 0.13, 0.22). In Hong Kong, separated individuals had almost double the rate of health problems compared to all other categories (0.83; 95% CI 0.58, 1.00). Japan and Turkey showed little variation by age until age 60 and above. Even among older age categories, rates in Japan were strikingly low.

The global variation in health problems preventing individuals from participating in activities expected for people their age is critical to human flourishing. Understanding the differences in how such problems are perceived, associated factors contributing to health problems, and changes over time will lead to a better application of social, cultural, economic, and spiritual determinants.

Several limitations of this study should be noted. First, using a dichotomous survey question, the study addressed self-reported health problems preventing normal activities. We are unable to speak to the nature of the issue(s) each person experienced, the severity of its impact, or its permanence. Future studies should explore the ways and extent to which different health problems impact participation in normative activities. Second, we explored a limited number of factors that might account for variations in health problems within and across countries. Although our focus here was on personal demographic factors, it is also important to explore the ways in which variations in policies, resources, systems, healthcare services, attitudes, cultures, environments, and myriad other factors shape health outcomes. Indeed, myriad factors—including personal, environmental, societal, and other factors not addressed in this study—can combine to account for variations in health limitations. Third, generalizability is limited to those countries included in the study, though the countries included represent over 60% of the world’s population [18]. Fourth, our cross-sectional data are purely descriptive and should not be interpreted causally. Fifth, selection bias is likely given that individuals with health limitations may be less likely to participate in population-based surveys [67]. Lastly, educational attainment measured as years of education may not be comparable across countries, given differences in education levels. Future waves of data from the GFS will enable causal examination. Finally, caution is needed in interpreting cross-national differences as these may be influenced by matters of translation, different modes of assessment, differing interpretations of items and response scales, and seasonal effects arising from data being collected in different countries at different times of the year.

## Conclusions

This study describes the distribution of health problems preventing individuals from doing activities normal for others their age. Healthcare access and availability, social support, stigma, and policy influence the variation observed across countries. Age is a driving factor and related to the influence of education and employment associated with health problems. Healthy aging and providing necessary support for individuals as they age are critical in promoting global human flourishing.

## Supplementary Information

Additional file 1. Table S1a: Nationally representative descriptive statistics for Argentina. Table S1b: Proportions by demographic category for Argentina. Table S2a: Nationally representative descriptive statistics for Australia. Table S2b: Proportions by demographic category for Australia. Table S3a: Nationally representative descriptive statistics for Brazil. Table S3b: Proportions by demographic category for Brazil. Table S4a: Nationally representative descriptive statistics for Egypt. Table S4b: Proportions by demographic category for Egypt. Table S5a: Nationally representative descriptive statistics for Germany. Table S5b: Proportions by demographic category for Germany. Table S6a: Nationally representative descriptive statistics for Hong Kong. Table S6b: Proportions by demographic category for Hong Kong. Table S7a: Nationally representative descriptive statistics for India. Table S7b: Proportions by demographic category for India. Table S8a: Nationally representative descriptive statistics for Indonesia. Table S8b: Proportions by demographic category for Indonesia. Table S9a: Nationally representative descriptive statistics for Israel. Table S9b: Proportions by demographic category for Israel. Table S10a: Nationally representative descriptive statistics for Japan. Table S10b: Proportions by demographic category for Japan. Table S11a: Nationally representative descriptive statistics for Kenya. Table S11b: Proportions by demographic category for Kenya. Table S12a: Nationally representative descriptive statistics for Mexico. Table S12b: Proportions by demographic category for Mexico. Table S13a: Nationally representative descriptive statistics for Nigeria. Table S13b: Proportions by demographic category for Nigeria. Table S14a: Nationally representative descriptive statistics for the Philippines. Table S14b: Proportions by demographic category for Philippines. Table S15a: Nationally representative descriptive statistics for Poland. Table S15b: Proportions by demographic category for Poland. Table S16a: Nationally representative descriptive statistics for South Africa. Table S16b: Proportions by demographic category for South Africa. Table S17a: Nationally representative descriptive statistics for Spain. Table S17b: Proportions by demographic category for Spain. Table S18a: Nationally representative descriptive statistics for Sweden. Table S18b: Proportions by demographic category for Sweden. Table S19a: Nationally representative descriptive statistics for Tanzania. Table S19b: Proportions by demographic category for Tanzania. Table S20a: Nationally representative descriptive statistics for Turkey. Table S20b: Proportions by demographic category for Turkey. Table S21a: Nationally representative descriptive statistics for United Kingdom. Table S21b: Proportions by demographic category for United Kingdom. Table S22a: Nationally representative descriptive statistics for United States. Table S22b: Proportions by demographic category for United States. Table S23: Population weighted meta-analysis of results demographic group means. Figure S1: Forest plot for ‘Age group’ – ’18-24’. Figure S2: Forest plot for ‘Age group’ – ’25-29’. Figure S3: Forest plot for ‘Age group’ – ’30-39’. Figure S4: Forest plot for ‘Age group’ – ’40-49’. Figure S5: Forest plot for ‘Age group’ – ’50-59’. Figure S6: Forest plot for ‘Age group’ –’60-69’. Figure S7: Forest plot for ‘Age group’ – ’70-79’. Figure S8: Forest plot for ‘Age group’ – ’80 and older’. Figure S9: Forest plot for ‘Gender’ –‘Male’. Figure S10: Forest plot for ‘Gender’ – ‘Female. Figure S11: Forest plot for ‘Gender’ – ‘Other’. Figure S12: Forest plot for ‘Marital status’ –‘Married’. Figure S13: Forest plot for ‘Marital status’ – ‘Separated’. Figure S14: Forest plot for ‘Marital status’ – ‘Divorced’. Figure S15: Forest plot for‘Marital status’ – ‘Widowed’. Figure S16: Forest plot for ‘Marital status’— ‘Single, never married’. Figure S17: Forest plot for ‘Marital status’— ‘Domestic partner’. Figure S18: Forest plot for ‘Employment status’—‘Employed for an employer’. Figure S19: Forest plot for ‘Employment status’— ‘Self-employed’. Figure S20: Forest plot for ‘Employment status’ – ‘Retired’. Figure S21: Forest plot for ‘Employment status’ –‘Student’. Figure S22: Forest plot for ‘Employment status’ – ‘Homemaker’. Figure S23: Forest plot for ‘Employment status’ – ‘Unemployed and looking for a job’. Figure S24: Forest plot for ‘Employment status’—‘None of these/other’. Figure S25: Forest plot for ‘Religious service attendance’— ‘<1/week’. Figure S26: Forest plot for ‘Religious service attendance’—‘1 /week’. Figure S27: Forest plot for ‘Religious service attendance’—‘1–3 /month’. Figure S28: Forest plot for ‘Religious service attendance’— ‘A few times a year’. Figure S29: Forest plot for ‘Religious service attendance’ – ‘Never’. Figure S30: Forest plot for ‘Education’ – ‘Up to 8 years’. Figure S31: Forest plot for ‘Education’ – ‘9-15 years’. Figure S32: Forest plot for ‘Education’ – ’16+ years’. Figure S33: Forest plot for‘Immigration status’—‘Born in this country’. Figure S34: Forest plot for ‘Immigration status’—‘Born in another country’.

Additional file 2. STROBE checklist.

## Data Availability

All analyses were pre-registered through the Center for Open Science Open Science Framework prior to data access (https://osf.io/uk9ay) [25] The analysis code used to conduct the analysis is openly available in an online repository through the Center for Open Science (https://osf.io/vbype) [24]. The data used in this study are available through the Center for Open Science (https://www.cos.io/gfs) [28]. The dataset used was Wave 1 non-sensitive Global data available February 2024 - March 2026 via preregistration and publicly from then onwards [27].

## Abbreviations

COS: Center for Open Science
GFS: Global Flourishing Study
STROBE: Strengthening the Reporting of Observational Studies in Epidemiology
TRAPD: Translation, Review, Adjudication, Pretest, Documentation
