# Supplementary material for: A cross-sectional study of demographic variation in health problem-related limitations in life across 22 countries: a cross-national analysis
Source: BMC Glob Public Health. 2025 Sep 3;3:78. doi: 10.1186/s44263-025-00190-6 (PMC12406540; doi:10.1186/s44263-025-00190-6)

**GFS Online Supplement**

**Important Notes and Caveats**

This online supplement to the Global Flourishing Study paper reporting demographic variation in *health problems* has several important caveats to interpretation. First, estimating the within country group proportions can be unstable if the group size is small (<1%) of the country sample size. In such cases, the uncertainty in the estimate leads to a multiple imputation adjusted degrees of freedom less than 1. This means there is not enough information to evaluate the uncertainty in the estimate. We flagged such cases with a “*”. Secondly, comparing results across countries should be done with caution due to possible measurement non-invariance and differences in translation.

***Table S1a. Nationally representative descriptive statistics for Argentina***

| **Characteristic** | **N = 6,724**^1^ |
| --- | --- |
| **Age group** |  |
| 18-24 | 1,108 (16%) |
| 25-29 | 719 (11%) |
| 30-39 | 1,432 (21%) |
| 40-49 | 1,254 (19%) |
| 50-59 | 1,014 (15%) |
| 60-69 | 730 (11%) |
| 70-79 | 356 (5.3%) |
| 80 or older | 112 (1.7%) |
| (Missing) | 0 (0%) |
| **Gender** |  |
| Male | 3,143 (47%) |
| Female | 3,542 (53%) |
| Other | 21 (0.3%) |
| (Missing) | 18 (0.3%) |
| **Marital status** |  |
| Married | 1,565 (23%) |
| Separated | 455 (6.8%) |
| Divorced | 321 (4.8%) |
| Widowed | 401 (6.0%) |
| Never | 2,381 (35%) |
| Domestic Partner | 1,514 (23%) |
| (Missing) | 88 (1.3%) |
| **Employment** |  |
| Employed for an employer | 2,440 (36%) |
| Self-employed | 1,748 (26%) |
| Retired | 773 (11%) |
| Student | 354 (5.3%) |
| Homemaker | 639 (9.5%) |
| Unemployed and looking for a job | 569 (8.5%) |
| None of these/Other | 179 (2.7%) |
| (Missing) | 22 (0.3%) |
| **Religious service attendance** |  |
| At least 1/week | 532 (7.9%) |
| 1/week | 773 (12%) |
| 1-3/month | 461 (6.8%) |
| A few times a year | 1,949 (29%) |
| Never | 2,982 (44%) |
| (Missing) | 27 (0.4%) |
| **Education** |  |
| Up to 8 years | 2,263 (34%) |
| 9-15 years | 3,823 (57%) |
| 16+ years | 635 (9.4%) |
| (Missing) | 3 (<0.1%) |
| **Immigration** |  |
| Born in this country | 6,346 (94%) |
| Born in another country | 348 (5.2%) |
| (Missing) | 29 (0.4%) |
| **Religious affiliation** |  |
| Christianity | 4,992 (74%) |
| Islam | 9 (0.1%) |
| Hinduism | 6 (<0.1%) |
| Buddhism | 35 (0.5%) |
| Judaism | 40 (0.6%) |
| Sikhism | 0 (<0.1%) |
| Baha'i | 0 (0%) |
| Jainism | 0 (0%) |
| Shinto | 0 (0%) |
| Taoism | 2 (<0.1%) |
| Confucianism | 0 (<0.1%) |
| Primal, Animist, or Folk religion | 19 (0.3%) |
| Spiritism | 0 (0%) |
| Umbanda, Candomble, and other African-derived religions | 0 (0%) |
| Chinese folk/traditional religion | 0 (0%) |
| Some other religion | 156 (2.3%) |
| No religion/Atheist/Agnostic | 1,352 (20%) |
| (Missing) | 111 (1.7%) |
| **Race/Ethnicity** |  |
| Asian | 43 (0.6%) |
| Black | 95 (1.4%) |
| Indigenous | 129 (1.9%) |
| Mestizo(a) | 1,801 (27%) |
| Mullato(a) | 75 (1.1%) |
| Other | 104 (1.5%) |
| White | 3,406 (51%) |
| (Missing) | 1,070 (16%) |
| ^1^n (%) | |

***Table S1b. Proportions by demographic category for Argentina***

| Variable | Category | Mean | 95% CI | SE | Global p-value |
| --- | --- | --- | --- | --- | --- |
| Age group | 18-24 | 0.14 | (0.11, 0.17) | 0.02 | < .001 |
|  | 25-29 | 0.12 | (0.09, 0.16) | 0.02 |  |
|  | 30-39 | 0.13 | (0.10, 0.15) | 0.01 |  |
|  | 40-49 | 0.16 | (0.13, 0.19) | 0.01 |  |
|  | 50-59 | 0.25 | (0.21, 0.28) | 0.02 |  |
|  | 60-69 | 0.29 | (0.24, 0.35) | 0.03 |  |
|  | 70-79 | 0.36 | (0.28, 0.43) | 0.04 |  |
|  | 80 or older | 0.42 | (0.27, 0.57) | 0.08 |  |
| Gender | Male | 0.16 | (0.14, 0.18) | 0.01 | < .001 |
|  | Female | 0.21 | (0.19, 0.23) | 0.01 |  |
|  | Other | 0.62 | (0.34, 0.90) | 0.13 |  |
| Marital status | Married | 0.21 | (0.18, 0.23) | 0.01 | < .001 |
|  | Separated | 0.27 | (0.21, 0.33) | 0.03 |  |
|  | Divorced | 0.23 | (0.17, 0.29) | 0.03 |  |
|  | Widowed | 0.31 | (0.23, 0.38) | 0.04 |  |
|  | Never | 0.16 | (0.13, 0.18) | 0.01 |  |
|  | Domestic Partner | 0.15 | (0.13, 0.18) | 0.01 |  |
| Employment | Employed for an employer | 0.11 | (0.09, 0.13) | 0.01 | < .001 |
|  | Self-employed | 0.16 | (0.13, 0.18) | 0.01 |  |
|  | Retired | 0.39 | (0.34, 0.44) | 0.03 |  |
|  | Student | 0.16 | (0.11, 0.22) | 0.03 |  |
|  | Homemaker | 0.23 | (0.18, 0.28) | 0.02 |  |
|  | Unemployed and looking for a job | 0.22 | (0.17, 0.27) | 0.03 |  |
|  | None of these/Other | 0.43 | (0.33, 0.53) | 0.05 |  |
| Religious service attendance | At least 1/week | 0.23 | (0.17, 0.29) | 0.03 | 0.003 |
|  | 1/week | 0.20 | (0.16, 0.25) | 0.02 |  |
|  | 1-3/month | 0.28 | (0.22, 0.34) | 0.03 |  |
|  | A few times a year | 0.17 | (0.14, 0.19) | 0.01 |  |
|  | Never | 0.17 | (0.15, 0.19) | 0.01 |  |
| Education | Up to 8 years | 0.25 | (0.22, 0.28) | 0.02 | < .001 |
|  | 9-15 years | 0.17 | (0.15, 0.18) | 0.01 |  |
|  | 16+ years | 0.09 | (0.07, 0.12) | 0.01 |  |
| Immigration status | Born in this country | 0.19 | (0.17, 0.20) | 0.01 | 0.719 |
|  | Born in another country | 0.18 | (0.12, 0.24) | 0.03 |  |
| Religious affiliation | Christianity | 0.19 | (0.18, 0.21) | 0.01 | < .001 |
|  | Islam | 0.16 | (0.00, 1.00) | 0.26 |  |
|  | Hinduism | 0.59 | * | * |  |
|  | Buddhism | 0.02 | (0.00, 0.08) | 0.02 |  |
|  | Judaism | 0.18 | (0.00, 0.37) | 0.10 |  |
|  | Sikhism | 1.00 | * | * |  |
|  | Taoism | 0.00 | * | * |  |
|  | Confucianism | 1.00 | * | * |  |
|  | Primal, Animist, or Folk religion | 0.10 | (0.00, 0.66) | 0.14 |  |
|  | Some other religion | 0.27 | (0.17, 0.38) | 0.05 |  |
|  | No religion/Atheist/Agnostic | 0.16 | (0.13, 0.19) | 0.01 |  |
| Race/ethnicity | Asian | 0.12 | (0.00, 0.27) | 0.07 | 0.334 |
|  | Black | 0.23 | (0.10, 0.36) | 0.06 |  |
|  | Indigenous | 0.22 | (0.11, 0.34) | 0.06 |  |
|  | Mestizo(a) | 0.17 | (0.14, 0.19) | 0.01 |  |
|  | Mullato(a) | 0.26 | (0.09, 0.42) | 0.08 |  |
|  | White | 0.20 | (0.18, 0.21) | 0.01 |  |
|  | Other | 0.15 | (0.06, 0.24) | 0.05 |  |

***Table S2a. Nationally representative descriptive statistics for Australia***

| **Characteristic** | **N = 3,844**^1^ |
| --- | --- |
| **Age group** |  |
| 18-24 | 345 (9.0%) |
| 25-29 | 282 (7.3%) |
| 30-39 | 641 (17%) |
| 40-49 | 618 (16%) |
| 50-59 | 691 (18%) |
| 60-69 | 589 (15%) |
| 70-79 | 498 (13%) |
| 80 or older | 178 (4.6%) |
| (Missing) | 2 (<0.1%) |
| **Gender** |  |
| Male | 1,861 (48%) |
| Female | 1,941 (50%) |
| Other | 36 (0.9%) |
| (Missing) | 6 (0.2%) |
| **Marital status** |  |
| Married | 1,797 (47%) |
| Separated | 158 (4.1%) |
| Divorced | 332 (8.6%) |
| Widowed | 215 (5.6%) |
| Never | 855 (22%) |
| Domestic Partner | 450 (12%) |
| (Missing) | 38 (1.0%) |
| **Employment** |  |
| Employed for an employer | 1,881 (49%) |
| Self-employed | 380 (9.9%) |
| Retired | 912 (24%) |
| Student | 190 (5.0%) |
| Homemaker | 137 (3.6%) |
| Unemployed and looking for a job | 134 (3.5%) |
| None of these/Other | 206 (5.4%) |
| (Missing) | 4 (0.1%) |
| **Religious service attendance** |  |
| At least 1/week | 162 (4.2%) |
| 1/week | 299 (7.8%) |
| 1-3/month | 135 (3.5%) |
| A few times a year | 656 (17%) |
| Never | 2,584 (67%) |
| (Missing) | 7 (0.2%) |
| **Education** |  |
| Up to 8 years | 70 (1.8%) |
| 9-15 years | 2,434 (63%) |
| 16+ years | 1,330 (35%) |
| (Missing) | 10 (0.3%) |
| **Immigration** |  |
| Born in this country | 2,953 (77%) |
| Born in another country | 885 (23%) |
| (Missing) | 6 (0.2%) |
| **Religious affiliation** |  |
| Christianity | 1,592 (41%) |
| Islam | 45 (1.2%) |
| Hinduism | 31 (0.8%) |
| Buddhism | 36 (0.9%) |
| Judaism | 26 (0.7%) |
| Sikhism | 8 (0.2%) |
| Baha'i | 7 (0.2%) |
| Jainism | 0 (0%) |
| Shinto | 0 (0%) |
| Taoism | 5 (0.1%) |
| Confucianism | 0 (0%) |
| Primal, Animist, or Folk religion | 23 (0.6%) |
| Spiritism | 0 (0%) |
| Umbanda, Candomble, and other African-derived religions | 0 (0%) |
| Chinese folk/traditional religion | 0 (0%) |
| Some other religion | 39 (1.0%) |
| No religion/Atheist/Agnostic | 2,020 (53%) |
| (Missing) | 15 (0.4%) |
| **Race/Ethnicity** |  |
| Aboriginal | 53 (1.4%) |
| Australian | 1,946 (51%) |
| Australian British/European | 1,047 (27%) |
| Chinese | 75 (1.9%) |
| Indian | 58 (1.5%) |
| Japanese | 1 (<0.1%) |
| Malay | 11 (0.3%) |
| New Zealander | 91 (2.4%) |
| Other | 163 (4.2%) |
| Other European | 357 (9.3%) |
| Russian | 7 (0.2%) |
| Samoan | 4 (0.1%) |
| Sinhalese | 1 (<0.1%) |
| Spanish | 2 (<0.1%) |
| Sri Lankan Moor | 1 (<0.1%) |
| Sri Lankan Tamil | 7 (0.2%) |
| Vietnamese | 7 (0.2%) |
| (Missing) | 14 (0.4%) |
| ^1^n (%) | |

***Table S2b. Proportions by demographic category for Australia***

| Variable | Category | Mean | 95% CI | SE | Global p-value |
| --- | --- | --- | --- | --- | --- |
| Age group | 18-24 | 0.16 | (0.10, 0.22) | 0.03 | < .001 |
|  | 25-29 | 0.22 | (0.15, 0.30) | 0.04 |  |
|  | 30-39 | 0.20 | (0.16, 0.25) | 0.02 |  |
|  | 40-49 | 0.24 | (0.20, 0.28) | 0.02 |  |
|  | 50-59 | 0.32 | (0.28, 0.36) | 0.02 |  |
|  | 60-69 | 0.32 | (0.28, 0.36) | 0.02 |  |
|  | 70-79 | 0.39 | (0.34, 0.44) | 0.03 |  |
|  | 80 or older | 0.35 | (0.28, 0.43) | 0.04 |  |
| Gender | Male | 0.23 | (0.21, 0.26) | 0.01 | < .001 |
|  | Female | 0.31 | (0.29, 0.34) | 0.01 |  |
|  | Other | 0.56 | (0.31, 0.80) | 0.12 |  |
| Marital status | Married | 0.25 | (0.22, 0.27) | 0.01 | < .001 |
|  | Separated | 0.33 | (0.23, 0.43) | 0.05 |  |
|  | Divorced | 0.40 | (0.34, 0.47) | 0.03 |  |
|  | Widowed | 0.41 | (0.33, 0.49) | 0.04 |  |
|  | Never | 0.29 | (0.25, 0.33) | 0.02 |  |
|  | Domestic Partner | 0.20 | (0.15, 0.25) | 0.03 |  |
| Employment | Employed for an employer | 0.19 | (0.16, 0.21) | 0.01 | < .001 |
|  | Self-employed | 0.21 | (0.16, 0.26) | 0.02 |  |
|  | Retired | 0.40 | (0.37, 0.44) | 0.02 |  |
|  | Student | 0.23 | (0.14, 0.32) | 0.05 |  |
|  | Homemaker | 0.40 | (0.29, 0.52) | 0.06 |  |
|  | Unemployed and looking for a job | 0.36 | (0.24, 0.47) | 0.06 |  |
|  | None of these/Other | 0.59 | (0.50, 0.68) | 0.05 |  |
| Religious service attendance | At least 1/week | 0.24 | (0.16, 0.32) | 0.04 | 0.422 |
|  | 1/week | 0.31 | (0.25, 0.38) | 0.03 |  |
|  | 1-3/month | 0.26 | (0.17, 0.35) | 0.05 |  |
|  | A few times a year | 0.25 | (0.21, 0.29) | 0.02 |  |
|  | Never | 0.28 | (0.26, 0.30) | 0.01 |  |
| Education | Up to 8 years | 0.23 | (0.08, 0.38) | 0.08 | < .001 |
|  | 9-15 years | 0.32 | (0.30, 0.35) | 0.01 |  |
|  | 16+ years | 0.19 | (0.17, 0.22) | 0.01 |  |
| Immigration status | Born in this country | 0.29 | (0.27, 0.31) | 0.01 | 0.007 |
|  | Born in another country | 0.24 | (0.20, 0.27) | 0.02 |  |
| Religious affiliation | Christianity | 0.30 | (0.28, 0.33) | 0.01 | < .001 |
|  | Islam | 0.09 | (0.00, 0.22) | 0.06 |  |
|  | Hinduism | 0.08 | (0.00, 0.24) | 0.08 |  |
|  | Buddhism | 0.32 | (0.13, 0.50) | 0.09 |  |
|  | Judaism | 0.10 | (0.00, 0.21) | 0.05 |  |
|  | Sikhism | 0.21 | * | * |  |
|  | Baha'i | 0.14 | * | * |  |
|  | Taoism | 0.20 | * | * |  |
|  | Primal, Animist, or Folk religion | 0.62 | (0.29, 0.95) | 0.15 |  |
|  | Some other religion | 0.49 | (0.26, 0.72) | 0.11 |  |
|  | No religion/Atheist/Agnostic | 0.26 | (0.23, 0.28) | 0.01 |  |
| Race/ethnicity | Aboriginal | 0.36 | (0.17, 0.55) | 0.09 | < .001 |
|  | Australian | 0.30 | (0.27, 0.33) | 0.01 |  |
|  | Australian British/European | 0.29 | (0.26, 0.32) | 0.02 |  |
|  | Chinese | 0.06 | (0.00, 0.13) | 0.03 |  |
|  | Indian | 0.05 | (0.00, 0.12) | 0.03 |  |
|  | Japanese | 0.54 | * | * |  |
|  | Malay | 0.00 | * | * |  |
|  | Sinhalese | 0.00 | * | * |  |
|  | Spanish | 0.38 | * | * |  |
|  | Sri Lankan Moor | 0.67 | * | * |  |
|  | Sri Lankan Tamil | 0.36 | * | * |  |
|  | Vietnamese | 0.00 | * | * |  |
|  | Russian | 0.04 | * | * |  |
|  | Samoan | 0.39 | * | * |  |
|  | New Zealander | 0.20 | (0.09, 0.32) | 0.06 |  |
|  | Other European | 0.24 | (0.19, 0.29) | 0.03 |  |
|  | Other | 0.24 | (0.15, 0.32) | 0.05 |  |

***Table S3a. Nationally representative descriptive statistics for Brazil***

| **Characteristic** | **N = 13,204**^1^ |
| --- | --- |
| **Age group** |  |
| 18-24 | 1,986 (15%) |
| 25-29 | 1,468 (11%) |
| 30-39 | 2,908 (22%) |
| 40-49 | 2,638 (20%) |
| 50-59 | 2,131 (16%) |
| 60-69 | 1,435 (11%) |
| 70-79 | 510 (3.9%) |
| 80 or older | 126 (1.0%) |
| (Missing) | 0 (0%) |
| **Gender** |  |
| Male | 6,320 (48%) |
| Female | 6,820 (52%) |
| Other | 35 (0.3%) |
| (Missing) | 30 (0.2%) |
| **Marital status** |  |
| Married | 4,646 (35%) |
| Separated | 594 (4.5%) |
| Divorced | 865 (6.5%) |
| Widowed | 408 (3.1%) |
| Never | 4,347 (33%) |
| Domestic Partner | 2,081 (16%) |
| (Missing) | 263 (2.0%) |
| **Employment** |  |
| Employed for an employer | 3,756 (28%) |
| Self-employed | 2,918 (22%) |
| Retired | 1,536 (12%) |
| Student | 624 (4.7%) |
| Homemaker | 1,305 (9.9%) |
| Unemployed and looking for a job | 2,419 (18%) |
| None of these/Other | 448 (3.4%) |
| (Missing) | 199 (1.5%) |
| **Religious service attendance** |  |
| At least 1/week | 2,386 (18%) |
| 1/week | 2,272 (17%) |
| 1-3/month | 1,398 (11%) |
| A few times a year | 3,978 (30%) |
| Never | 3,110 (24%) |
| (Missing) | 61 (0.5%) |
| **Education** |  |
| Up to 8 years | 3,139 (24%) |
| 9-15 years | 7,665 (58%) |
| 16+ years | 2,390 (18%) |
| (Missing) | 10 (<0.1%) |
| **Immigration** |  |
| Born in this country | 12,688 (96%) |
| Born in another country | 153 (1.2%) |
| (Missing) | 363 (2.7%) |
| **Religious affiliation** |  |
| Christianity | 9,911 (75%) |
| Islam | 6 (<0.1%) |
| Hinduism | 1 (<0.1%) |
| Buddhism | 37 (0.3%) |
| Judaism | 31 (0.2%) |
| Sikhism | 0 (0%) |
| Baha'i | 2 (<0.1%) |
| Jainism | 2 (<0.1%) |
| Shinto | 1 (<0.1%) |
| Taoism | 1 (<0.1%) |
| Confucianism | 6 (<0.1%) |
| Primal, Animist, or Folk religion | 15 (0.1%) |
| Spiritism | 696 (5.3%) |
| Umbanda, Candomble, and other African-derived religions | 525 (4.0%) |
| Chinese folk/traditional religion | 0 (0%) |
| Some other religion | 144 (1.1%) |
| No religion/Atheist/Agnostic | 1,712 (13%) |
| (Missing) | 113 (0.9%) |
| **Race/Ethnicity** |  |
| Amarela | 238 (1.8%) |
| Branca | 5,169 (39%) |
| Indígena | 131 (1.0%) |
| Other | 61 (0.5%) |
| Parda | 5,125 (39%) |
| Preta | 1,615 (12%) |
| (Missing) | 865 (6.6%) |
| ^1^n (%) | |

***Table S3b. Proportions by demographic category for Brazil***

| Variable | Category | Mean | 95% CI | SE | Global p-value |
| --- | --- | --- | --- | --- | --- |
| Age group | 18-24 | 0.12 | (0.10, 0.14) | 0.01 | < .001 |
|  | 25-29 | 0.12 | (0.10, 0.14) | 0.01 |  |
|  | 30-39 | 0.14 | (0.12, 0.15) | 0.01 |  |
|  | 40-49 | 0.21 | (0.19, 0.23) | 0.01 |  |
|  | 50-59 | 0.26 | (0.23, 0.29) | 0.01 |  |
|  | 60-69 | 0.29 | (0.26, 0.33) | 0.02 |  |
|  | 70-79 | 0.32 | (0.25, 0.40) | 0.04 |  |
|  | 80 or older | 0.33 | (0.20, 0.47) | 0.07 |  |
| Gender | Male | 0.19 | (0.18, 0.20) | 0.01 | 0.721 |
|  | Female | 0.19 | (0.18, 0.21) | 0.01 |  |
|  | Other | 0.24 | (0.07, 0.41) | 0.08 |  |
| Marital status | Married | 0.18 | (0.16, 0.19) | 0.01 | < .001 |
|  | Separated | 0.25 | (0.20, 0.30) | 0.03 |  |
|  | Divorced | 0.26 | (0.22, 0.31) | 0.02 |  |
|  | Widowed | 0.30 | (0.24, 0.37) | 0.03 |  |
|  | Never | 0.18 | (0.16, 0.20) | 0.01 |  |
|  | Domestic Partner | 0.19 | (0.16, 0.21) | 0.01 |  |
| Employment | Employed for an employer | 0.11 | (0.10, 0.13) | 0.01 | < .001 |
|  | Self-employed | 0.16 | (0.14, 0.18) | 0.01 |  |
|  | Retired | 0.35 | (0.31, 0.39) | 0.02 |  |
|  | Student | 0.14 | (0.11, 0.17) | 0.02 |  |
|  | Homemaker | 0.24 | (0.21, 0.27) | 0.02 |  |
|  | Unemployed and looking for a job | 0.21 | (0.19, 0.23) | 0.01 |  |
|  | None of these/Other | 0.37 | (0.31, 0.43) | 0.03 |  |
| Religious service attendance | At least 1/week | 0.20 | (0.17, 0.22) | 0.01 | 0.639 |
|  | 1/week | 0.21 | (0.18, 0.23) | 0.01 |  |
|  | 1-3/month | 0.19 | (0.17, 0.22) | 0.01 |  |
|  | A few times a year | 0.18 | (0.17, 0.20) | 0.01 |  |
|  | Never | 0.19 | (0.17, 0.21) | 0.01 |  |
| Education | Up to 8 years | 0.29 | (0.27, 0.31) | 0.01 | < .001 |
|  | 9-15 years | 0.17 | (0.15, 0.18) | 0.01 |  |
|  | 16+ years | 0.15 | (0.13, 0.17) | 0.01 |  |
| Immigration status | Born in this country | 0.19 | (0.18, 0.20) | 0.00 | 0.094 |
|  | Born in another country | 0.28 | (0.18, 0.38) | 0.05 |  |
| Religious affiliation | Christianity | 0.19 | (0.18, 0.20) | 0.01 | < .001 |
|  | Islam | 0.47 | * | * |  |
|  | Hinduism | 0.00 | * | * |  |
|  | Buddhism | 0.23 | (0.01, 0.45) | 0.11 |  |
|  | Judaism | 0.25 | (0.00, 0.51) | 0.12 |  |
|  | Baha'i | 0.70 | * | * |  |
|  | Jainism | 0.47 | * | * |  |
|  | Shinto | 0.00 | * | * |  |
|  | Taoism | 0.24 | * | * |  |
|  | Confucianism | 0.07 | * | * |  |
|  | Primal, Animist, or Folk religion | 0.29 | * | * |  |
|  | Spiritism | 0.21 | (0.17, 0.25) | 0.02 |  |
|  | Umbanda, Candomble, and other African-derived religions | 0.19 | (0.15, 0.23) | 0.02 |  |
|  | Some other religion | 0.22 | (0.12, 0.32) | 0.05 |  |
|  | No religion/Atheist/Agnostic | 0.17 | (0.15, 0.20) | 0.01 |  |
| Race/ethnicity | Branca | 0.20 | (0.18, 0.21) | 0.01 | 0.017 |
|  | Preta | 0.16 | (0.14, 0.19) | 0.01 |  |
|  | Parda | 0.19 | (0.18, 0.21) | 0.01 |  |
|  | Amarela | 0.28 | (0.20, 0.35) | 0.04 |  |
|  | Indígena | 0.21 | (0.13, 0.30) | 0.04 |  |
|  | Other | 0.29 | (0.11, 0.46) | 0.09 |  |

***Table S4a. Nationally representative descriptive statistics for Egypt***

| **Characteristic** | **N = 4,729**^1^ |
| --- | --- |
| **Age group** |  |
| 18-24 | 960 (20%) |
| 25-29 | 607 (13%) |
| 30-39 | 1,204 (25%) |
| 40-49 | 897 (19%) |
| 50-59 | 613 (13%) |
| 60-69 | 387 (8.2%) |
| 70-79 | 54 (1.1%) |
| 80 or older | 7 (0.2%) |
| (Missing) | 0 (0%) |
| **Gender** |  |
| Male | 2,394 (51%) |
| Female | 2,334 (49%) |
| Other | 0 (0%) |
| (Missing) | 0 (<0.1%) |
| **Marital status** |  |
| Married | 3,387 (72%) |
| Separated | 39 (0.8%) |
| Divorced | 101 (2.1%) |
| Widowed | 238 (5.0%) |
| Never | 947 (20%) |
| Domestic Partner | 0 (0%) |
| (Missing) | 17 (0.4%) |
| **Employment** |  |
| Employed for an employer | 1,267 (27%) |
| Self-employed | 892 (19%) |
| Retired | 253 (5.4%) |
| Student | 297 (6.3%) |
| Homemaker | 1,772 (37%) |
| Unemployed and looking for a job | 224 (4.7%) |
| None of these/Other | 21 (0.4%) |
| (Missing) | 3 (<0.1%) |
| **Religious service attendance** |  |
| At least 1/week | 839 (18%) |
| 1/week | 960 (20%) |
| 1-3/month | 368 (7.8%) |
| A few times a year | 458 (9.7%) |
| Never | 2,091 (44%) |
| (Missing) | 12 (0.3%) |
| **Education** |  |
| Up to 8 years | 2,486 (53%) |
| 9-15 years | 1,599 (34%) |
| 16+ years | 643 (14%) |
| (Missing) | 1 (<0.1%) |
| **Immigration** |  |
| Born in this country | 4,713 (100%) |
| Born in another country | 16 (0.3%) |
| (Missing) | 1 (<0.1%) |
| **Religious affiliation** |  |
| Christianity | 120 (2.5%) |
| Islam | 4,607 (97%) |
| Hinduism | 0 (0%) |
| Buddhism | 0 (0%) |
| Judaism | 0 (0%) |
| Sikhism | 0 (0%) |
| Baha'i | 0 (0%) |
| Jainism | 0 (0%) |
| Shinto | 0 (0%) |
| Taoism | 0 (<0.1%) |
| Confucianism | 0 (0%) |
| Primal, Animist, or Folk religion | 0 (0%) |
| Spiritism | 0 (0%) |
| Umbanda, Candomble, and other African-derived religions | 0 (0%) |
| Chinese folk/traditional religion | 0 (0%) |
| Some other religion | 0 (0%) |
| No religion/Atheist/Agnostic | 0 (0%) |
| (Missing) | 1 (<0.1%) |
| **Race/Ethnicity** |  |
| Arab | 4,585 (97%) |
| Bedouin Arab | 4 (<0.1%) |
| Greek | 1 (<0.1%) |
| Nubian | 27 (0.6%) |
| Turkish | 9 (0.2%) |
| (Missing) | 102 (2.2%) |
| ^1^n (%) | |

***Table S4b. Proportions by demographic category for Egypt***

| Variable | Category | Mean | 95% CI | SE | Global p-value |
| --- | --- | --- | --- | --- | --- |
| Age group | 18-24 | 0.13 | (0.10, 0.16) | 0.02 | < .001 |
|  | 25-29 | 0.12 | (0.09, 0.15) | 0.02 |  |
|  | 30-39 | 0.21 | (0.18, 0.24) | 0.01 |  |
|  | 40-49 | 0.30 | (0.27, 0.34) | 0.02 |  |
|  | 50-59 | 0.41 | (0.37, 0.45) | 0.02 |  |
|  | 60-69 | 0.48 | (0.41, 0.56) | 0.04 |  |
|  | 70-79 | 0.62 | (0.47, 0.78) | 0.08 |  |
|  | 80 or older | 1.00 | * | * |  |
| Gender | Male | 0.21 | (0.19, 0.23) | 0.01 | < .001 |
|  | Female | 0.30 | (0.28, 0.32) | 0.01 |  |
| Marital status | Married | 0.28 | (0.26, 0.30) | 0.01 | < .001 |
|  | Separated | 0.26 | (0.06, 0.46) | 0.10 |  |
|  | Divorced | 0.34 | (0.24, 0.44) | 0.05 |  |
|  | Widowed | 0.49 | (0.42, 0.57) | 0.04 |  |
|  | Never | 0.10 | (0.07, 0.12) | 0.01 |  |
| Employment | Employed for an employer | 0.20 | (0.17, 0.23) | 0.01 | < .001 |
|  | Self-employed | 0.20 | (0.17, 0.24) | 0.02 |  |
|  | Retired | 0.52 | (0.44, 0.60) | 0.04 |  |
|  | Student | 0.07 | (0.03, 0.10) | 0.02 |  |
|  | Homemaker | 0.32 | (0.29, 0.34) | 0.01 |  |
|  | Unemployed and looking for a job | 0.18 | (0.11, 0.25) | 0.04 |  |
|  | None of these/Other | 0.26 | (0.04, 0.49) | 0.10 |  |
| Religious service attendance | At least 1/week | 0.21 | (0.18, 0.25) | 0.02 | 0.003 |
|  | 1/week | 0.25 | (0.21, 0.29) | 0.02 |  |
|  | 1-3/month | 0.25 | (0.19, 0.31) | 0.03 |  |
|  | A few times a year | 0.19 | (0.14, 0.24) | 0.03 |  |
|  | Never | 0.29 | (0.26, 0.31) | 0.01 |  |
| Education | Up to 8 years | 0.32 | (0.30, 0.35) | 0.01 | < .001 |
|  | 9-15 years | 0.18 | (0.16, 0.20) | 0.01 |  |
|  | 16+ years | 0.16 | (0.13, 0.19) | 0.02 |  |
| Immigration status | Born in this country | 0.25 | (0.24, 0.27) | 0.01 | 0.465 |
|  | Born in another country | 0.17 | (0.00, 0.42) | 0.12 |  |
| Religious affiliation | Christianity | 0.27 | (0.19, 0.36) | 0.04 | < .001 |
|  | Islam | 0.25 | (0.24, 0.27) | 0.01 |  |
|  | Taoism | 1.00 | * | * |  |
| Race/ethnicity | Arab | 0.25 | (0.24, 0.27) | 0.01 | 0.598 |
|  | Turkish | 0.15 | (0.00, 0.46) | 0.09 |  |
|  | Greek | 0.00 | * | * |  |
|  | Bedouin Arab | 0.00 | * | * |  |
|  | Nubian | 0.35 | (0.21, 0.49) | 0.07 |  |

***Table S5a. Nationally representative descriptive statistics for Germany***

| **Characteristic** | **N = 9,506**^1^ |
| --- | --- |
| **Age group** |  |
| 18-24 | 829 (8.7%) |
| 25-29 | 774 (8.1%) |
| 30-39 | 1,438 (15%) |
| 40-49 | 1,494 (16%) |
| 50-59 | 1,729 (18%) |
| 60-69 | 1,915 (20%) |
| 70-79 | 1,137 (12%) |
| 80 or older | 190 (2.0%) |
| (Missing) | 0 (0%) |
| **Gender** |  |
| Male | 4,641 (49%) |
| Female | 4,843 (51%) |
| Other | 11 (0.1%) |
| (Missing) | 11 (0.1%) |
| **Marital status** |  |
| Married | 4,784 (50%) |
| Separated | 219 (2.3%) |
| Divorced | 767 (8.1%) |
| Widowed | 409 (4.3%) |
| Never | 2,627 (28%) |
| Domestic Partner | 619 (6.5%) |
| (Missing) | 81 (0.9%) |
| **Employment** |  |
| Employed for an employer | 4,950 (52%) |
| Self-employed | 712 (7.5%) |
| Retired | 2,480 (26%) |
| Student | 605 (6.4%) |
| Homemaker | 251 (2.6%) |
| Unemployed and looking for a job | 288 (3.0%) |
| None of these/Other | 204 (2.1%) |
| (Missing) | 14 (0.2%) |
| **Religious service attendance** |  |
| At least 1/week | 285 (3.0%) |
| 1/week | 424 (4.5%) |
| 1-3/month | 550 (5.8%) |
| A few times a year | 2,362 (25%) |
| Never | 5,876 (62%) |
| (Missing) | 9 (<0.1%) |
| **Education** |  |
| Up to 8 years | 235 (2.5%) |
| 9-15 years | 6,094 (64%) |
| 16+ years | 3,164 (33%) |
| (Missing) | 13 (0.1%) |
| **Immigration** |  |
| Born in this country | 8,722 (92%) |
| Born in another country | 744 (7.8%) |
| (Missing) | 40 (0.4%) |
| **Religious affiliation** |  |
| Christianity | 5,052 (53%) |
| Islam | 351 (3.7%) |
| Hinduism | 12 (0.1%) |
| Buddhism | 51 (0.5%) |
| Judaism | 19 (0.2%) |
| Sikhism | 5 (<0.1%) |
| Baha'i | 3 (<0.1%) |
| Jainism | 0 (0%) |
| Shinto | 2 (<0.1%) |
| Taoism | 0 (<0.1%) |
| Confucianism | 4 (<0.1%) |
| Primal, Animist, or Folk religion | 34 (0.4%) |
| Spiritism | 0 (0%) |
| Umbanda, Candomble, and other African-derived religions | 0 (0%) |
| Chinese folk/traditional religion | 0 (0%) |
| Some other religion | 60 (0.6%) |
| No religion/Atheist/Agnostic | 3,815 (40%) |
| (Missing) | 99 (1.0%) |
| ^1^n (%) | |

***Table S5b. Proportions by demographic category for Germany***

| Variable | Category | Mean | 95% CI | SE | Global p-value |
| --- | --- | --- | --- | --- | --- |
| Age group | 18-24 | 0.21 | (0.17, 0.25) | 0.02 | < .001 |
|  | 25-29 | 0.20 | (0.17, 0.24) | 0.02 |  |
|  | 30-39 | 0.20 | (0.18, 0.22) | 0.01 |  |
|  | 40-49 | 0.28 | (0.25, 0.31) | 0.01 |  |
|  | 50-59 | 0.32 | (0.29, 0.35) | 0.01 |  |
|  | 60-69 | 0.37 | (0.35, 0.40) | 0.01 |  |
|  | 70-79 | 0.39 | (0.35, 0.43) | 0.02 |  |
|  | 80 or older | 0.35 | (0.27, 0.44) | 0.04 |  |
| Gender | Male | 0.29 | (0.27, 0.30) | 0.01 | 0.256 |
|  | Female | 0.30 | (0.29, 0.32) | 0.01 |  |
|  | Other | 0.37 | (0.00, 1.00) | 0.22 |  |
| Marital status | Married | 0.30 | (0.28, 0.31) | 0.01 | < .001 |
|  | Separated | 0.31 | (0.24, 0.38) | 0.04 |  |
|  | Divorced | 0.39 | (0.35, 0.43) | 0.02 |  |
|  | Widowed | 0.41 | (0.35, 0.48) | 0.03 |  |
|  | Never | 0.26 | (0.24, 0.28) | 0.01 |  |
|  | Domestic Partner | 0.24 | (0.20, 0.28) | 0.02 |  |
| Employment | Employed for an employer | 0.22 | (0.21, 0.24) | 0.01 | < .001 |
|  | Self-employed | 0.27 | (0.23, 0.31) | 0.02 |  |
|  | Retired | 0.42 | (0.40, 0.45) | 0.01 |  |
|  | Student | 0.18 | (0.14, 0.22) | 0.02 |  |
|  | Homemaker | 0.43 | (0.36, 0.50) | 0.04 |  |
|  | Unemployed and looking for a job | 0.48 | (0.41, 0.55) | 0.04 |  |
|  | None of these/Other | 0.47 | (0.39, 0.56) | 0.04 |  |
| Religious service attendance | At least 1/week | 0.26 | (0.19, 0.32) | 0.03 | 0.148 |
|  | 1/week | 0.26 | (0.20, 0.31) | 0.03 |  |
|  | 1-3/month | 0.26 | (0.21, 0.31) | 0.03 |  |
|  | A few times a year | 0.30 | (0.28, 0.32) | 0.01 |  |
|  | Never | 0.30 | (0.29, 0.32) | 0.01 |  |
| Education | Up to 8 years | 0.40 | (0.32, 0.47) | 0.04 | < .001 |
|  | 9-15 years | 0.32 | (0.31, 0.34) | 0.01 |  |
|  | 16+ years | 0.24 | (0.22, 0.26) | 0.01 |  |
| Immigration status | Born in this country | 0.30 | (0.29, 0.31) | 0.01 | 0.008 |
|  | Born in another country | 0.24 | (0.20, 0.28) | 0.02 |  |
| Religious affiliation | Christianity | 0.31 | (0.29, 0.32) | 0.01 | < .001 |
|  | Islam | 0.22 | (0.16, 0.28) | 0.03 |  |
|  | Hinduism | 0.32 | * | * |  |
|  | Buddhism | 0.18 | (0.06, 0.31) | 0.06 |  |
|  | Judaism | 0.28 | (0.00, 0.56) | 0.10 |  |
|  | Sikhism | 0.17 | * | * |  |
|  | Baha'i | 0.00 | * | * |  |
|  | Shinto | 0.00 | * | * |  |
|  | Taoism | 0.00 | * | * |  |
|  | Confucianism | 0.41 | * | * |  |
|  | Primal, Animist, or Folk religion | 0.42 | (0.16, 0.67) | 0.12 |  |
|  | Some other religion | 0.47 | (0.28, 0.67) | 0.10 |  |
|  | No religion/Atheist/Agnostic | 0.29 | (0.27, 0.31) | 0.01 |  |

***Table S6a. Nationally representative descriptive statistics for Hong Kong***

| **Characteristic** | **N = 3,012**^1^ |
| --- | --- |
| **Age group** |  |
| 18-24 | 217 (7.2%) |
| 25-29 | 198 (6.6%) |
| 30-39 | 507 (17%) |
| 40-49 | 580 (19%) |
| 50-59 | 711 (24%) |
| 60-69 | 620 (21%) |
| 70-79 | 164 (5.5%) |
| 80 or older | 15 (0.5%) |
| (Missing) | 0 (0%) |
| **Gender** |  |
| Male | 1,390 (46%) |
| Female | 1,620 (54%) |
| Other | 2 (<0.1%) |
| (Missing) | 0 (0%) |
| **Marital status** |  |
| Married | 2,080 (69%) |
| Separated | 21 (0.7%) |
| Divorced | 105 (3.5%) |
| Widowed | 45 (1.5%) |
| Never | 723 (24%) |
| Domestic Partner | 37 (1.2%) |
| (Missing) | 1 (<0.1%) |
| **Employment** |  |
| Employed for an employer | 2,056 (68%) |
| Self-employed | 245 (8.1%) |
| Retired | 423 (14%) |
| Student | 55 (1.8%) |
| Homemaker | 114 (3.8%) |
| Unemployed and looking for a job | 62 (2.0%) |
| None of these/Other | 39 (1.3%) |
| (Missing) | 18 (0.6%) |
| **Religious service attendance** |  |
| At least 1/week | 237 (7.9%) |
| 1/week | 567 (19%) |
| 1-3/month | 332 (11%) |
| A few times a year | 543 (18%) |
| Never | 1,332 (44%) |
| (Missing) | 1 (<0.1%) |
| **Education** |  |
| Up to 8 years | 433 (14%) |
| 9-15 years | 2,031 (67%) |
| 16+ years | 547 (18%) |
| (Missing) | 0 (0%) |
| **Immigration** |  |
| Born in this country | 2,637 (88%) |
| Born in another country | 321 (11%) |
| (Missing) | 53 (1.8%) |
| **Religious affiliation** |  |
| Christianity | 757 (25%) |
| Islam | 86 (2.8%) |
| Hinduism | 20 (0.7%) |
| Buddhism | 349 (12%) |
| Judaism | 10 (0.3%) |
| Sikhism | 2 (<0.1%) |
| Baha'i | 3 (<0.1%) |
| Jainism | 1 (<0.1%) |
| Shinto | 19 (0.6%) |
| Taoism | 97 (3.2%) |
| Confucianism | 11 (0.4%) |
| Primal, Animist, or Folk religion | 27 (0.9%) |
| Spiritism | 0 (0%) |
| Umbanda, Candomble, and other African-derived religions | 0 (0%) |
| Chinese folk/traditional religion | 106 (3.5%) |
| Some other religion | 4 (0.1%) |
| No religion/Atheist/Agnostic | 1,518 (50%) |
| (Missing) | 5 (0.2%) |
| **Race/Ethnicity** |  |
| Chinese (Cantonese) | 1,930 (64%) |
| Chinese (Chaoshan) | 201 (6.7%) |
| Chinese (Fujianese) | 117 (3.9%) |
| Chinese (Hakka) | 121 (4.0%) |
| Chinese (Other ethnicity) | 264 (8.8%) |
| Chinese (Shanghainese) | 89 (2.9%) |
| East Asian (Korean, Japanese) | 10 (0.3%) |
| Other | 4 (0.1%) |
| South Asian (Indian, Nepalese, Pakistani) | 17 (0.6%) |
| Southeast Asian (Filipino, Indonesian, Thailand) | 46 (1.5%) |
| Taiwanese | 14 (0.4%) |
| White | 15 (0.5%) |
| (Missing) | 184 (6.1%) |
| ^1^n (%) | |

***Table S6b. Proportions by demographic category for Hong Kong***

| Variable | Category | Mean | 95% CI | SE | Global p-value |
| --- | --- | --- | --- | --- | --- |
| Age group | 18-24 | 0.18 | (0.13, 0.22) | 0.02 | < .001 |
|  | 25-29 | 0.25 | (0.17, 0.33) | 0.04 |  |
|  | 30-39 | 0.23 | (0.19, 0.27) | 0.02 |  |
|  | 40-49 | 0.21 | (0.16, 0.25) | 0.02 |  |
|  | 50-59 | 0.16 | (0.13, 0.20) | 0.02 |  |
|  | 60-69 | 0.20 | (0.15, 0.26) | 0.03 |  |
|  | 70-79 | 0.36 | (0.20, 0.52) | 0.08 |  |
|  | 80 or older | 0.00 | * | * |  |
| Gender | Male | 0.22 | (0.19, 0.25) | 0.02 | < .001 |
|  | Female | 0.20 | (0.17, 0.22) | 0.01 |  |
|  | Other | 0.00 | * | * |  |
| Marital status | Married | 0.19 | (0.17, 0.22) | 0.01 | < .001 |
|  | Separated | 0.83 | (0.58, 1.00) | 0.11 |  |
|  | Divorced | 0.47 | (0.30, 0.63) | 0.08 |  |
|  | Widowed | 0.42 | (0.12, 0.71) | 0.14 |  |
|  | Never | 0.18 | (0.15, 0.21) | 0.02 |  |
|  | Domestic Partner | 0.25 | (0.00, 0.49) | 0.12 |  |
| Employment | Employed for an employer | 0.17 | (0.15, 0.19) | 0.01 | < .001 |
|  | Self-employed | 0.37 | (0.29, 0.46) | 0.04 |  |
|  | Retired | 0.24 | (0.15, 0.32) | 0.04 |  |
|  | Student | 0.10 | (0.03, 0.16) | 0.03 |  |
|  | Homemaker | 0.33 | (0.18, 0.47) | 0.07 |  |
|  | Unemployed and looking for a job | 0.23 | (0.08, 0.37) | 0.07 |  |
|  | None of these/Other | 0.57 | (0.31, 0.83) | 0.13 |  |
| Religious service attendance | At least 1/week | 0.39 | (0.32, 0.46) | 0.03 | < .001 |
|  | 1/week | 0.28 | (0.23, 0.34) | 0.03 |  |
|  | 1-3/month | 0.21 | (0.15, 0.28) | 0.03 |  |
|  | A few times a year | 0.18 | (0.13, 0.23) | 0.03 |  |
|  | Never | 0.15 | (0.12, 0.18) | 0.02 |  |
| Education | Up to 8 years | 0.34 | (0.25, 0.43) | 0.04 | < .001 |
|  | 9-15 years | 0.20 | (0.18, 0.22) | 0.01 |  |
|  | 16+ years | 0.13 | (0.09, 0.17) | 0.02 |  |
| Immigration status | Born in this country | 0.20 | (0.18, 0.22) | 0.01 | 0.122 |
|  | Born in another country | 0.27 | (0.18, 0.36) | 0.05 |  |
| Religious affiliation | Christianity | 0.24 | (0.19, 0.28) | 0.02 | < .001 |
|  | Islam | 0.55 | (0.38, 0.72) | 0.09 |  |
|  | Hinduism | 0.60 | (0.12, 1.00) | 0.15 |  |
|  | Buddhism | 0.33 | (0.27, 0.40) | 0.03 |  |
|  | Judaism | 0.50 | * | * |  |
|  | Sikhism | 0.00 | * | * |  |
|  | Baha'i | 0.82 | * | * |  |
|  | Jainism | 1.00 | * | * |  |
|  | Shinto | 0.03 | (0.00, 0.17) | 0.03 |  |
|  | Taoism | 0.18 | (0.06, 0.29) | 0.06 |  |
|  | Confucianism | 0.30 | * | * |  |
|  | Primal, Animist, or Folk religion | 0.23 | (0.04, 0.41) | 0.08 |  |
|  | Chinese folk/traditional religion | 0.18 | (0.08, 0.28) | 0.05 |  |
|  | Some other religion | 0.41 | * | * |  |
|  | No religion/Atheist/Agnostic | 0.14 | (0.11, 0.17) | 0.01 |  |
| Race/ethnicity | White | 0.47 | (0.00, 1.00) | 0.20 | < .001 |
|  | Other | 0.07 | * | * |  |
|  | Chinese (Cantonese) | 0.20 | (0.18, 0.23) | 0.01 |  |
|  | Chinese (Chaoshan) | 0.28 | (0.20, 0.36) | 0.04 |  |
|  | Chinese (Fujianese) | 0.22 | (0.12, 0.33) | 0.05 |  |
|  | Chinese (Hakka) | 0.24 | (0.13, 0.35) | 0.06 |  |
|  | Chinese (Shanghainese) | 0.27 | (0.10, 0.45) | 0.09 |  |
|  | Chinese (Other ethnicity) | 0.09 | (0.04, 0.14) | 0.02 |  |
|  | East Asian (Korean, Japanese) | 0.75 | (0.00, 1.00) | 0.17 |  |
|  | Southeast Asian (Filipino, Indonesian, Thailand) | 0.31 | (0.03, 0.58) | 0.13 |  |
|  | South Asian (Indian, Nepalese, Pakistani) | 0.19 | (0.00, 0.73) | 0.16 |  |
|  | Taiwanese | 0.14 | * | * |  |

***Table S7a. Nationally representative descriptive statistics for India***

| **Characteristic** | **N = 12,765**^1^ |
| --- | --- |
| **Age group** |  |
| 18-24 | 2,543 (20%) |
| 25-29 | 1,640 (13%) |
| 30-39 | 3,109 (24%) |
| 40-49 | 2,275 (18%) |
| 50-59 | 1,574 (12%) |
| 60-69 | 1,188 (9.3%) |
| 70-79 | 370 (2.9%) |
| 80 or older | 67 (0.5%) |
| (Missing) | 0 (0%) |
| **Gender** |  |
| Male | 6,473 (51%) |
| Female | 6,292 (49%) |
| Other | 0 (0%) |
| (Missing) | 0 (0%) |
| **Marital status** |  |
| Married | 9,848 (77%) |
| Separated | 45 (0.4%) |
| Divorced | 25 (0.2%) |
| Widowed | 445 (3.5%) |
| Never | 2,065 (16%) |
| Domestic Partner | 269 (2.1%) |
| (Missing) | 69 (0.5%) |
| **Employment** |  |
| Employed for an employer | 2,660 (21%) |
| Self-employed | 3,401 (27%) |
| Retired | 286 (2.2%) |
| Student | 532 (4.2%) |
| Homemaker | 4,221 (33%) |
| Unemployed and looking for a job | 902 (7.1%) |
| None of these/Other | 715 (5.6%) |
| (Missing) | 48 (0.4%) |
| **Religious service attendance** |  |
| At least 1/week | 2,875 (23%) |
| 1/week | 3,166 (25%) |
| 1-3/month | 2,740 (21%) |
| A few times a year | 2,090 (16%) |
| Never | 1,823 (14%) |
| (Missing) | 71 (0.6%) |
| **Education** |  |
| Up to 8 years | 11,422 (89%) |
| 9-15 years | 1,194 (9.4%) |
| 16+ years | 145 (1.1%) |
| (Missing) | 4 (<0.1%) |
| **Immigration** |  |
| Born in this country | 12,629 (99%) |
| Born in another country | 110 (0.9%) |
| (Missing) | 26 (0.2%) |
| **Religious affiliation** |  |
| Christianity | 306 (2.4%) |
| Islam | 1,555 (12%) |
| Hinduism | 10,362 (81%) |
| Buddhism | 230 (1.8%) |
| Judaism | 0 (0%) |
| Sikhism | 127 (1.0%) |
| Baha'i | 0 (0%) |
| Jainism | 10 (<0.1%) |
| Shinto | 1 (<0.1%) |
| Taoism | 0 (0%) |
| Confucianism | 0 (0%) |
| Primal, Animist, or Folk religion | 30 (0.2%) |
| Spiritism | 0 (0%) |
| Umbanda, Candomble, and other African-derived religions | 0 (0%) |
| Chinese folk/traditional religion | 0 (0%) |
| Some other religion | 67 (0.5%) |
| No religion/Atheist/Agnostic | 13 (0.1%) |
| (Missing) | 62 (0.5%) |
| **Race/Ethnicity** |  |
| General | 3,538 (28%) |
| Other backward caste | 4,177 (33%) |
| Schedule caste | 3,599 (28%) |
| Schedule tribe | 1,185 (9.3%) |
| (Missing) | 267 (2.1%) |
| ^1^n (%) | |

***Table S7b. Proportions by demographic category for India***

| Variable | Category | Mean | 95% CI | SE | Global p-value |
| --- | --- | --- | --- | --- | --- |
| Age group | 18-24 | 0.18 | (0.16, 0.21) | 0.01 | < .001 |
|  | 25-29 | 0.22 | (0.20, 0.24) | 0.01 |  |
|  | 30-39 | 0.25 | (0.23, 0.27) | 0.01 |  |
|  | 40-49 | 0.32 | (0.30, 0.34) | 0.01 |  |
|  | 50-59 | 0.36 | (0.33, 0.39) | 0.02 |  |
|  | 60-69 | 0.43 | (0.40, 0.47) | 0.02 |  |
|  | 70-79 | 0.46 | (0.39, 0.52) | 0.03 |  |
|  | 80 or older | 0.40 | (0.24, 0.56) | 0.08 |  |
| Gender | Male | 0.26 | (0.25, 0.27) | 0.01 | < .001 |
|  | Female | 0.31 | (0.29, 0.32) | 0.01 |  |
| Marital status | Married | 0.29 | (0.28, 0.31) | 0.01 | < .001 |
|  | Separated | 0.26 | (0.09, 0.42) | 0.08 |  |
|  | Divorced | 0.33 | (0.06, 0.61) | 0.13 |  |
|  | Widowed | 0.48 | (0.42, 0.54) | 0.03 |  |
|  | Never | 0.19 | (0.16, 0.21) | 0.01 |  |
|  | Domestic Partner | 0.34 | (0.24, 0.44) | 0.05 |  |
| Employment | Employed for an employer | 0.28 | (0.26, 0.30) | 0.01 | < .001 |
|  | Self-employed | 0.25 | (0.23, 0.27) | 0.01 |  |
|  | Retired | 0.48 | (0.40, 0.56) | 0.04 |  |
|  | Student | 0.17 | (0.14, 0.21) | 0.02 |  |
|  | Homemaker | 0.30 | (0.28, 0.32) | 0.01 |  |
|  | Unemployed and looking for a job | 0.28 | (0.25, 0.32) | 0.02 |  |
|  | None of these/Other | 0.37 | (0.32, 0.42) | 0.03 |  |
| Religious service attendance | At least 1/week | 0.30 | (0.28, 0.33) | 0.01 | 0.007 |
|  | 1/week | 0.29 | (0.27, 0.31) | 0.01 |  |
|  | 1-3/month | 0.27 | (0.26, 0.29) | 0.01 |  |
|  | A few times a year | 0.25 | (0.23, 0.27) | 0.01 |  |
|  | Never | 0.29 | (0.26, 0.31) | 0.01 |  |
| Education | Up to 8 years | 0.29 | (0.28, 0.30) | 0.01 | < .001 |
|  | 9-15 years | 0.24 | (0.21, 0.26) | 0.01 |  |
|  | 16+ years | 0.22 | (0.15, 0.28) | 0.03 |  |
| Immigration status | Born in this country | 0.28 | (0.27, 0.30) | 0.01 | 0.269 |
|  | Born in another country | 0.23 | (0.13, 0.33) | 0.05 |  |
| Religious affiliation | Christianity | 0.34 | (0.26, 0.41) | 0.04 | < .001 |
|  | Islam | 0.26 | (0.22, 0.30) | 0.02 |  |
|  | Hinduism | 0.29 | (0.28, 0.30) | 0.01 |  |
|  | Buddhism | 0.22 | (0.16, 0.28) | 0.03 |  |
|  | Sikhism | 0.28 | (0.19, 0.37) | 0.05 |  |
|  | Jainism | 0.23 | * | * |  |
|  | Shinto | 0.00 | * | * |  |
|  | Primal, Animist, or Folk religion | 0.16 | (0.02, 0.29) | 0.06 |  |
|  | Some other religion | 0.17 | (0.04, 0.29) | 0.06 |  |
|  | No religion/Atheist/Agnostic | 0.36 | (0.00, 1.00) | 0.19 |  |
| Race/ethnicity | General | 0.27 | (0.25, 0.29) | 0.01 | 0.054 |
|  | Other backward caste | 0.30 | (0.28, 0.32) | 0.01 |  |
|  | Schedule caste | 0.29 | (0.27, 0.31) | 0.01 |  |
|  | Schedule tribe | 0.25 | (0.22, 0.28) | 0.02 |  |

***Table S8a. Nationally representative descriptive statistics for Indonesia***

| **Characteristic** | **N = 6,992**^1^ |
| --- | --- |
| **Age group** |  |
| 18-24 | 1,216 (17%) |
| 25-29 | 849 (12%) |
| 30-39 | 1,591 (23%) |
| 40-49 | 1,576 (23%) |
| 50-59 | 1,169 (17%) |
| 60-69 | 490 (7.0%) |
| 70-79 | 83 (1.2%) |
| 80 or older | 17 (0.2%) |
| (Missing) | 0 (0%) |
| **Gender** |  |
| Male | 3,461 (50%) |
| Female | 3,513 (50%) |
| Other | 7 (<0.1%) |
| (Missing) | 11 (0.2%) |
| **Marital status** |  |
| Married | 4,846 (69%) |
| Separated | 81 (1.2%) |
| Divorced | 196 (2.8%) |
| Widowed | 425 (6.1%) |
| Never | 1,381 (20%) |
| Domestic Partner | 18 (0.3%) |
| (Missing) | 45 (0.6%) |
| **Employment** |  |
| Employed for an employer | 1,323 (19%) |
| Self-employed | 2,187 (31%) |
| Retired | 78 (1.1%) |
| Student | 272 (3.9%) |
| Homemaker | 2,138 (31%) |
| Unemployed and looking for a job | 529 (7.6%) |
| None of these/Other | 448 (6.4%) |
| (Missing) | 18 (0.3%) |
| **Religious service attendance** |  |
| At least 1/week | 2,667 (38%) |
| 1/week | 2,529 (36%) |
| 1-3/month | 786 (11%) |
| A few times a year | 659 (9.4%) |
| Never | 332 (4.8%) |
| (Missing) | 18 (0.3%) |
| **Education** |  |
| Up to 8 years | 3,079 (44%) |
| 9-15 years | 3,491 (50%) |
| 16+ years | 419 (6.0%) |
| (Missing) | 2 (<0.1%) |
| **Immigration** |  |
| Born in this country | 6,958 (100%) |
| Born in another country | 34 (0.5%) |
| (Missing) | 0 (0%) |
| **Religious affiliation** |  |
| Christianity | 504 (7.2%) |
| Islam | 6,406 (92%) |
| Hinduism | 73 (1.0%) |
| Buddhism | 3 (<0.1%) |
| Judaism | 0 (0%) |
| Sikhism | 0 (0%) |
| Baha'i | 0 (0%) |
| Jainism | 0 (0%) |
| Shinto | 0 (0%) |
| Taoism | 1 (<0.1%) |
| Confucianism | 0 (0%) |
| Primal, Animist, or Folk religion | 0 (0%) |
| Spiritism | 0 (0%) |
| Umbanda, Candomble, and other African-derived religions | 0 (0%) |
| Chinese folk/traditional religion | 0 (0%) |
| Some other religion | 1 (<0.1%) |
| No religion/Atheist/Agnostic | 0 (0%) |
| (Missing) | 4 (<0.1%) |
| **Race/Ethnicity** |  |
| Bali | 69 (1.0%) |
| Banjar/Melayu Banjar | 320 (4.6%) |
| Batak | 165 (2.4%) |
| Betawi | 251 (3.6%) |
| Bugis | 243 (3.5%) |
| Jawa | 2,846 (41%) |
| Madura | 262 (3.7%) |
| Makasar | 91 (1.3%) |
| Minangkabau | 273 (3.9%) |
| Other | 1,262 (18%) |
| Sunda/Parahyangan | 1,172 (17%) |
| (Missing) | 38 (0.5%) |
| ^1^n (%) | |

***Table S8b. Proportions by demographic category for Indonesia***

| Variable | Category | Mean | 95% CI | SE | Global p-value |
| --- | --- | --- | --- | --- | --- |
| Age group | 18-24 | 0.12 | (0.10, 0.14) | 0.01 | < .001 |
|  | 25-29 | 0.08 | (0.06, 0.10) | 0.01 |  |
|  | 30-39 | 0.10 | (0.08, 0.12) | 0.01 |  |
|  | 40-49 | 0.15 | (0.12, 0.17) | 0.01 |  |
|  | 50-59 | 0.24 | (0.20, 0.28) | 0.02 |  |
|  | 60-69 | 0.26 | (0.20, 0.32) | 0.03 |  |
|  | 70-79 | 0.40 | (0.23, 0.57) | 0.09 |  |
|  | 80 or older | 0.18 | (0.00, 0.56) | 0.16 |  |
| Gender | Male | 0.15 | (0.14, 0.17) | 0.01 | 0.548 |
|  | Female | 0.15 | (0.13, 0.16) | 0.01 |  |
|  | Other | 0.08 | (0.00, 0.39) | 0.08 |  |
| Marital status | Married | 0.14 | (0.13, 0.16) | 0.01 | < .001 |
|  | Separated | 0.21 | (0.08, 0.35) | 0.07 |  |
|  | Divorced | 0.12 | (0.06, 0.17) | 0.03 |  |
|  | Widowed | 0.27 | (0.22, 0.33) | 0.03 |  |
|  | Never | 0.13 | (0.11, 0.16) | 0.01 |  |
|  | Domestic Partner | 0.38 | (0.03, 0.73) | 0.16 |  |
| Employment | Employed for an employer | 0.13 | (0.10, 0.15) | 0.01 | 0.169 |
|  | Self-employed | 0.15 | (0.13, 0.18) | 0.01 |  |
|  | Retired | 0.17 | (0.09, 0.26) | 0.04 |  |
|  | Student | 0.11 | (0.07, 0.15) | 0.02 |  |
|  | Homemaker | 0.16 | (0.13, 0.18) | 0.01 |  |
|  | Unemployed and looking for a job | 0.18 | (0.13, 0.22) | 0.02 |  |
|  | None of these/Other | 0.14 | (0.10, 0.18) | 0.02 |  |
| Religious service attendance | At least 1/week | 0.14 | (0.12, 0.16) | 0.01 | 0.119 |
|  | 1/week | 0.15 | (0.13, 0.17) | 0.01 |  |
|  | 1-3/month | 0.15 | (0.12, 0.19) | 0.02 |  |
|  | A few times a year | 0.14 | (0.10, 0.17) | 0.02 |  |
|  | Never | 0.23 | (0.16, 0.30) | 0.03 |  |
| Education | Up to 8 years | 0.19 | (0.17, 0.21) | 0.01 | < .001 |
|  | 9-15 years | 0.12 | (0.11, 0.13) | 0.01 |  |
|  | 16+ years | 0.10 | (0.07, 0.13) | 0.02 |  |
| Immigration status | Born in this country | 0.15 | (0.14, 0.16) | 0.01 | 0.108 |
|  | Born in another country | 0.28 | (0.11, 0.46) | 0.08 |  |
| Religious affiliation | Christianity | 0.24 | (0.18, 0.30) | 0.03 | < .001 |
|  | Islam | 0.14 | (0.13, 0.15) | 0.01 |  |
|  | Hinduism | 0.14 | (0.00, 0.28) | 0.07 |  |
|  | Buddhism | 0.00 | * | * |  |
|  | Taoism | 0.00 | * | * |  |
|  | Some other religion | 1.00 | * | * |  |
| Race/ethnicity | Banjar/Melayu Banjar | 0.19 | (0.13, 0.25) | 0.03 | < .001 |
|  | Betawi | 0.12 | (0.07, 0.17) | 0.03 |  |
|  | Bugis | 0.24 | (0.16, 0.31) | 0.04 |  |
|  | Jawa | 0.12 | (0.10, 0.14) | 0.01 |  |
|  | Madura | 0.17 | (0.11, 0.23) | 0.03 |  |
|  | Minangkabau | 0.17 | (0.11, 0.22) | 0.03 |  |
|  | Sunda/Parahyangan | 0.14 | (0.11, 0.17) | 0.02 |  |
|  | Bali | 0.15 | (0.00, 0.30) | 0.08 |  |
|  | Batak | 0.16 | (0.10, 0.21) | 0.03 |  |
|  | Makasar | 0.07 | (0.02, 0.12) | 0.02 |  |
|  | Other | 0.20 | (0.16, 0.24) | 0.02 |  |

***Table S9a. Nationally representative descriptive statistics for Israel***

| **Characteristic** | **N = 3,669**^1^ |
| --- | --- |
| **Age group** |  |
| 18-24 | 553 (15%) |
| 25-29 | 407 (11%) |
| 30-39 | 666 (18%) |
| 40-49 | 616 (17%) |
| 50-59 | 542 (15%) |
| 60-69 | 469 (13%) |
| 70-79 | 336 (9.2%) |
| 80 or older | 79 (2.2%) |
| (Missing) | 0 (0%) |
| **Gender** |  |
| Male | 1,791 (49%) |
| Female | 1,872 (51%) |
| Other | 0 (<0.1%) |
| (Missing) | 6 (0.2%) |
| **Marital status** |  |
| Married | 2,056 (56%) |
| Separated | 48 (1.3%) |
| Divorced | 258 (7.0%) |
| Widowed | 212 (5.8%) |
| Never | 834 (23%) |
| Domestic Partner | 193 (5.3%) |
| (Missing) | 69 (1.9%) |
| **Employment** |  |
| Employed for an employer | 1,793 (49%) |
| Self-employed | 424 (12%) |
| Retired | 576 (16%) |
| Student | 388 (11%) |
| Homemaker | 211 (5.7%) |
| Unemployed and looking for a job | 148 (4.0%) |
| None of these/Other | 118 (3.2%) |
| (Missing) | 10 (0.3%) |
| **Religious service attendance** |  |
| At least 1/week | 649 (18%) |
| 1/week | 495 (14%) |
| 1-3/month | 374 (10%) |
| A few times a year | 1,014 (28%) |
| Never | 1,122 (31%) |
| (Missing) | 14 (0.4%) |
| **Education** |  |
| Up to 8 years | 224 (6.1%) |
| 9-15 years | 1,517 (41%) |
| 16+ years | 1,926 (52%) |
| (Missing) | 2 (<0.1%) |
| **Immigration** |  |
| Born in this country | 2,796 (76%) |
| Born in another country | 868 (24%) |
| (Missing) | 5 (0.1%) |
| **Religious affiliation** |  |
| Christianity | 39 (1.1%) |
| Islam | 656 (18%) |
| Hinduism | 0 (0%) |
| Buddhism | 0 (0%) |
| Judaism | 2,897 (79%) |
| Sikhism | 0 (0%) |
| Baha'i | 2 (<0.1%) |
| Jainism | 0 (0%) |
| Shinto | 0 (0%) |
| Taoism | 1 (<0.1%) |
| Confucianism | 0 (0%) |
| Primal, Animist, or Folk religion | 1 (<0.1%) |
| Spiritism | 0 (0%) |
| Umbanda, Candomble, and other African-derived religions | 0 (0%) |
| Chinese folk/traditional religion | 0 (0%) |
| Some other religion | 5 (0.1%) |
| No religion/Atheist/Agnostic | 64 (1.7%) |
| (Missing) | 4 (0.1%) |
| **Race/Ethnicity** |  |
| Arab | 674 (18%) |
| Jewish | 2,926 (80%) |
| Other | 39 (1.1%) |
| (Missing) | 30 (0.8%) |
| ^1^n (%) | |

***Table S9b. Proportions by demographic category for Israel***

| Variable | Category | Mean | 95% CI | SE | Global p-value |
| --- | --- | --- | --- | --- | --- |
| Age group | 18-24 | 0.06 | (0.04, 0.08) | 0.01 | < .001 |
|  | 25-29 | 0.09 | (0.05, 0.12) | 0.02 |  |
|  | 30-39 | 0.07 | (0.04, 0.09) | 0.01 |  |
|  | 40-49 | 0.10 | (0.07, 0.14) | 0.02 |  |
|  | 50-59 | 0.15 | (0.11, 0.19) | 0.02 |  |
|  | 60-69 | 0.21 | (0.15, 0.27) | 0.03 |  |
|  | 70-79 | 0.35 | (0.27, 0.42) | 0.04 |  |
|  | 80 or older | 0.44 | (0.31, 0.57) | 0.07 |  |
| Gender | Male | 0.13 | (0.10, 0.16) | 0.01 | < .001 |
|  | Female | 0.15 | (0.12, 0.18) | 0.01 |  |
|  | Other | 0.00 | * | * |  |
| Marital status | Married | 0.14 | (0.11, 0.16) | 0.01 | < .001 |
|  | Separated | 0.34 | (0.21, 0.46) | 0.06 |  |
|  | Divorced | 0.16 | (0.10, 0.23) | 0.03 |  |
|  | Widowed | 0.39 | (0.30, 0.47) | 0.04 |  |
|  | Never | 0.07 | (0.05, 0.09) | 0.01 |  |
|  | Domestic Partner | 0.13 | (0.06, 0.20) | 0.04 |  |
| Employment | Employed for an employer | 0.08 | (0.06, 0.11) | 0.01 | < .001 |
|  | Self-employed | 0.11 | (0.07, 0.15) | 0.02 |  |
|  | Retired | 0.30 | (0.24, 0.37) | 0.03 |  |
|  | Student | 0.07 | (0.04, 0.10) | 0.02 |  |
|  | Homemaker | 0.28 | (0.19, 0.37) | 0.04 |  |
|  | Unemployed and looking for a job | 0.13 | (0.06, 0.20) | 0.03 |  |
|  | None of these/Other | 0.24 | (0.15, 0.34) | 0.05 |  |
| Religious service attendance | At least 1/week | 0.08 | (0.06, 0.11) | 0.01 | < .001 |
|  | 1/week | 0.13 | (0.09, 0.17) | 0.02 |  |
|  | 1-3/month | 0.22 | (0.17, 0.28) | 0.03 |  |
|  | A few times a year | 0.16 | (0.12, 0.20) | 0.02 |  |
|  | Never | 0.12 | (0.09, 0.16) | 0.02 |  |
| Education | Up to 8 years | 0.38 | (0.26, 0.51) | 0.06 | < .001 |
|  | 9-15 years | 0.12 | (0.10, 0.15) | 0.01 |  |
|  | 16+ years | 0.12 | (0.09, 0.15) | 0.01 |  |
| Immigration status | Born in this country | 0.12 | (0.10, 0.14) | 0.01 | 0.002 |
|  | Born in another country | 0.21 | (0.15, 0.26) | 0.03 |  |
| Religious affiliation | Christianity | 0.20 | (0.00, 0.40) | 0.10 | 0.016 |
|  | Islam | 0.19 | (0.14, 0.24) | 0.02 |  |
|  | Judaism | 0.13 | (0.10, 0.15) | 0.01 |  |
|  | Baha'i | 0.00 | * | * |  |
|  | Taoism | 0.00 | * | * |  |
|  | Primal, Animist, or Folk religion | 0.00 | * | * |  |
|  | Some other religion | 0.13 | (0.00, 0.37) | 0.12 |  |
|  | No religion/Atheist/Agnostic | 0.14 | (0.04, 0.24) | 0.05 |  |
| Race/ethnicity | Arab | 0.19 | (0.15, 0.24) | 0.02 | 0.019 |
|  | Jewish | 0.13 | (0.10, 0.15) | 0.01 |  |
|  | Other | 0.08 | (0.00, 0.18) | 0.05 |  |

***Table S10a. Nationally representative descriptive statistics for Japan***

| **Characteristic** | **N = 20,543**^1^ |
| --- | --- |
| **Age group** |  |
| 18-24 | 1,589 (7.7%) |
| 25-29 | 806 (3.9%) |
| 30-39 | 2,851 (14%) |
| 40-49 | 3,363 (16%) |
| 50-59 | 3,770 (18%) |
| 60-69 | 4,118 (20%) |
| 70-79 | 3,554 (17%) |
| 80 or older | 493 (2.4%) |
| (Missing) | 0 (0%) |
| **Gender** |  |
| Male | 9,847 (48%) |
| Female | 10,602 (52%) |
| Other | 28 (0.1%) |
| (Missing) | 66 (0.3%) |
| **Marital status** |  |
| Married | 11,837 (58%) |
| Separated | 190 (0.9%) |
| Divorced | 2,126 (10%) |
| Widowed | 1,179 (5.7%) |
| Never | 5,004 (24%) |
| Domestic Partner | 144 (0.7%) |
| (Missing) | 64 (0.3%) |
| **Employment** |  |
| Employed for an employer | 10,853 (53%) |
| Self-employed | 1,748 (8.5%) |
| Retired | 2,535 (12%) |
| Student | 491 (2.4%) |
| Homemaker | 1,276 (6.2%) |
| Unemployed and looking for a job | 622 (3.0%) |
| None of these/Other | 2,983 (15%) |
| (Missing) | 36 (0.2%) |
| **Religious service attendance** |  |
| At least 1/week | 316 (1.5%) |
| 1/week | 348 (1.7%) |
| 1-3/month | 862 (4.2%) |
| A few times a year | 3,112 (15%) |
| Never | 15,788 (77%) |
| (Missing) | 117 (0.6%) |
| **Education** |  |
| Up to 8 years | 567 (2.8%) |
| 9-15 years | 14,893 (72%) |
| 16+ years | 5,083 (25%) |
| (Missing) | 0 (0%) |
| **Immigration** |  |
| Born in this country | 19,548 (95%) |
| Born in another country | 158 (0.8%) |
| (Missing) | 837 (4.1%) |
| **Religious affiliation** |  |
| Christianity | 381 (1.9%) |
| Islam | 10 (<0.1%) |
| Hinduism | 5 (<0.1%) |
| Buddhism | 6,709 (33%) |
| Judaism | 10 (<0.1%) |
| Sikhism | 6 (<0.1%) |
| Baha'i | 2 (<0.1%) |
| Jainism | 11 (<0.1%) |
| Shinto | 469 (2.3%) |
| Taoism | 7 (<0.1%) |
| Confucianism | 17 (<0.1%) |
| Primal, Animist, or Folk religion | 19 (<0.1%) |
| Spiritism | 0 (0%) |
| Umbanda, Candomble, and other African-derived religions | 0 (0%) |
| Chinese folk/traditional religion | 0 (0%) |
| Some other religion | 46 (0.2%) |
| No religion/Atheist/Agnostic | 12,497 (61%) |
| (Missing) | 355 (1.7%) |
| ^1^n (%) | |

***Table S10b. Proportions by demographic category for Japan***

| Variable | Category | Mean | 95% CI | SE | Global p-value |
| --- | --- | --- | --- | --- | --- |
| Age group | 18-24 | 0.16 | (0.14, 0.18) | 0.01 | < .001 |
|  | 25-29 | 0.13 | (0.11, 0.15) | 0.01 |  |
|  | 30-39 | 0.14 | (0.13, 0.15) | 0.01 |  |
|  | 40-49 | 0.17 | (0.15, 0.18) | 0.01 |  |
|  | 50-59 | 0.17 | (0.16, 0.18) | 0.01 |  |
|  | 60-69 | 0.20 | (0.19, 0.21) | 0.01 |  |
|  | 70-79 | 0.21 | (0.19, 0.22) | 0.01 |  |
|  | 80 or older | 0.27 | (0.22, 0.31) | 0.02 |  |
| Gender | Male | 0.18 | (0.18, 0.19) | 0.00 | 0.010 |
|  | Female | 0.17 | (0.16, 0.18) | 0.00 |  |
|  | Other | 0.35 | (0.17, 0.53) | 0.09 |  |
| Marital status | Married | 0.16 | (0.15, 0.17) | 0.00 | < .001 |
|  | Separated | 0.25 | (0.17, 0.33) | 0.04 |  |
|  | Divorced | 0.22 | (0.20, 0.25) | 0.01 |  |
|  | Widowed | 0.21 | (0.18, 0.24) | 0.02 |  |
|  | Never | 0.19 | (0.18, 0.20) | 0.01 |  |
|  | Domestic Partner | 0.23 | (0.14, 0.31) | 0.04 |  |
| Employment | Employed for an employer | 0.14 | (0.13, 0.14) | 0.00 | < .001 |
|  | Self-employed | 0.20 | (0.18, 0.22) | 0.01 |  |
|  | Retired | 0.24 | (0.23, 0.26) | 0.01 |  |
|  | Student | 0.12 | (0.09, 0.15) | 0.01 |  |
|  | Homemaker | 0.21 | (0.18, 0.23) | 0.01 |  |
|  | Unemployed and looking for a job | 0.34 | (0.31, 0.38) | 0.02 |  |
|  | None of these/Other | 0.22 | (0.21, 0.24) | 0.01 |  |
| Religious service attendance | At least 1/week | 0.22 | (0.17, 0.26) | 0.02 | < .001 |
|  | 1/week | 0.30 | (0.24, 0.36) | 0.03 |  |
|  | 1-3/month | 0.24 | (0.20, 0.27) | 0.02 |  |
|  | A few times a year | 0.18 | (0.17, 0.20) | 0.01 |  |
|  | Never | 0.17 | (0.16, 0.18) | 0.00 |  |
| Education | Up to 8 years | 0.26 | (0.22, 0.31) | 0.02 | < .001 |
|  | 9-15 years | 0.18 | (0.17, 0.19) | 0.00 |  |
|  | 16+ years | 0.16 | (0.15, 0.17) | 0.01 |  |
| Immigration status | Born in this country | 0.18 | (0.17, 0.18) | 0.00 | 0.159 |
|  | Born in another country | 0.24 | (0.15, 0.32) | 0.04 |  |
| Religious affiliation | Christianity | 0.25 | (0.19, 0.30) | 0.03 | < .001 |
|  | Islam | 0.71 | * | * |  |
|  | Hinduism | 0.37 | * | * |  |
|  | Buddhism | 0.19 | (0.18, 0.20) | 0.01 |  |
|  | Judaism | 0.10 | * | * |  |
|  | Sikhism | 0.82 | * | * |  |
|  | Baha'i | 0.00 | * | * |  |
|  | Jainism | 0.70 | (0.27, 1.00) | 0.21 |  |
|  | Shinto | 0.23 | (0.19, 0.28) | 0.02 |  |
|  | Taoism | 0.11 | * | * |  |
|  | Confucianism | 0.38 | (0.00, 1.00) | 0.12 |  |
|  | Primal, Animist, or Folk religion | 0.09 | (0.00, 0.32) | 0.07 |  |
|  | Some other religion | 0.19 | (0.05, 0.32) | 0.07 |  |
|  | No religion/Atheist/Agnostic | 0.17 | (0.16, 0.17) | 0.00 |  |

***Table S11a. Nationally representative descriptive statistics for Kenya***

| **Characteristic** | **N = 11,389**^1^ |
| --- | --- |
| **Age group** |  |
| 18-24 | 2,868 (25%) |
| 25-29 | 2,035 (18%) |
| 30-39 | 2,564 (23%) |
| 40-49 | 1,708 (15%) |
| 50-59 | 1,072 (9.4%) |
| 60-69 | 710 (6.2%) |
| 70-79 | 360 (3.2%) |
| 80 or older | 67 (0.6%) |
| (Missing) | 5 (<0.1%) |
| **Gender** |  |
| Male | 5,567 (49%) |
| Female | 5,813 (51%) |
| Other | 2 (<0.1%) |
| (Missing) | 7 (<0.1%) |
| **Marital status** |  |
| Married | 6,626 (58%) |
| Separated | 467 (4.1%) |
| Divorced | 111 (1.0%) |
| Widowed | 464 (4.1%) |
| Never | 3,531 (31%) |
| Domestic Partner | 146 (1.3%) |
| (Missing) | 43 (0.4%) |
| **Employment** |  |
| Employed for an employer | 1,467 (13%) |
| Self-employed | 3,630 (32%) |
| Retired | 319 (2.8%) |
| Student | 1,136 (10.0%) |
| Homemaker | 1,537 (13%) |
| Unemployed and looking for a job | 3,153 (28%) |
| None of these/Other | 138 (1.2%) |
| (Missing) | 9 (<0.1%) |
| **Religious service attendance** |  |
| At least 1/week | 2,774 (24%) |
| 1/week | 6,063 (53%) |
| 1-3/month | 1,219 (11%) |
| A few times a year | 855 (7.5%) |
| Never | 465 (4.1%) |
| (Missing) | 13 (0.1%) |
| **Education** |  |
| Up to 8 years | 4,485 (39%) |
| 9-15 years | 6,115 (54%) |
| 16+ years | 783 (6.9%) |
| (Missing) | 6 (<0.1%) |
| **Immigration** |  |
| Born in this country | 11,270 (99%) |
| Born in another country | 117 (1.0%) |
| (Missing) | 2 (<0.1%) |
| **Religious affiliation** |  |
| Christianity | 10,334 (91%) |
| Islam | 918 (8.1%) |
| Hinduism | 0 (0%) |
| Buddhism | 1 (<0.1%) |
| Judaism | 3 (<0.1%) |
| Sikhism | 0 (0%) |
| Baha'i | 1 (<0.1%) |
| Jainism | 1 (<0.1%) |
| Shinto | 0 (0%) |
| Taoism | 0 (0%) |
| Confucianism | 3 (<0.1%) |
| Primal, Animist, or Folk religion | 7 (<0.1%) |
| Spiritism | 0 (0%) |
| Umbanda, Candomble, and other African-derived religions | 0 (0%) |
| Chinese folk/traditional religion | 0 (0%) |
| Some other religion | 5 (<0.1%) |
| No religion/Atheist/Agnostic | 108 (0.9%) |
| (Missing) | 9 (<0.1%) |
| **Race/Ethnicity** |  |
| Embu | 197 (1.7%) |
| Kalenjin | 1,377 (12%) |
| Kamba | 1,299 (11%) |
| Kenyan Somali/Somali | 396 (3.5%) |
| Kikuyu | 2,119 (19%) |
| Kisii | 789 (6.9%) |
| Luhya | 1,943 (17%) |
| Luo | 1,120 (9.8%) |
| Maasai | 237 (2.1%) |
| Meru | 630 (5.5%) |
| Miji Kenda tribes | 708 (6.2%) |
| Other | 548 (4.8%) |
| (Missing) | 27 (0.2%) |
| ^1^n (%) | |

***Table S11b. Proportions by demographic category for Kenya***

| Variable | Category | Mean | 95% CI | SE | Global p-value |
| --- | --- | --- | --- | --- | --- |
| Age group | 18-24 | 0.09 | (0.07, 0.10) | 0.01 | < .001 |
|  | 25-29 | 0.09 | (0.08, 0.11) | 0.01 |  |
|  | 30-39 | 0.12 | (0.10, 0.14) | 0.01 |  |
|  | 40-49 | 0.17 | (0.15, 0.19) | 0.01 |  |
|  | 50-59 | 0.23 | (0.19, 0.27) | 0.02 |  |
|  | 60-69 | 0.31 | (0.24, 0.38) | 0.03 |  |
|  | 70-79 | 0.42 | (0.34, 0.50) | 0.04 |  |
|  | 80 or older | 0.54 | (0.32, 0.77) | 0.11 |  |
| Gender | Male | 0.12 | (0.11, 0.14) | 0.01 | < .001 |
|  | Female | 0.17 | (0.16, 0.18) | 0.01 |  |
|  | Other | 0.00 | * | * |  |
| Marital status | Married | 0.15 | (0.14, 0.17) | 0.01 | < .001 |
|  | Separated | 0.20 | (0.16, 0.25) | 0.02 |  |
|  | Divorced | 0.20 | (0.10, 0.30) | 0.05 |  |
|  | Widowed | 0.42 | (0.35, 0.50) | 0.04 |  |
|  | Never | 0.09 | (0.08, 0.11) | 0.01 |  |
|  | Domestic Partner | 0.14 | (0.06, 0.22) | 0.04 |  |
| Employment | Employed for an employer | 0.10 | (0.08, 0.11) | 0.01 | < .001 |
|  | Self-employed | 0.15 | (0.13, 0.16) | 0.01 |  |
|  | Retired | 0.39 | (0.30, 0.48) | 0.05 |  |
|  | Student | 0.07 | (0.06, 0.09) | 0.01 |  |
|  | Homemaker | 0.23 | (0.20, 0.26) | 0.02 |  |
|  | Unemployed and looking for a job | 0.13 | (0.11, 0.14) | 0.01 |  |
|  | None of these/Other | 0.32 | (0.22, 0.41) | 0.05 |  |
| Religious service attendance | At least 1/week | 0.18 | (0.16, 0.20) | 0.01 | < .001 |
|  | 1/week | 0.13 | (0.12, 0.15) | 0.01 |  |
|  | 1-3/month | 0.15 | (0.12, 0.17) | 0.01 |  |
|  | A few times a year | 0.14 | (0.11, 0.17) | 0.02 |  |
|  | Never | 0.15 | (0.11, 0.20) | 0.02 |  |
| Education | Up to 8 years | 0.21 | (0.19, 0.23) | 0.01 | < .001 |
|  | 9-15 years | 0.11 | (0.10, 0.12) | 0.01 |  |
|  | 16+ years | 0.08 | (0.06, 0.11) | 0.01 |  |
| Immigration status | Born in this country | 0.15 | (0.14, 0.16) | 0.01 | 0.617 |
|  | Born in another country | 0.17 | (0.09, 0.25) | 0.04 |  |
| Religious affiliation | Christianity | 0.14 | (0.13, 0.15) | 0.01 | 0.008 |
|  | Islam | 0.19 | (0.15, 0.24) | 0.02 |  |
|  | Buddhism | 0.00 | * | * |  |
|  | Judaism | 0.00 | * | * |  |
|  | Baha'i | 0.00 | * | * |  |
|  | Jainism | 0.00 | * | * |  |
|  | Confucianism | 0.00 | * | * |  |
|  | Primal, Animist, or Folk religion | 0.09 | (0.00, 0.28) | 0.09 |  |
|  | Some other religion | 0.37 | * | * |  |
|  | No religion/Atheist/Agnostic | 0.11 | (0.04, 0.17) | 0.03 |  |
| Race/ethnicity | Luhya | 0.18 | (0.15, 0.20) | 0.01 | < .001 |
|  | Luo | 0.23 | (0.19, 0.26) | 0.02 |  |
|  | Kalenjin | 0.09 | (0.07, 0.11) | 0.01 |  |
|  | Kamba | 0.12 | (0.08, 0.15) | 0.02 |  |
|  | Kikuyu | 0.13 | (0.11, 0.15) | 0.01 |  |
|  | Kisii | 0.15 | (0.11, 0.18) | 0.02 |  |
|  | Maasai | 0.03 | (0.01, 0.06) | 0.01 |  |
|  | Meru | 0.14 | (0.11, 0.17) | 0.02 |  |
|  | Kenyan Somali/Somali | 0.18 | (0.09, 0.27) | 0.05 |  |
|  | Miji Kenda tribes | 0.18 | (0.15, 0.22) | 0.02 |  |
|  | Embu | 0.21 | (0.11, 0.31) | 0.05 |  |
|  | Other | 0.13 | (0.09, 0.16) | 0.02 |  |

***Table S12a. Nationally representative descriptive statistics for Mexico***

| **Characteristic** | **N = 5,776**^1^ |
| --- | --- |
| **Age group** |  |
| 18-24 | 986 (17%) |
| 25-29 | 623 (11%) |
| 30-39 | 1,312 (23%) |
| 40-49 | 1,027 (18%) |
| 50-59 | 873 (15%) |
| 60-69 | 611 (11%) |
| 70-79 | 277 (4.8%) |
| 80 or older | 68 (1.2%) |
| (Missing) | 0 (0%) |
| **Gender** |  |
| Male | 2,755 (48%) |
| Female | 2,997 (52%) |
| Other | 3 (<0.1%) |
| (Missing) | 21 (0.4%) |
| **Marital status** |  |
| Married | 2,089 (36%) |
| Separated | 403 (7.0%) |
| Divorced | 230 (4.0%) |
| Widowed | 347 (6.0%) |
| Never | 1,432 (25%) |
| Domestic Partner | 1,109 (19%) |
| (Missing) | 166 (2.9%) |
| **Employment** |  |
| Employed for an employer | 1,921 (33%) |
| Self-employed | 1,091 (19%) |
| Retired | 386 (6.7%) |
| Student | 247 (4.3%) |
| Homemaker | 1,257 (22%) |
| Unemployed and looking for a job | 564 (9.8%) |
| None of these/Other | 169 (2.9%) |
| (Missing) | 141 (2.4%) |
| **Religious service attendance** |  |
| At least 1/week | 609 (11%) |
| 1/week | 1,261 (22%) |
| 1-3/month | 676 (12%) |
| A few times a year | 2,054 (36%) |
| Never | 1,134 (20%) |
| (Missing) | 43 (0.7%) |
| **Education** |  |
| Up to 8 years | 1,291 (22%) |
| 9-15 years | 3,180 (55%) |
| 16+ years | 1,304 (23%) |
| (Missing) | 1 (<0.1%) |
| **Immigration** |  |
| Born in this country | 5,517 (96%) |
| Born in another country | 108 (1.9%) |
| (Missing) | 151 (2.6%) |
| **Religious affiliation** |  |
| Christianity | 4,844 (84%) |
| Islam | 2 (<0.1%) |
| Hinduism | 3 (<0.1%) |
| Buddhism | 6 (0.1%) |
| Judaism | 7 (0.1%) |
| Sikhism | 0 (0%) |
| Baha'i | 1 (<0.1%) |
| Jainism | 1 (<0.1%) |
| Shinto | 2 (<0.1%) |
| Taoism | 4 (<0.1%) |
| Confucianism | 1 (<0.1%) |
| Primal, Animist, or Folk religion | 20 (0.3%) |
| Spiritism | 0 (0%) |
| Umbanda, Candomble, and other African-derived religions | 0 (0%) |
| Chinese folk/traditional religion | 0 (0%) |
| Some other religion | 41 (0.7%) |
| No religion/Atheist/Agnostic | 770 (13%) |
| (Missing) | 75 (1.3%) |
| **Race/Ethnicity** |  |
| Black | 108 (1.9%) |
| Indigenous | 594 (10%) |
| Mestizo | 2,762 (48%) |
| Mulatto | 63 (1.1%) |
| Other | 339 (5.9%) |
| White | 1,116 (19%) |
| (Missing) | 794 (14%) |
| ^1^n (%) | |

***Table S12b. Proportions by demographic category for Mexico***

| Variable | Category | Mean | 95% CI | SE | Global p-value |
| --- | --- | --- | --- | --- | --- |
| Age group | 18-24 | 0.08 | (0.06, 0.10) | 0.01 | < .001 |
|  | 25-29 | 0.10 | (0.07, 0.13) | 0.01 |  |
|  | 30-39 | 0.09 | (0.07, 0.11) | 0.01 |  |
|  | 40-49 | 0.18 | (0.15, 0.21) | 0.02 |  |
|  | 50-59 | 0.23 | (0.20, 0.27) | 0.02 |  |
|  | 60-69 | 0.36 | (0.30, 0.41) | 0.03 |  |
|  | 70-79 | 0.42 | (0.33, 0.51) | 0.04 |  |
|  | 80 or older | 0.45 | (0.26, 0.63) | 0.09 |  |
| Gender | Male | 0.15 | (0.13, 0.16) | 0.01 | < .001 |
|  | Female | 0.20 | (0.18, 0.22) | 0.01 |  |
|  | Other | 0.23 | * | * |  |
| Marital status | Married | 0.19 | (0.16, 0.21) | 0.01 | < .001 |
|  | Separated | 0.22 | (0.16, 0.27) | 0.03 |  |
|  | Divorced | 0.21 | (0.15, 0.28) | 0.03 |  |
|  | Widowed | 0.38 | (0.31, 0.46) | 0.04 |  |
|  | Never | 0.14 | (0.11, 0.16) | 0.01 |  |
|  | Domestic Partner | 0.12 | (0.09, 0.14) | 0.01 |  |
| Employment | Employed for an employer | 0.11 | (0.09, 0.12) | 0.01 | < .001 |
|  | Self-employed | 0.16 | (0.13, 0.19) | 0.01 |  |
|  | Retired | 0.33 | (0.26, 0.40) | 0.04 |  |
|  | Student | 0.10 | (0.04, 0.15) | 0.03 |  |
|  | Homemaker | 0.24 | (0.21, 0.27) | 0.02 |  |
|  | Unemployed and looking for a job | 0.17 | (0.13, 0.21) | 0.02 |  |
|  | None of these/Other | 0.32 | (0.22, 0.42) | 0.05 |  |
| Religious service attendance | At least 1/week | 0.24 | (0.20, 0.29) | 0.02 | < .001 |
|  | 1/week | 0.22 | (0.19, 0.26) | 0.02 |  |
|  | 1-3/month | 0.22 | (0.17, 0.26) | 0.02 |  |
|  | A few times a year | 0.14 | (0.12, 0.16) | 0.01 |  |
|  | Never | 0.13 | (0.10, 0.15) | 0.01 |  |
| Education | Up to 8 years | 0.29 | (0.26, 0.32) | 0.02 | < .001 |
|  | 9-15 years | 0.15 | (0.14, 0.17) | 0.01 |  |
|  | 16+ years | 0.12 | (0.09, 0.14) | 0.01 |  |
| Immigration status | Born in this country | 0.17 | (0.16, 0.19) | 0.01 | 0.923 |
|  | Born in another country | 0.18 | (0.08, 0.27) | 0.05 |  |
| Religious affiliation | Christianity | 0.18 | (0.17, 0.20) | 0.01 |  |
|  | Islam | 0.00 | * | * |  |
|  | Hinduism | 0.25 | * | * |  |
|  | Buddhism | 0.66 | * | * |  |
|  | Judaism | 0.20 | * | * |  |
|  | Baha'i | 0.00 | * | * |  |
|  | Jainism | 1.00 | * | * |  |
|  | Shinto | 0.00 | * | * |  |
|  | Taoism | 0.94 | * | * |  |
|  | Confucianism | 0.00 | * | * |  |
|  | Primal, Animist, or Folk religion | 0.04 | (0.00, 0.11) | 0.03 |  |
|  | Some other religion | 0.15 | (0.02, 0.28) | 0.06 |  |
|  | No religion/Atheist/Agnostic | 0.12 | (0.09, 0.15) | 0.01 |  |
| Race/ethnicity | Black | 0.21 | (0.12, 0.30) | 0.05 | < .001 |
|  | Indigenous | 0.19 | (0.15, 0.23) | 0.02 |  |
|  | White | 0.19 | (0.16, 0.21) | 0.01 |  |
|  | Mestizo | 0.15 | (0.14, 0.17) | 0.01 |  |
|  | Mulatto | 0.15 | (0.04, 0.26) | 0.05 |  |
|  | Other | 0.29 | (0.23, 0.36) | 0.03 |  |

***Table S13a. Nationally representative descriptive statistics for Nigeria***

| **Characteristic** | **N = 6,827**^1^ |
| --- | --- |
| **Age group** |  |
| 18-24 | 1,533 (22%) |
| 25-29 | 1,193 (17%) |
| 30-39 | 1,943 (28%) |
| 40-49 | 1,059 (16%) |
| 50-59 | 619 (9.1%) |
| 60-69 | 296 (4.3%) |
| 70-79 | 133 (2.0%) |
| 80 or older | 50 (0.7%) |
| (Missing) | 0 (0%) |
| **Gender** |  |
| Male | 3,371 (49%) |
| Female | 3,456 (51%) |
| Other | 0 (<0.1%) |
| (Missing) | 0 (0%) |
| **Marital status** |  |
| Married | 4,065 (60%) |
| Separated | 117 (1.7%) |
| Divorced | 71 (1.0%) |
| Widowed | 231 (3.4%) |
| Never | 2,289 (34%) |
| Domestic Partner | 12 (0.2%) |
| (Missing) | 42 (0.6%) |
| **Employment** |  |
| Employed for an employer | 699 (10%) |
| Self-employed | 3,898 (57%) |
| Retired | 178 (2.6%) |
| Student | 650 (9.5%) |
| Homemaker | 499 (7.3%) |
| Unemployed and looking for a job | 684 (10%) |
| None of these/Other | 211 (3.1%) |
| (Missing) | 8 (0.1%) |
| **Religious service attendance** |  |
| At least 1/week | 4,049 (59%) |
| 1/week | 1,895 (28%) |
| 1-3/month | 531 (7.8%) |
| A few times a year | 254 (3.7%) |
| Never | 77 (1.1%) |
| (Missing) | 20 (0.3%) |
| **Education** |  |
| Up to 8 years | 2,575 (38%) |
| 9-15 years | 4,120 (60%) |
| 16+ years | 130 (1.9%) |
| (Missing) | 2 (<0.1%) |
| **Immigration** |  |
| Born in this country | 6,779 (99%) |
| Born in another country | 47 (0.7%) |
| (Missing) | 1 (<0.1%) |
| **Religious affiliation** |  |
| Christianity | 3,476 (51%) |
| Islam | 3,302 (48%) |
| Hinduism | 0 (0%) |
| Buddhism | 0 (0%) |
| Judaism | 0 (0%) |
| Sikhism | 0 (0%) |
| Baha'i | 0 (0%) |
| Jainism | 0 (0%) |
| Shinto | 1 (<0.1%) |
| Taoism | 0 (0%) |
| Confucianism | 0 (<0.1%) |
| Primal, Animist, or Folk religion | 24 (0.3%) |
| Spiritism | 0 (0%) |
| Umbanda, Candomble, and other African-derived religions | 0 (0%) |
| Chinese folk/traditional religion | 0 (0%) |
| Some other religion | 1 (<0.1%) |
| No religion/Atheist/Agnostic | 15 (0.2%) |
| (Missing) | 9 (0.1%) |
| **Race/Ethnicity** |  |
| Edo | 116 (1.7%) |
| Efik | 48 (0.7%) |
| Fulani | 266 (3.9%) |
| Hausa | 2,342 (34%) |
| Ibibio | 180 (2.6%) |
| Idoma | 61 (0.9%) |
| Igala | 77 (1.1%) |
| Igbo (Ibo) | 1,111 (16%) |
| Ijaw | 110 (1.6%) |
| Kanuri | 31 (0.5%) |
| Other | 1,014 (15%) |
| Tiv | 198 (2.9%) |
| Urhobo | 38 (0.6%) |
| Yoruba | 1,230 (18%) |
| (Missing) | 4 (<0.1%) |
| ^1^n (%) | |

***Table S13b. Proportions by demographic category for Nigeria***

| Variable | Category | Mean | 95% CI | SE | Global p-value |
| --- | --- | --- | --- | --- | --- |
| Age group | 18-24 | 0.09 | (0.07, 0.11) | 0.01 | < .001 |
|  | 25-29 | 0.10 | (0.07, 0.12) | 0.01 |  |
|  | 30-39 | 0.09 | (0.07, 0.11) | 0.01 |  |
|  | 40-49 | 0.17 | (0.13, 0.20) | 0.02 |  |
|  | 50-59 | 0.24 | (0.18, 0.31) | 0.03 |  |
|  | 60-69 | 0.35 | (0.23, 0.46) | 0.06 |  |
|  | 70-79 | 0.36 | (0.22, 0.50) | 0.07 |  |
|  | 80 or older | 0.77 | (0.50, 1.00) | 0.13 |  |
| Gender | Male | 0.14 | (0.12, 0.16) | 0.01 | < .001 |
|  | Female | 0.14 | (0.12, 0.16) | 0.01 |  |
|  | Other | 0.00 | * | * |  |
| Marital status | Married | 0.14 | (0.12, 0.16) | 0.01 | < .001 |
|  | Separated | 0.27 | (0.16, 0.39) | 0.06 |  |
|  | Divorced | 0.23 | (0.08, 0.39) | 0.08 |  |
|  | Widowed | 0.43 | (0.30, 0.57) | 0.07 |  |
|  | Never | 0.10 | (0.09, 0.12) | 0.01 |  |
|  | Domestic Partner | 0.00 | * | * |  |
| Employment | Employed for an employer | 0.13 | (0.10, 0.16) | 0.02 | < .001 |
|  | Self-employed | 0.12 | (0.10, 0.14) | 0.01 |  |
|  | Retired | 0.43 | (0.29, 0.57) | 0.07 |  |
|  | Student | 0.09 | (0.07, 0.12) | 0.01 |  |
|  | Homemaker | 0.17 | (0.11, 0.23) | 0.03 |  |
|  | Unemployed and looking for a job | 0.15 | (0.11, 0.19) | 0.02 |  |
|  | None of these/Other | 0.26 | (0.11, 0.42) | 0.08 |  |
| Religious service attendance | At least 1/week | 0.13 | (0.11, 0.15) | 0.01 | 0.096 |
|  | 1/week | 0.14 | (0.11, 0.16) | 0.01 |  |
|  | 1-3/month | 0.17 | (0.12, 0.22) | 0.03 |  |
|  | A few times a year | 0.19 | (0.12, 0.27) | 0.04 |  |
|  | Never | 0.26 | (0.11, 0.41) | 0.08 |  |
| Education | Up to 8 years | 0.17 | (0.13, 0.20) | 0.02 | < .001 |
|  | 9-15 years | 0.12 | (0.11, 0.14) | 0.01 |  |
|  | 16+ years | 0.08 | (0.04, 0.11) | 0.02 |  |
| Immigration status | Born in this country | 0.14 | (0.12, 0.15) | 0.01 | 0.070 |
|  | Born in another country | 0.33 | (0.12, 0.54) | 0.10 |  |
| Religious affiliation | Christianity | 0.18 | (0.15, 0.20) | 0.01 | < .001 |
|  | Islam | 0.10 | (0.08, 0.12) | 0.01 |  |
|  | Shinto | 0.00 | * | * |  |
|  | Confucianism | 0.00 | * | * |  |
|  | Primal, Animist, or Folk religion | 0.09 | (0.00, 0.28) | 0.09 |  |
|  | Some other religion | 0.00 | * | * |  |
|  | No religion/Atheist/Agnostic | 0.04 | (0.00, 0.14) | 0.04 |  |
| Race/ethnicity | Hausa | 0.09 | (0.07, 0.11) | 0.01 | < .001 |
|  | Yoruba | 0.16 | (0.11, 0.21) | 0.03 |  |
|  | Igbo (Ibo) | 0.16 | (0.13, 0.20) | 0.02 |  |
|  | Edo | 0.12 | (0.06, 0.18) | 0.03 |  |
|  | Urhobo | 0.02 | (0.00, 0.06) | 0.02 |  |
|  | Fulani | 0.12 | (0.04, 0.20) | 0.04 |  |
|  | Kanuri | 0.18 | (0.01, 0.35) | 0.08 |  |
|  | Tiv | 0.24 | (0.18, 0.29) | 0.03 |  |
|  | Efik | 0.14 | (0.05, 0.24) | 0.05 |  |
|  | Ijaw | 0.15 | (0.03, 0.28) | 0.06 |  |
|  | Igala | 0.08 | (0.01, 0.15) | 0.04 |  |
|  | Ibibio | 0.36 | (0.25, 0.47) | 0.06 |  |
|  | Idoma | 0.05 | (0.00, 0.10) | 0.03 |  |
|  | Other | 0.15 | (0.11, 0.18) | 0.02 |  |

***Table S14a. Nationally representative descriptive statistics for Philippines***

| **Characteristic** | **N = 5,292**^1^ |
| --- | --- |
| **Age group** |  |
| 18-24 | 1,073 (20%) |
| 25-29 | 695 (13%) |
| 30-39 | 1,160 (22%) |
| 40-49 | 972 (18%) |
| 50-59 | 732 (14%) |
| 60-69 | 495 (9.4%) |
| 70-79 | 143 (2.7%) |
| 80 or older | 23 (0.4%) |
| (Missing) | 0 (0%) |
| **Gender** |  |
| Male | 2,625 (50%) |
| Female | 2,643 (50%) |
| Other | 13 (0.2%) |
| (Missing) | 11 (0.2%) |
| **Marital status** |  |
| Married | 2,385 (45%) |
| Separated | 249 (4.7%) |
| Divorced | 9 (0.2%) |
| Widowed | 274 (5.2%) |
| Never | 1,206 (23%) |
| Domestic Partner | 1,152 (22%) |
| (Missing) | 16 (0.3%) |
| **Employment** |  |
| Employed for an employer | 1,350 (26%) |
| Self-employed | 1,379 (26%) |
| Retired | 158 (3.0%) |
| Student | 585 (11%) |
| Homemaker | 1,049 (20%) |
| Unemployed and looking for a job | 658 (12%) |
| None of these/Other | 113 (2.1%) |
| (Missing) | 0 (0%) |
| **Religious service attendance** |  |
| At least 1/week | 844 (16%) |
| 1/week | 1,929 (36%) |
| 1-3/month | 1,374 (26%) |
| A few times a year | 929 (18%) |
| Never | 210 (4.0%) |
| (Missing) | 6 (0.1%) |
| **Education** |  |
| Up to 8 years | 1,188 (22%) |
| 9-15 years | 3,722 (70%) |
| 16+ years | 381 (7.2%) |
| (Missing) | 1 (<0.1%) |
| **Immigration** |  |
| Born in this country | 5,284 (100%) |
| Born in another country | 8 (0.1%) |
| (Missing) | 0 (0%) |
| **Religious affiliation** |  |
| Christianity | 4,914 (93%) |
| Islam | 297 (5.6%) |
| Hinduism | 0 (0%) |
| Buddhism | 4 (<0.1%) |
| Judaism | 4 (<0.1%) |
| Sikhism | 0 (0%) |
| Baha'i | 1 (<0.1%) |
| Jainism | 0 (0%) |
| Shinto | 0 (0%) |
| Taoism | 0 (0%) |
| Confucianism | 0 (0%) |
| Primal, Animist, or Folk religion | 5 (<0.1%) |
| Spiritism | 0 (0%) |
| Umbanda, Candomble, and other African-derived religions | 0 (0%) |
| Chinese folk/traditional religion | 0 (0%) |
| Some other religion | 35 (0.7%) |
| No religion/Atheist/Agnostic | 23 (0.4%) |
| (Missing) | 9 (0.2%) |
| **Race/Ethnicity** |  |
| Aeta | 1 (<0.1%) |
| Badjao | 2 (<0.1%) |
| Bicolano/Bikolano | 300 (5.7%) |
| Cebuano | 656 (12%) |
| Chinese-Filipino | 3 (<0.1%) |
| Igorot | 42 (0.8%) |
| Ilocano/Ilokano | 429 (8.1%) |
| Ilonggo/Hiligaynon | 428 (8.1%) |
| Kapampangan | 107 (2.0%) |
| Maguindanaoan | 84 (1.6%) |
| Mangyan | 2 (<0.1%) |
| Maranao | 39 (0.7%) |
| Masbateno | 54 (1.0%) |
| Other | 244 (4.6%) |
| Pangasinense | 107 (2.0%) |
| Tagalog | 1,691 (32%) |
| Tausug | 94 (1.8%) |
| Visayan/Bisaya | 739 (14%) |
| Waray | 216 (4.1%) |
| Zamboangueno | 51 (1.0%) |
| (Missing) | 3 (<0.1%) |
| ^1^n (%) | |

***Table S14b. Proportions by demographic category for Philippines***

| Variable | Category | Mean | 95% CI | SE | Global p-value |
| --- | --- | --- | --- | --- | --- |
| Age group | 18-24 | 0.26 | (0.22, 0.30) | 0.02 | < .001 |
|  | 25-29 | 0.24 | (0.20, 0.28) | 0.02 |  |
|  | 30-39 | 0.30 | (0.27, 0.33) | 0.02 |  |
|  | 40-49 | 0.37 | (0.33, 0.41) | 0.02 |  |
|  | 50-59 | 0.43 | (0.39, 0.47) | 0.02 |  |
|  | 60-69 | 0.52 | (0.46, 0.58) | 0.03 |  |
|  | 70-79 | 0.54 | (0.44, 0.65) | 0.06 |  |
|  | 80 or older | 0.60 | (0.31, 0.90) | 0.13 |  |
| Gender | Male | 0.36 | (0.33, 0.38) | 0.01 | 0.214 |
|  | Female | 0.33 | (0.31, 0.35) | 0.01 |  |
|  | Other | 0.50 | (0.19, 0.81) | 0.13 |  |
| Marital status | Married | 0.38 | (0.35, 0.40) | 0.01 | < .001 |
|  | Separated | 0.43 | (0.35, 0.50) | 0.04 |  |
|  | Divorced | 0.48 | (0.00, 1.00) | 0.16 |  |
|  | Widowed | 0.54 | (0.48, 0.60) | 0.03 |  |
|  | Never | 0.26 | (0.22, 0.29) | 0.02 |  |
|  | Domestic Partner | 0.31 | (0.27, 0.34) | 0.02 |  |
| Employment | Employed for an employer | 0.32 | (0.29, 0.35) | 0.02 | < .001 |
|  | Self-employed | 0.35 | (0.32, 0.38) | 0.02 |  |
|  | Retired | 0.67 | (0.56, 0.78) | 0.06 |  |
|  | Student | 0.24 | (0.19, 0.28) | 0.02 |  |
|  | Homemaker | 0.37 | (0.34, 0.40) | 0.01 |  |
|  | Unemployed and looking for a job | 0.36 | (0.32, 0.41) | 0.02 |  |
|  | None of these/Other | 0.35 | (0.24, 0.46) | 0.06 |  |
| Religious service attendance | At least 1/week | 0.36 | (0.32, 0.40) | 0.02 | 0.442 |
|  | 1/week | 0.33 | (0.31, 0.36) | 0.01 |  |
|  | 1-3/month | 0.34 | (0.31, 0.37) | 0.02 |  |
|  | A few times a year | 0.35 | (0.31, 0.39) | 0.02 |  |
|  | Never | 0.41 | (0.31, 0.50) | 0.05 |  |
| Education | Up to 8 years | 0.43 | (0.39, 0.46) | 0.02 | < .001 |
|  | 9-15 years | 0.32 | (0.30, 0.35) | 0.01 |  |
|  | 16+ years | 0.28 | (0.22, 0.35) | 0.03 |  |
| Immigration status | Born in this country | 0.34 | (0.33, 0.36) | 0.01 | 0.320 |
|  | Born in another country | 0.18 | (0.00, 0.65) | 0.16 |  |
| Religious affiliation | Christianity | 0.34 | (0.32, 0.36) | 0.01 | 0.002 |
|  | Islam | 0.39 | (0.33, 0.44) | 0.03 |  |
|  | Buddhism | 0.00 | * | * |  |
|  | Judaism | 0.17 | * | * |  |
|  | Baha'i | 0.00 | * | * |  |
|  | Primal, Animist, or Folk religion | 0.86 | (0.57, 1.00) | 0.15 |  |
|  | Some other religion | 0.47 | (0.31, 0.63) | 0.08 |  |
|  | No religion/Atheist/Agnostic | 0.36 | (0.03, 0.69) | 0.15 |  |
| Race/ethnicity | Tagalog | 0.29 | (0.26, 0.32) | 0.01 | < .001 |
|  | Cebuano | 0.43 | (0.38, 0.48) | 0.03 |  |
|  | Ilocano/Ilokano | 0.33 | (0.29, 0.37) | 0.02 |  |
|  | Visayan/Bisaya | 0.38 | (0.33, 0.42) | 0.02 |  |
|  | Ilonggo/Hiligaynon | 0.38 | (0.33, 0.43) | 0.02 |  |
|  | Bicolano/Bikolano | 0.35 | (0.28, 0.41) | 0.03 |  |
|  | Waray | 0.35 | (0.28, 0.42) | 0.04 |  |
|  | Tausug | 0.42 | (0.36, 0.47) | 0.03 |  |
|  | Maranao | 0.35 | (0.16, 0.55) | 0.09 |  |
|  | Maguindanaoan | 0.29 | (0.20, 0.39) | 0.05 |  |
|  | Chinese-Filipino | 0.19 | * | * |  |
|  | Kapampangan | 0.39 | (0.27, 0.51) | 0.06 |  |
|  | Pangasinense | 0.23 | (0.12, 0.34) | 0.05 |  |
|  | Zamboangueno | 0.30 | (0.11, 0.49) | 0.09 |  |
|  | Masbateno | 0.25 | (0.16, 0.34) | 0.04 |  |
|  | Aeta | 0.00 | * | * |  |
|  | Igorot | 0.29 | (0.10, 0.47) | 0.09 |  |
|  | Mangyan | 0.00 | * | * |  |
|  | Badjao | 0.19 | * | * |  |
|  | Other | 0.39 | (0.31, 0.48) | 0.04 |  |

***Table S15a. Nationally representative descriptive statistics for Poland***

| **Characteristic** | **N = 10,389**^1^ |
| --- | --- |
| **Age group** |  |
| 18-24 | 955 (9.2%) |
| 25-29 | 761 (7.3%) |
| 30-39 | 2,159 (21%) |
| 40-49 | 1,956 (19%) |
| 50-59 | 1,670 (16%) |
| 60-69 | 1,909 (18%) |
| 70-79 | 833 (8.0%) |
| 80 or older | 145 (1.4%) |
| (Missing) | 1 (<0.1%) |
| **Gender** |  |
| Male | 4,974 (48%) |
| Female | 5,387 (52%) |
| Other | 3 (<0.1%) |
| (Missing) | 26 (0.2%) |
| **Marital status** |  |
| Married | 6,065 (58%) |
| Separated | 111 (1.1%) |
| Divorced | 529 (5.1%) |
| Widowed | 990 (9.5%) |
| Never | 1,811 (17%) |
| Domestic Partner | 504 (4.8%) |
| (Missing) | 379 (3.6%) |
| **Employment** |  |
| Employed for an employer | 5,837 (56%) |
| Self-employed | 686 (6.6%) |
| Retired | 2,434 (23%) |
| Student | 515 (5.0%) |
| Homemaker | 338 (3.3%) |
| Unemployed and looking for a job | 284 (2.7%) |
| None of these/Other | 169 (1.6%) |
| (Missing) | 126 (1.2%) |
| **Religious service attendance** |  |
| At least 1/week | 305 (2.9%) |
| 1/week | 3,263 (31%) |
| 1-3/month | 2,081 (20%) |
| A few times a year | 3,064 (29%) |
| Never | 1,597 (15%) |
| (Missing) | 78 (0.8%) |
| **Education** |  |
| Up to 8 years | 1,238 (12%) |
| 9-15 years | 6,130 (59%) |
| 16+ years | 3,020 (29%) |
| (Missing) | 1 (<0.1%) |
| **Immigration** |  |
| Born in this country | 10,258 (99%) |
| Born in another country | 108 (1.0%) |
| (Missing) | 23 (0.2%) |
| **Religious affiliation** |  |
| Christianity | 9,378 (90%) |
| Islam | 2 (<0.1%) |
| Hinduism | 0 (0%) |
| Buddhism | 2 (<0.1%) |
| Judaism | 0 (0%) |
| Sikhism | 1 (<0.1%) |
| Baha'i | 0 (0%) |
| Jainism | 3 (<0.1%) |
| Shinto | 1 (<0.1%) |
| Taoism | 0 (0%) |
| Confucianism | 0 (0%) |
| Primal, Animist, or Folk religion | 11 (0.1%) |
| Spiritism | 0 (0%) |
| Umbanda, Candomble, and other African-derived religions | 0 (0%) |
| Chinese folk/traditional religion | 0 (0%) |
| Some other religion | 0 (0%) |
| No religion/Atheist/Agnostic | 942 (9.1%) |
| (Missing) | 50 (0.5%) |
| **Race/Ethnicity** |  |
| Belarussian | 2 (<0.1%) |
| German | 4 (<0.1%) |
| Kashubians | 3 (<0.1%) |
| Other | 4 (<0.1%) |
| Polish | 10,309 (99%) |
| Silesia | 14 (0.1%) |
| Ukrainian | 38 (0.4%) |
| (Missing) | 14 (0.1%) |
| ^1^n (%) | |

***Table S15b. Proportions by demographic category for Poland***

| Variable | Category | Mean | 95% CI | SE | Global p-value |
| --- | --- | --- | --- | --- | --- |
| Age group | 18-24 | 0.03 | (0.02, 0.04) | 0.01 | < .001 |
|  | 25-29 | 0.04 | (0.03, 0.05) | 0.01 |  |
|  | 30-39 | 0.05 | (0.03, 0.06) | 0.01 |  |
|  | 40-49 | 0.06 | (0.05, 0.08) | 0.01 |  |
|  | 50-59 | 0.13 | (0.10, 0.16) | 0.02 |  |
|  | 60-69 | 0.27 | (0.23, 0.31) | 0.02 |  |
|  | 70-79 | 0.37 | (0.29, 0.45) | 0.04 |  |
|  | 80 or older | 0.39 | (0.22, 0.55) | 0.08 |  |
| Gender | Male | 0.13 | (0.11, 0.14) | 0.01 | < .001 |
|  | Female | 0.14 | (0.12, 0.16) | 0.01 |  |
|  | Other | 0.00 | * | * |  |
| Marital status | Married | 0.11 | (0.10, 0.13) | 0.01 | < .001 |
|  | Separated | 0.07 | (0.02, 0.12) | 0.02 |  |
|  | Divorced | 0.19 | (0.12, 0.25) | 0.03 |  |
|  | Widowed | 0.38 | (0.30, 0.45) | 0.04 |  |
|  | Never | 0.08 | (0.06, 0.10) | 0.01 |  |
|  | Domestic Partner | 0.07 | (0.05, 0.09) | 0.01 |  |
| Employment | Employed for an employer | 0.07 | (0.06, 0.08) | 0.01 | < .001 |
|  | Self-employed | 0.07 | (0.04, 0.09) | 0.01 |  |
|  | Retired | 0.31 | (0.27, 0.36) | 0.02 |  |
|  | Student | 0.01 | (0.00, 0.02) | 0.00 |  |
|  | Homemaker | 0.13 | (0.06, 0.21) | 0.04 |  |
|  | Unemployed and looking for a job | 0.15 | (0.08, 0.23) | 0.04 |  |
|  | None of these/Other | 0.39 | (0.24, 0.53) | 0.07 |  |
| Religious service attendance | At least 1/week | 0.37 | (0.24, 0.50) | 0.07 | < .001 |
|  | 1/week | 0.16 | (0.13, 0.18) | 0.01 |  |
|  | 1-3/month | 0.10 | (0.08, 0.13) | 0.01 |  |
|  | A few times a year | 0.11 | (0.09, 0.13) | 0.01 |  |
|  | Never | 0.13 | (0.10, 0.16) | 0.02 |  |
| Education | Up to 8 years | 0.24 | (0.16, 0.33) | 0.05 | 0.019 |
|  | 9-15 years | 0.12 | (0.11, 0.13) | 0.01 |  |
|  | 16+ years | 0.12 | (0.10, 0.14) | 0.01 |  |
| Immigration status | Born in this country | 0.13 | (0.12, 0.15) | 0.01 | 0.026 |
|  | Born in another country | 0.31 | (0.15, 0.47) | 0.08 |  |
| Religious affiliation | Christianity | 0.14 | (0.12, 0.15) | 0.01 | 0.912 |
|  | Islam | 0.00 | * | * |  |
|  | Buddhism | 0.00 | * | * |  |
|  | Sikhism | 0.00 | * | * |  |
|  | Jainism | 0.00 | * | * |  |
|  | Shinto | 0.00 | * | * |  |
|  | Primal, Animist, or Folk religion | 0.13 | (0.00, 0.64) | 0.08 |  |
|  | No religion/Atheist/Agnostic | 0.12 | (0.08, 0.15) | 0.02 |  |
| Race/ethnicity | Polish | 0.13 | (0.12, 0.15) | 0.01 | 0.709 |
|  | German | 0.21 | * | * |  |
|  | Belarussian | 0.00 | * | * |  |
|  | Ukrainian | 0.04 | (0.00, 0.14) | 0.05 |  |
|  | Silesia | 0.04 | (0.00, 0.16) | 0.05 |  |
|  | Kashubians | 0.00 | * | * |  |
|  | Other | 0.00 | * | * |  |

***Table S16a. Nationally representative descriptive statistics for South Africa***

| **Characteristic** | **N = 2,651**^1^ |
| --- | --- |
| **Age group** |  |
| 18-24 | 461 (17%) |
| 25-29 | 364 (14%) |
| 30-39 | 655 (25%) |
| 40-49 | 522 (20%) |
| 50-59 | 309 (12%) |
| 60-69 | 195 (7.4%) |
| 70-79 | 120 (4.5%) |
| 80 or older | 17 (0.6%) |
| (Missing) | 9 (0.3%) |
| **Gender** |  |
| Male | 1,288 (49%) |
| Female | 1,356 (51%) |
| Other | 2 (<0.1%) |
| (Missing) | 4 (0.2%) |
| **Marital status** |  |
| Married | 539 (20%) |
| Separated | 76 (2.9%) |
| Divorced | 51 (1.9%) |
| Widowed | 133 (5.0%) |
| Never | 1,561 (59%) |
| Domestic Partner | 264 (10.0%) |
| (Missing) | 28 (1.0%) |
| **Employment** |  |
| Employed for an employer | 569 (21%) |
| Self-employed | 412 (16%) |
| Retired | 243 (9.2%) |
| Student | 204 (7.7%) |
| Homemaker | 137 (5.2%) |
| Unemployed and looking for a job | 1,008 (38%) |
| None of these/Other | 74 (2.8%) |
| (Missing) | 3 (0.1%) |
| **Religious service attendance** |  |
| At least 1/week | 414 (16%) |
| 1/week | 891 (34%) |
| 1-3/month | 574 (22%) |
| A few times a year | 431 (16%) |
| Never | 334 (13%) |
| (Missing) | 7 (0.3%) |
| **Education** |  |
| Up to 8 years | 668 (25%) |
| 9-15 years | 1,796 (68%) |
| 16+ years | 183 (6.9%) |
| (Missing) | 4 (0.2%) |
| **Immigration** |  |
| Born in this country | 2,511 (95%) |
| Born in another country | 139 (5.2%) |
| (Missing) | 1 (<0.1%) |
| **Religious affiliation** |  |
| Christianity | 2,163 (82%) |
| Islam | 62 (2.3%) |
| Hinduism | 1 (<0.1%) |
| Buddhism | 12 (0.5%) |
| Judaism | 0 (0%) |
| Sikhism | 0 (0%) |
| Baha'i | 0 (0%) |
| Jainism | 2 (<0.1%) |
| Shinto | 2 (<0.1%) |
| Taoism | 1 (<0.1%) |
| Confucianism | 0 (0%) |
| Primal, Animist, or Folk religion | 127 (4.8%) |
| Spiritism | 0 (0%) |
| Umbanda, Candomble, and other African-derived religions | 0 (0%) |
| Chinese folk/traditional religion | 0 (0%) |
| Some other religion | 5 (0.2%) |
| No religion/Atheist/Agnostic | 253 (9.6%) |
| (Missing) | 23 (0.9%) |
| **Race/Ethnicity** |  |
| Asian/Indian | 6 (0.2%) |
| Black | 2,381 (90%) |
| Colored | 252 (9.5%) |
| Other | 1 (<0.1%) |
| White | 8 (0.3%) |
| (Missing) | 3 (0.1%) |
| ^1^n (%) | |

***Table S16b. Proportions by demographic category for South Africa***

| Variable | Category | Mean | 95% CI | SE | Global p-value |
| --- | --- | --- | --- | --- | --- |
| Age group | 18-24 | 0.14 | (0.10, 0.19) | 0.02 | < .001 |
|  | 25-29 | 0.15 | (0.11, 0.18) | 0.02 |  |
|  | 30-39 | 0.17 | (0.13, 0.20) | 0.02 |  |
|  | 40-49 | 0.23 | (0.18, 0.28) | 0.02 |  |
|  | 50-59 | 0.29 | (0.22, 0.36) | 0.04 |  |
|  | 60-69 | 0.46 | (0.34, 0.57) | 0.06 |  |
|  | 70-79 | 0.38 | (0.22, 0.55) | 0.08 |  |
|  | 80 or older | 0.49 | (0.00, 1.00) | 0.22 |  |
| Gender | Male | 0.19 | (0.16, 0.22) | 0.02 | 0.034 |
|  | Female | 0.25 | (0.21, 0.28) | 0.02 |  |
|  | Other | 0.42 | * | * |  |
| Marital status | Married | 0.27 | (0.21, 0.32) | 0.03 | < .001 |
|  | Separated | 0.33 | (0.19, 0.46) | 0.07 |  |
|  | Divorced | 0.22 | (0.03, 0.40) | 0.09 |  |
|  | Widowed | 0.42 | (0.28, 0.56) | 0.07 |  |
|  | Never | 0.18 | (0.15, 0.21) | 0.01 |  |
|  | Domestic Partner | 0.23 | (0.16, 0.29) | 0.03 |  |
| Employment | Employed for an employer | 0.18 | (0.14, 0.23) | 0.02 | < .001 |
|  | Self-employed | 0.16 | (0.12, 0.21) | 0.02 |  |
|  | Retired | 0.47 | (0.36, 0.58) | 0.06 |  |
|  | Student | 0.16 | (0.09, 0.24) | 0.04 |  |
|  | Homemaker | 0.38 | (0.28, 0.47) | 0.05 |  |
|  | Unemployed and looking for a job | 0.19 | (0.15, 0.22) | 0.02 |  |
|  | None of these/Other | 0.31 | (0.14, 0.48) | 0.09 |  |
| Religious service attendance | At least 1/week | 0.29 | (0.23, 0.36) | 0.03 | 0.007 |
|  | 1/week | 0.20 | (0.16, 0.23) | 0.02 |  |
|  | 1-3/month | 0.25 | (0.20, 0.30) | 0.02 |  |
|  | A few times a year | 0.17 | (0.12, 0.22) | 0.02 |  |
|  | Never | 0.20 | (0.14, 0.26) | 0.03 |  |
| Education | Up to 8 years | 0.33 | (0.27, 0.40) | 0.03 | < .001 |
|  | 9-15 years | 0.19 | (0.16, 0.21) | 0.01 |  |
|  | 16+ years | 0.13 | (0.08, 0.19) | 0.03 |  |
| Immigration status | Born in this country | 0.22 | (0.20, 0.25) | 0.01 | 0.310 |
|  | Born in another country | 0.17 | (0.08, 0.26) | 0.05 |  |
| Religious affiliation | Christianity | 0.23 | (0.20, 0.25) | 0.01 | < .001 |
|  | Islam | 0.35 | (0.21, 0.49) | 0.07 |  |
|  | Hinduism | 0.00 | * | * |  |
|  | Buddhism | 0.10 | * | * |  |
|  | Jainism | 0.00 | * | * |  |
|  | Shinto | 0.00 | * | * |  |
|  | Taoism | 0.00 | * | * |  |
|  | Primal, Animist, or Folk religion | 0.14 | (0.06, 0.23) | 0.04 |  |
|  | Some other religion | 0.00 | * | * |  |
|  | No religion/Atheist/Agnostic | 0.17 | (0.09, 0.24) | 0.04 |  |
| Race/ethnicity | Black | 0.22 | (0.19, 0.24) | 0.01 | 0.392 |
|  | White | 0.33 | (0.00, 1.00) | 0.14 |  |
|  | Asian/Indian | 0.00 | * | * |  |
|  | Colored | 0.25 | (0.16, 0.35) | 0.05 |  |
|  | Other | 1.00 | * | * |  |

***Table S17a. Nationally representative descriptive statistics for Spain***

| **Characteristic** | **N = 6,290**^1^ |
| --- | --- |
| **Age group** |  |
| 18-24 | 594 (9.4%) |
| 25-29 | 450 (7.2%) |
| 30-39 | 1,111 (18%) |
| 40-49 | 1,396 (22%) |
| 50-59 | 1,252 (20%) |
| 60-69 | 977 (16%) |
| 70-79 | 467 (7.4%) |
| 80 or older | 43 (0.7%) |
| (Missing) | 0 (0%) |
| **Gender** |  |
| Male | 3,142 (50%) |
| Female | 3,119 (50%) |
| Other | 6 (0.1%) |
| (Missing) | 22 (0.4%) |
| **Marital status** |  |
| Married | 2,947 (47%) |
| Separated | 237 (3.8%) |
| Divorced | 518 (8.2%) |
| Widowed | 189 (3.0%) |
| Never | 1,742 (28%) |
| Domestic Partner | 589 (9.4%) |
| (Missing) | 67 (1.1%) |
| **Employment** |  |
| Employed for an employer | 2,862 (45%) |
| Self-employed | 576 (9.2%) |
| Retired | 1,278 (20%) |
| Student | 448 (7.1%) |
| Homemaker | 345 (5.5%) |
| Unemployed and looking for a job | 646 (10%) |
| None of these/Other | 123 (2.0%) |
| (Missing) | 11 (0.2%) |
| **Religious service attendance** |  |
| At least 1/week | 317 (5.0%) |
| 1/week | 662 (11%) |
| 1-3/month | 437 (6.9%) |
| A few times a year | 1,972 (31%) |
| Never | 2,875 (46%) |
| (Missing) | 27 (0.4%) |
| **Education** |  |
| Up to 8 years | 802 (13%) |
| 9-15 years | 4,145 (66%) |
| 16+ years | 1,341 (21%) |
| (Missing) | 2 (<0.1%) |
| **Immigration** |  |
| Born in this country | 5,479 (87%) |
| Born in another country | 788 (13%) |
| (Missing) | 23 (0.4%) |
| **Religious affiliation** |  |
| Christianity | 4,074 (65%) |
| Islam | 135 (2.1%) |
| Hinduism | 7 (0.1%) |
| Buddhism | 36 (0.6%) |
| Judaism | 4 (<0.1%) |
| Sikhism | 3 (<0.1%) |
| Baha'i | 2 (<0.1%) |
| Jainism | 1 (<0.1%) |
| Shinto | 0 (0%) |
| Taoism | 5 (<0.1%) |
| Confucianism | 3 (<0.1%) |
| Primal, Animist, or Folk religion | 7 (0.1%) |
| Spiritism | 0 (0%) |
| Umbanda, Candomble, and other African-derived religions | 0 (0%) |
| Chinese folk/traditional religion | 0 (0%) |
| Some other religion | 27 (0.4%) |
| No religion/Atheist/Agnostic | 1,932 (31%) |
| (Missing) | 55 (0.9%) |
| ^1^n (%) | |

***Table S17b. Proportions by demographic category for Spain***

| Variable | Category | Mean | 95% CI | SE | Global p-value |
| --- | --- | --- | --- | --- | --- |
| Age group | 18-24 | 0.11 | (0.08, 0.14) | 0.02 | < .001 |
|  | 25-29 | 0.14 | (0.10, 0.17) | 0.02 |  |
|  | 30-39 | 0.13 | (0.11, 0.15) | 0.01 |  |
|  | 40-49 | 0.18 | (0.15, 0.20) | 0.01 |  |
|  | 50-59 | 0.23 | (0.20, 0.26) | 0.02 |  |
|  | 60-69 | 0.25 | (0.21, 0.30) | 0.02 |  |
|  | 70-79 | 0.35 | (0.27, 0.43) | 0.04 |  |
|  | 80 or older | 0.42 | (0.17, 0.66) | 0.12 |  |
| Gender | Male | 0.19 | (0.18, 0.21) | 0.01 | 0.672 |
|  | Female | 0.20 | (0.18, 0.22) | 0.01 |  |
|  | Other | 0.32 | (0.00, 1.00) | 0.14 |  |
| Marital status | Married | 0.22 | (0.19, 0.24) | 0.01 | < .001 |
|  | Separated | 0.31 | (0.23, 0.39) | 0.04 |  |
|  | Divorced | 0.19 | (0.14, 0.23) | 0.02 |  |
|  | Widowed | 0.36 | (0.24, 0.47) | 0.06 |  |
|  | Never | 0.15 | (0.13, 0.17) | 0.01 |  |
|  | Domestic Partner | 0.16 | (0.12, 0.20) | 0.02 |  |
| Employment | Employed for an employer | 0.13 | (0.12, 0.15) | 0.01 | < .001 |
|  | Self-employed | 0.16 | (0.12, 0.19) | 0.02 |  |
|  | Retired | 0.36 | (0.31, 0.40) | 0.02 |  |
|  | Student | 0.10 | (0.07, 0.13) | 0.02 |  |
|  | Homemaker | 0.27 | (0.20, 0.35) | 0.04 |  |
|  | Unemployed and looking for a job | 0.20 | (0.16, 0.23) | 0.02 |  |
|  | None of these/Other | 0.35 | (0.26, 0.44) | 0.05 |  |
| Religious service attendance | At least 1/week | 0.24 | (0.17, 0.30) | 0.03 | 0.017 |
|  | 1/week | 0.19 | (0.15, 0.23) | 0.02 |  |
|  | 1-3/month | 0.17 | (0.12, 0.22) | 0.03 |  |
|  | A few times a year | 0.23 | (0.20, 0.26) | 0.01 |  |
|  | Never | 0.18 | (0.16, 0.19) | 0.01 |  |
| Education | Up to 8 years | 0.29 | (0.24, 0.34) | 0.03 | < .001 |
|  | 9-15 years | 0.20 | (0.18, 0.22) | 0.01 |  |
|  | 16+ years | 0.13 | (0.11, 0.16) | 0.01 |  |
| Immigration status | Born in this country | 0.20 | (0.19, 0.22) | 0.01 | 0.007 |
|  | Born in another country | 0.16 | (0.13, 0.19) | 0.02 |  |
| Religious affiliation | Christianity | 0.20 | (0.19, 0.22) | 0.01 | < .001 |
|  | Islam | 0.22 | (0.13, 0.30) | 0.04 |  |
|  | Hinduism | 0.22 | * | * |  |
|  | Buddhism | 0.29 | (0.08, 0.50) | 0.10 |  |
|  | Judaism | 0.28 | * | * |  |
|  | Sikhism | 0.00 | * | * |  |
|  | Baha'i | 0.40 | * | * |  |
|  | Jainism | 0.00 | * | * |  |
|  | Taoism | 0.00 | * | * |  |
|  | Confucianism | 0.49 | * | * |  |
|  | Primal, Animist, or Folk religion | 0.34 | * | * |  |
|  | Some other religion | 0.44 | (0.15, 0.73) | 0.13 |  |
|  | No religion/Atheist/Agnostic | 0.18 | (0.15, 0.20) | 0.01 |  |

***Table S18a. Nationally representative descriptive statistics for Sweden***

| **Characteristic** | **N = 15,068**^1^ |
| --- | --- |
| **Age group** |  |
| 18-24 | 1,515 (10%) |
| 25-29 | 1,399 (9.3%) |
| 30-39 | 2,398 (16%) |
| 40-49 | 2,221 (15%) |
| 50-59 | 2,493 (17%) |
| 60-69 | 2,168 (14%) |
| 70-79 | 2,253 (15%) |
| 80 or older | 621 (4.1%) |
| (Missing) | 0 (0%) |
| **Gender** |  |
| Male | 7,536 (50%) |
| Female | 7,493 (50%) |
| Other | 27 (0.2%) |
| (Missing) | 12 (<0.1%) |
| **Marital status** |  |
| Married | 6,408 (43%) |
| Separated | 426 (2.8%) |
| Divorced | 801 (5.3%) |
| Widowed | 433 (2.9%) |
| Never | 3,854 (26%) |
| Domestic Partner | 3,073 (20%) |
| (Missing) | 72 (0.5%) |
| **Employment** |  |
| Employed for an employer | 7,907 (52%) |
| Self-employed | 1,243 (8.3%) |
| Retired | 3,832 (25%) |
| Student | 1,332 (8.8%) |
| Homemaker | 75 (0.5%) |
| Unemployed and looking for a job | 324 (2.2%) |
| None of these/Other | 337 (2.2%) |
| (Missing) | 18 (0.1%) |
| **Religious service attendance** |  |
| At least 1/week | 236 (1.6%) |
| 1/week | 434 (2.9%) |
| 1-3/month | 486 (3.2%) |
| A few times a year | 3,950 (26%) |
| Never | 9,918 (66%) |
| (Missing) | 45 (0.3%) |
| **Education** |  |
| Up to 8 years | 252 (1.7%) |
| 9-15 years | 10,790 (72%) |
| 16+ years | 4,026 (27%) |
| (Missing) | 0 (0%) |
| **Immigration** |  |
| Born in this country | 13,922 (92%) |
| Born in another country | 1,052 (7.0%) |
| (Missing) | 94 (0.6%) |
| **Religious affiliation** |  |
| Christianity | 8,346 (55%) |
| Islam | 470 (3.1%) |
| Hinduism | 22 (0.1%) |
| Buddhism | 110 (0.7%) |
| Judaism | 54 (0.4%) |
| Sikhism | 4 (<0.1%) |
| Baha'i | 6 (<0.1%) |
| Jainism | 0 (0%) |
| Shinto | 0 (<0.1%) |
| Taoism | 4 (<0.1%) |
| Confucianism | 0 (0%) |
| Primal, Animist, or Folk religion | 83 (0.5%) |
| Spiritism | 0 (0%) |
| Umbanda, Candomble, and other African-derived religions | 0 (0%) |
| Chinese folk/traditional religion | 0 (0%) |
| Some other religion | 198 (1.3%) |
| No religion/Atheist/Agnostic | 5,697 (38%) |
| (Missing) | 74 (0.5%) |
| ^1^n (%) | |

***Table S18b. Proportions by demographic category for Sweden***

| Variable | Category | Mean | 95% CI | SE | Global p-value |
| --- | --- | --- | --- | --- | --- |
| Age group | 18-24 | 0.19 | (0.16, 0.21) | 0.01 | < .001 |
|  | 25-29 | 0.19 | (0.16, 0.21) | 0.01 |  |
|  | 30-39 | 0.20 | (0.18, 0.22) | 0.01 |  |
|  | 40-49 | 0.23 | (0.21, 0.25) | 0.01 |  |
|  | 50-59 | 0.27 | (0.25, 0.30) | 0.01 |  |
|  | 60-69 | 0.34 | (0.32, 0.37) | 0.01 |  |
|  | 70-79 | 0.31 | (0.29, 0.34) | 0.01 |  |
|  | 80 or older | 0.34 | (0.30, 0.39) | 0.02 |  |
| Gender | Male | 0.21 | (0.20, 0.23) | 0.01 | < .001 |
|  | Female | 0.30 | (0.29, 0.31) | 0.01 |  |
|  | Other | 0.64 | (0.43, 0.85) | 0.10 |  |
| Marital status | Married | 0.25 | (0.24, 0.27) | 0.01 | < .001 |
|  | Separated | 0.28 | (0.23, 0.33) | 0.02 |  |
|  | Divorced | 0.37 | (0.33, 0.41) | 0.02 |  |
|  | Widowed | 0.33 | (0.28, 0.39) | 0.03 |  |
|  | Never | 0.26 | (0.25, 0.28) | 0.01 |  |
|  | Domestic Partner | 0.21 | (0.19, 0.23) | 0.01 |  |
| Employment | Employed for an employer | 0.20 | (0.18, 0.21) | 0.01 | < .001 |
|  | Self-employed | 0.20 | (0.17, 0.23) | 0.02 |  |
|  | Retired | 0.37 | (0.35, 0.39) | 0.01 |  |
|  | Student | 0.19 | (0.16, 0.21) | 0.01 |  |
|  | Homemaker | 0.30 | (0.17, 0.43) | 0.06 |  |
|  | Unemployed and looking for a job | 0.43 | (0.37, 0.48) | 0.03 |  |
|  | None of these/Other | 0.72 | (0.67, 0.78) | 0.03 |  |
| Religious service attendance | At least 1/week | 0.39 | (0.30, 0.47) | 0.04 | 0.014 |
|  | 1/week | 0.26 | (0.20, 0.32) | 0.03 |  |
|  | 1-3/month | 0.29 | (0.24, 0.35) | 0.03 |  |
|  | A few times a year | 0.24 | (0.23, 0.26) | 0.01 |  |
|  | Never | 0.26 | (0.25, 0.27) | 0.01 |  |
| Education | Up to 8 years | 0.41 | (0.33, 0.49) | 0.04 | < .001 |
|  | 9-15 years | 0.28 | (0.27, 0.29) | 0.01 |  |
|  | 16+ years | 0.20 | (0.18, 0.21) | 0.01 |  |
| Immigration status | Born in this country | 0.26 | (0.25, 0.27) | 0.00 | 0.766 |
|  | Born in another country | 0.26 | (0.23, 0.30) | 0.02 |  |
| Religious affiliation | Christianity | 0.27 | (0.25, 0.28) | 0.01 | < .001 |
|  | Islam | 0.20 | (0.14, 0.25) | 0.03 |  |
|  | Hinduism | 0.23 | (0.00, 0.57) | 0.14 |  |
|  | Buddhism | 0.38 | (0.24, 0.52) | 0.07 |  |
|  | Judaism | 0.22 | (0.05, 0.39) | 0.08 |  |
|  | Sikhism | 0.00 | * | * |  |
|  | Baha'i | 0.33 | * | * |  |
|  | Shinto | 0.00 | * | * |  |
|  | Taoism | 1.00 | * | * |  |
|  | Primal, Animist, or Folk religion | 0.31 | (0.16, 0.47) | 0.08 |  |
|  | Some other religion | 0.38 | (0.28, 0.48) | 0.05 |  |
|  | No religion/Atheist/Agnostic | 0.24 | (0.23, 0.25) | 0.01 |  |

***Table S19a. Nationally representative descriptive statistics for Tanzania***

| **Characteristic** | **N = 9,075**^1^ |
| --- | --- |
| **Age group** |  |
| 18-24 | 2,284 (25%) |
| 25-29 | 1,349 (15%) |
| 30-39 | 2,060 (23%) |
| 40-49 | 1,503 (17%) |
| 50-59 | 912 (10%) |
| 60-69 | 575 (6.3%) |
| 70-79 | 297 (3.3%) |
| 80 or older | 93 (1.0%) |
| (Missing) | 2 (<0.1%) |
| **Gender** |  |
| Male | 4,299 (47%) |
| Female | 4,776 (53%) |
| Other | 0 (0%) |
| (Missing) | 0 (0%) |
| **Marital status** |  |
| Married | 5,577 (61%) |
| Separated | 404 (4.5%) |
| Divorced | 103 (1.1%) |
| Widowed | 450 (5.0%) |
| Never | 2,260 (25%) |
| Domestic Partner | 275 (3.0%) |
| (Missing) | 7 (<0.1%) |
| **Employment** |  |
| Employed for an employer | 513 (5.6%) |
| Self-employed | 4,625 (51%) |
| Retired | 139 (1.5%) |
| Student | 319 (3.5%) |
| Homemaker | 1,796 (20%) |
| Unemployed and looking for a job | 1,491 (16%) |
| None of these/Other | 186 (2.1%) |
| (Missing) | 6 (<0.1%) |
| **Religious service attendance** |  |
| At least 1/week | 2,622 (29%) |
| 1/week | 4,268 (47%) |
| 1-3/month | 1,082 (12%) |
| A few times a year | 814 (9.0%) |
| Never | 288 (3.2%) |
| (Missing) | 1 (<0.1%) |
| **Education** |  |
| Up to 8 years | 6,699 (74%) |
| 9-15 years | 2,252 (25%) |
| 16+ years | 122 (1.3%) |
| (Missing) | 2 (<0.1%) |
| **Immigration** |  |
| Born in this country | 9,048 (100%) |
| Born in another country | 25 (0.3%) |
| (Missing) | 1 (<0.1%) |
| **Religious affiliation** |  |
| Christianity | 5,647 (62%) |
| Islam | 3,189 (35%) |
| Hinduism | 0 (0%) |
| Buddhism | 0 (0%) |
| Judaism | 0 (0%) |
| Sikhism | 0 (0%) |
| Baha'i | 0 (0%) |
| Jainism | 0 (0%) |
| Shinto | 0 (0%) |
| Taoism | 1 (<0.1%) |
| Confucianism | 0 (0%) |
| Primal, Animist, or Folk religion | 12 (0.1%) |
| Spiritism | 0 (0%) |
| Umbanda, Candomble, and other African-derived religions | 0 (0%) |
| Chinese folk/traditional religion | 0 (0%) |
| Some other religion | 0 (0%) |
| No religion/Atheist/Agnostic | 216 (2.4%) |
| (Missing) | 10 (0.1%) |
| **Race/Ethnicity** |  |
| African | 9,060 (100%) |
| Arab | 11 (0.1%) |
| Indian | 3 (<0.1%) |
| (Missing) | 2 (<0.1%) |
| ^1^n (%) | |

***Table S19b. Proportions by demographic category for Tanzania***

| Variable | Category | Mean | 95% CI | SE | Global p-value |
| --- | --- | --- | --- | --- | --- |
| Age group | 18-24 | 0.06 | (0.05, 0.07) | 0.01 | < .001 |
|  | 25-29 | 0.10 | (0.08, 0.12) | 0.01 |  |
|  | 30-39 | 0.12 | (0.10, 0.14) | 0.01 |  |
|  | 40-49 | 0.16 | (0.14, 0.19) | 0.01 |  |
|  | 50-59 | 0.26 | (0.23, 0.29) | 0.02 |  |
|  | 60-69 | 0.38 | (0.33, 0.44) | 0.03 |  |
|  | 70-79 | 0.54 | (0.44, 0.64) | 0.05 |  |
|  | 80 or older | 0.59 | (0.42, 0.77) | 0.09 |  |
| Gender | Male | 0.12 | (0.11, 0.14) | 0.01 | < .001 |
|  | Female | 0.19 | (0.17, 0.21) | 0.01 |  |
| Marital status | Married | 0.16 | (0.15, 0.18) | 0.01 | < .001 |
|  | Separated | 0.22 | (0.17, 0.26) | 0.02 |  |
|  | Divorced | 0.28 | (0.18, 0.39) | 0.05 |  |
|  | Widowed | 0.49 | (0.42, 0.56) | 0.04 |  |
|  | Never | 0.07 | (0.06, 0.08) | 0.01 |  |
|  | Domestic Partner | 0.12 | (0.08, 0.16) | 0.02 |  |
| Employment | Employed for an employer | 0.06 | (0.04, 0.09) | 0.01 | < .001 |
|  | Self-employed | 0.16 | (0.14, 0.17) | 0.01 |  |
|  | Retired | 0.44 | (0.33, 0.55) | 0.06 |  |
|  | Student | 0.04 | (0.02, 0.06) | 0.01 |  |
|  | Homemaker | 0.23 | (0.20, 0.26) | 0.01 |  |
|  | Unemployed and looking for a job | 0.11 | (0.09, 0.12) | 0.01 |  |
|  | None of these/Other | 0.26 | (0.18, 0.34) | 0.04 |  |
| Religious service attendance | At least 1/week | 0.18 | (0.16, 0.20) | 0.01 | < .001 |
|  | 1/week | 0.15 | (0.13, 0.16) | 0.01 |  |
|  | 1-3/month | 0.17 | (0.14, 0.20) | 0.02 |  |
|  | A few times a year | 0.11 | (0.08, 0.14) | 0.01 |  |
|  | Never | 0.21 | (0.14, 0.27) | 0.03 |  |
| Education | Up to 8 years | 0.19 | (0.17, 0.20) | 0.01 | < .001 |
|  | 9-15 years | 0.08 | (0.07, 0.09) | 0.01 |  |
|  | 16+ years | 0.07 | (0.02, 0.11) | 0.02 |  |
| Immigration status | Born in this country | 0.16 | (0.15, 0.17) | 0.01 | 0.840 |
|  | Born in another country | 0.13 | (0.00, 0.39) | 0.12 |  |
| Religious affiliation | Christianity | 0.16 | (0.14, 0.17) | 0.01 | < .001 |
|  | Islam | 0.16 | (0.15, 0.18) | 0.01 |  |
|  | Taoism | 0.00 | * | * |  |
|  | Primal, Animist, or Folk religion | 0.32 | (0.00, 0.71) | 0.15 |  |
|  | No religion/Atheist/Agnostic | 0.18 | (0.13, 0.23) | 0.03 |  |
| Race/ethnicity | Indian | 0.43 | * | * | 0.569 |
|  | Arab | 0.13 | (0.00, 0.38) | 0.10 |  |
|  | African | 0.16 | (0.15, 0.17) | 0.01 |  |

***Table S20a. Nationally representative descriptive statistics for Turkey***

| **Characteristic** | **N = 1,473**^1^ |
| --- | --- |
| **Age group** |  |
| 18-24 | 222 (15%) |
| 25-29 | 152 (10%) |
| 30-39 | 315 (21%) |
| 40-49 | 312 (21%) |
| 50-59 | 225 (15%) |
| 60-69 | 164 (11%) |
| 70-79 | 65 (4.4%) |
| 80 or older | 18 (1.2%) |
| (Missing) | 0 (0%) |
| **Gender** |  |
| Male | 754 (51%) |
| Female | 719 (49%) |
| Other | 0 (0%) |
| (Missing) | 0 (0%) |
| **Marital status** |  |
| Married | 936 (64%) |
| Separated | 13 (0.9%) |
| Divorced | 64 (4.3%) |
| Widowed | 64 (4.3%) |
| Never | 379 (26%) |
| Domestic Partner | 0 (0%) |
| (Missing) | 17 (1.1%) |
| **Employment** |  |
| Employed for an employer | 413 (28%) |
| Self-employed | 255 (17%) |
| Retired | 205 (14%) |
| Student | 107 (7.3%) |
| Homemaker | 347 (24%) |
| Unemployed and looking for a job | 87 (5.9%) |
| None of these/Other | 59 (4.0%) |
| (Missing) | 0 (0%) |
| **Religious service attendance** |  |
| At least 1/week | 493 (33%) |
| 1/week | 271 (18%) |
| 1-3/month | 174 (12%) |
| A few times a year | 255 (17%) |
| Never | 274 (19%) |
| (Missing) | 6 (0.4%) |
| **Education** |  |
| Up to 8 years | 436 (30%) |
| 9-15 years | 711 (48%) |
| 16+ years | 326 (22%) |
| (Missing) | 0 (0%) |
| **Immigration** |  |
| Born in this country | 1,415 (96%) |
| Born in another country | 58 (4.0%) |
| (Missing) | 0 (0%) |
| **Religious affiliation** |  |
| Christianity | 2 (0.1%) |
| Islam | 1,381 (94%) |
| Hinduism | 0 (0%) |
| Buddhism | 1 (<0.1%) |
| Judaism | 1 (<0.1%) |
| Sikhism | 1 (<0.1%) |
| Baha'i | 0 (0%) |
| Jainism | 0 (0%) |
| Shinto | 0 (0%) |
| Taoism | 0 (0%) |
| Confucianism | 0 (0%) |
| Primal, Animist, or Folk religion | 1 (<0.1%) |
| Spiritism | 0 (0%) |
| Umbanda, Candomble, and other African-derived religions | 0 (0%) |
| Chinese folk/traditional religion | 0 (0%) |
| Some other religion | 1 (<0.1%) |
| No religion/Atheist/Agnostic | 66 (4.5%) |
| (Missing) | 19 (1.3%) |
| **Race/Ethnicity** |  |
| Albanian | 8 (0.5%) |
| Arab | 51 (3.5%) |
| Armenian | 1 (<0.1%) |
| Azeri | 9 (0.6%) |
| Bosnian | 5 (0.3%) |
| Circassian | 19 (1.3%) |
| Georgian | 4 (0.3%) |
| Greek | 1 (<0.1%) |
| Kurdish/Zaza | 252 (17%) |
| Laz | 25 (1.7%) |
| Other | 58 (3.9%) |
| Turkish | 1,030 (70%) |
| Uyghur | 1 (<0.1%) |
| (Missing) | 9 (0.6%) |
| ^1^n (%) | |

***Table S20b. Proportions by demographic category for Turkey***

| Variable | Category | Mean | 95% CI | SE | Global p-value |
| --- | --- | --- | --- | --- | --- |
| Age group | 18-24 | 0.10 | (0.06, 0.15) | 0.02 | < .001 |
|  | 25-29 | 0.06 | (0.01, 0.11) | 0.02 |  |
|  | 30-39 | 0.11 | (0.07, 0.15) | 0.02 |  |
|  | 40-49 | 0.16 | (0.11, 0.21) | 0.03 |  |
|  | 50-59 | 0.13 | (0.07, 0.19) | 0.03 |  |
|  | 60-69 | 0.21 | (0.11, 0.32) | 0.05 |  |
|  | 70-79 | 0.30 | (0.11, 0.49) | 0.10 |  |
|  | 80 or older | 0.01 | (0.00, 0.16) | 0.04 |  |
| Gender | Male | 0.12 | (0.09, 0.15) | 0.01 | 0.144 |
|  | Female | 0.15 | (0.12, 0.19) | 0.02 |  |
| Marital status | Married | 0.13 | (0.10, 0.16) | 0.02 | 0.112 |
|  | Separated | 0.26 | (0.00, 0.68) | 0.17 |  |
|  | Divorced | 0.27 | (0.12, 0.41) | 0.07 |  |
|  | Widowed | 0.21 | (0.04, 0.38) | 0.09 |  |
|  | Never | 0.11 | (0.08, 0.14) | 0.02 |  |
| Employment | Employed for an employer | 0.09 | (0.06, 0.12) | 0.02 | 0.012 |
|  | Self-employed | 0.10 | (0.05, 0.15) | 0.02 |  |
|  | Retired | 0.22 | (0.14, 0.31) | 0.04 |  |
|  | Student | 0.08 | (0.03, 0.12) | 0.02 |  |
|  | Homemaker | 0.18 | (0.12, 0.24) | 0.03 |  |
|  | Unemployed and looking for a job | 0.15 | (0.08, 0.22) | 0.03 |  |
|  | None of these/Other | 0.16 | (0.05, 0.26) | 0.05 |  |
| Religious service attendance | At least 1/week | 0.16 | (0.11, 0.20) | 0.02 | 0.060 |
|  | 1/week | 0.10 | (0.05, 0.15) | 0.02 |  |
|  | 1-3/month | 0.09 | (0.04, 0.13) | 0.02 |  |
|  | A few times a year | 0.13 | (0.07, 0.18) | 0.03 |  |
|  | Never | 0.18 | (0.12, 0.24) | 0.03 |  |
| Education | Up to 8 years | 0.18 | (0.13, 0.24) | 0.03 | < .001 |
|  | 9-15 years | 0.13 | (0.10, 0.17) | 0.02 |  |
|  | 16+ years | 0.08 | (0.06, 0.10) | 0.01 |  |
| Immigration status | Born in this country | 0.14 | (0.11, 0.16) | 0.01 | 0.768 |
|  | Born in another country | 0.12 | (0.00, 0.24) | 0.06 |  |
| Religious affiliation | Christianity | 0.00 | * | * | 0.811 |
|  | Islam | 0.14 | (0.11, 0.16) | 0.01 |  |
|  | Buddhism | 0.00 | * | * |  |
|  | Judaism | 0.00 | * | * |  |
|  | Sikhism | 0.00 | * | * |  |
|  | Primal, Animist, or Folk religion | 0.57 | * | * |  |
|  | Some other religion | 0.58 | * | * |  |
|  | No religion/Atheist/Agnostic | 0.13 | (0.04, 0.23) | 0.05 |  |
| Race/ethnicity | Arab | 0.11 | (0.00, 0.22) | 0.06 |  |
|  | Turkish | 0.14 | (0.11, 0.17) | 0.01 |  |
|  | Greek | 0.00 | * | * |  |
|  | Kurdish/Zaza | 0.15 | (0.09, 0.20) | 0.03 |  |
|  | Laz | 0.15 | (0.00, 0.34) | 0.09 |  |
|  | Circassian | 0.07 | (0.00, 0.21) | 0.05 |  |
|  | Bosnian | 0.00 | * | * |  |
|  | Armenian | 0.00 | * | * |  |
|  | Georgian | 0.15 | * | * |  |
|  | Uyghur | 0.00 | * | * |  |
|  | Albanian | 0.00 | * | * |  |
|  | Azeri | 0.00 | * | * |  |
|  | Other | 0.16 | (0.02, 0.29) | 0.07 |  |

***Table S21a. Nationally representative descriptive statistics for United Kingdom***

| **Characteristic** | **N = 5,368**^1^ |
| --- | --- |
| **Age group** |  |
| 18-24 | 490 (9.1%) |
| 25-29 | 391 (7.3%) |
| 30-39 | 946 (18%) |
| 40-49 | 827 (15%) |
| 50-59 | 949 (18%) |
| 60-69 | 889 (17%) |
| 70-79 | 711 (13%) |
| 80 or older | 163 (3.0%) |
| (Missing) | 1 (<0.1%) |
| **Gender** |  |
| Male | 2,557 (48%) |
| Female | 2,789 (52%) |
| Other | 14 (0.3%) |
| (Missing) | 9 (0.2%) |
| **Marital status** |  |
| Married | 2,510 (47%) |
| Separated | 114 (2.1%) |
| Divorced | 435 (8.1%) |
| Widowed | 294 (5.5%) |
| Never | 1,456 (27%) |
| Domestic Partner | 512 (9.5%) |
| (Missing) | 48 (0.9%) |
| **Employment** |  |
| Employed for an employer | 2,798 (52%) |
| Self-employed | 469 (8.7%) |
| Retired | 1,262 (24%) |
| Student | 229 (4.3%) |
| Homemaker | 184 (3.4%) |
| Unemployed and looking for a job | 215 (4.0%) |
| None of these/Other | 201 (3.7%) |
| (Missing) | 11 (0.2%) |
| **Religious service attendance** |  |
| At least 1/week | 291 (5.4%) |
| 1/week | 499 (9.3%) |
| 1-3/month | 293 (5.5%) |
| A few times a year | 1,165 (22%) |
| Never | 3,110 (58%) |
| (Missing) | 10 (0.2%) |
| **Education** |  |
| Up to 8 years | 1,314 (24%) |
| 9-15 years | 2,072 (39%) |
| 16+ years | 1,974 (37%) |
| (Missing) | 8 (0.2%) |
| **Immigration** |  |
| Born in this country | 4,659 (87%) |
| Born in another country | 682 (13%) |
| (Missing) | 27 (0.5%) |
| **Religious affiliation** |  |
| Christianity | 2,750 (51%) |
| Islam | 218 (4.1%) |
| Hinduism | 61 (1.1%) |
| Buddhism | 30 (0.6%) |
| Judaism | 44 (0.8%) |
| Sikhism | 29 (0.5%) |
| Baha'i | 6 (0.1%) |
| Jainism | 4 (<0.1%) |
| Shinto | 0 (0%) |
| Taoism | 4 (<0.1%) |
| Confucianism | 2 (<0.1%) |
| Primal, Animist, or Folk religion | 36 (0.7%) |
| Spiritism | 0 (0%) |
| Umbanda, Candomble, and other African-derived religions | 0 (0%) |
| Chinese folk/traditional religion | 0 (0%) |
| Some other religion | 61 (1.1%) |
| No religion/Atheist/Agnostic | 2,099 (39%) |
| (Missing) | 25 (0.5%) |
| **Race/Ethnicity** |  |
| Asian | 426 (7.9%) |
| Black | 152 (2.8%) |
| Other | 96 (1.8%) |
| White | 4,647 (87%) |
| (Missing) | 47 (0.9%) |
| ^1^n (%) | |

***Table S21b. Proportions by demographic category for United Kingdom***

| Variable | Category | Mean | 95% CI | SE | Global p-value |
| --- | --- | --- | --- | --- | --- |
| Age group | 18-24 | 0.22 | (0.16, 0.27) | 0.03 | < .001 |
|  | 25-29 | 0.22 | (0.16, 0.28) | 0.03 |  |
|  | 30-39 | 0.22 | (0.19, 0.26) | 0.02 |  |
|  | 40-49 | 0.24 | (0.20, 0.28) | 0.02 |  |
|  | 50-59 | 0.31 | (0.27, 0.35) | 0.02 |  |
|  | 60-69 | 0.43 | (0.39, 0.48) | 0.02 |  |
|  | 70-79 | 0.42 | (0.36, 0.47) | 0.03 |  |
|  | 80 or older | 0.46 | (0.35, 0.56) | 0.05 |  |
| Gender | Male | 0.28 | (0.26, 0.31) | 0.01 | 0.009 |
|  | Female | 0.33 | (0.31, 0.36) | 0.01 |  |
|  | Other | 0.57 | (0.09, 1.00) | 0.21 |  |
| Marital status | Married | 0.29 | (0.26, 0.31) | 0.01 | < .001 |
|  | Separated | 0.34 | (0.23, 0.45) | 0.06 |  |
|  | Divorced | 0.45 | (0.39, 0.52) | 0.03 |  |
|  | Widowed | 0.46 | (0.38, 0.54) | 0.04 |  |
|  | Never | 0.28 | (0.25, 0.31) | 0.02 |  |
|  | Domestic Partner | 0.27 | (0.21, 0.32) | 0.03 |  |
| Employment | Employed for an employer | 0.22 | (0.20, 0.24) | 0.01 | < .001 |
|  | Self-employed | 0.23 | (0.18, 0.28) | 0.02 |  |
|  | Retired | 0.44 | (0.40, 0.48) | 0.02 |  |
|  | Student | 0.21 | (0.13, 0.29) | 0.04 |  |
|  | Homemaker | 0.35 | (0.25, 0.46) | 0.05 |  |
|  | Unemployed and looking for a job | 0.42 | (0.32, 0.52) | 0.05 |  |
|  | None of these/Other | 0.86 | (0.80, 0.92) | 0.03 |  |
| Religious service attendance | At least 1/week | 0.25 | (0.18, 0.32) | 0.03 | < .001 |
|  | 1/week | 0.27 | (0.22, 0.33) | 0.03 |  |
|  | 1-3/month | 0.20 | (0.14, 0.27) | 0.03 |  |
|  | A few times a year | 0.30 | (0.27, 0.34) | 0.02 |  |
|  | Never | 0.33 | (0.31, 0.35) | 0.01 |  |
| Education | Up to 8 years | 0.43 | (0.38, 0.48) | 0.03 | < .001 |
|  | 9-15 years | 0.31 | (0.29, 0.34) | 0.01 |  |
|  | 16+ years | 0.22 | (0.20, 0.24) | 0.01 |  |
| Immigration status | Born in this country | 0.33 | (0.31, 0.35) | 0.01 | < .001 |
|  | Born in another country | 0.19 | (0.15, 0.22) | 0.02 |  |
| Religious affiliation | Christianity | 0.32 | (0.30, 0.35) | 0.01 | < .001 |
|  | Islam | 0.18 | (0.11, 0.25) | 0.04 |  |
|  | Hinduism | 0.19 | (0.07, 0.32) | 0.06 |  |
|  | Buddhism | 0.31 | (0.12, 0.50) | 0.09 |  |
|  | Judaism | 0.40 | (0.19, 0.61) | 0.10 |  |
|  | Sikhism | 0.33 | (0.02, 0.65) | 0.15 |  |
|  | Baha'i | 0.06 | * | * |  |
|  | Jainism | 0.00 | * | * |  |
|  | Taoism | 0.58 | * | * |  |
|  | Confucianism | 1.00 | * | * |  |
|  | Primal, Animist, or Folk religion | 0.39 | (0.13, 0.65) | 0.12 |  |
|  | Some other religion | 0.46 | (0.31, 0.62) | 0.08 |  |
|  | No religion/Atheist/Agnostic | 0.30 | (0.27, 0.32) | 0.01 |  |
| Race/ethnicity | Asian | 0.19 | (0.14, 0.25) | 0.03 | < .001 |
|  | Black | 0.19 | (0.11, 0.27) | 0.04 |  |
|  | White | 0.33 | (0.31, 0.34) | 0.01 |  |
|  | Other | 0.19 | (0.07, 0.30) | 0.06 |  |

***Table S22a. Nationally representative descriptive statistics for United States***

| **Characteristic** | **N = 38,312**^1^ |
| --- | --- |
| **Age group** |  |
| 18-24 | 2,682 (7.0%) |
| 25-29 | 3,540 (9.2%) |
| 30-39 | 7,284 (19%) |
| 40-49 | 5,649 (15%) |
| 50-59 | 6,745 (18%) |
| 60-69 | 6,832 (18%) |
| 70-79 | 4,054 (11%) |
| 80 or older | 1,525 (4.0%) |
| (Missing) | 0 (0%) |
| **Gender** |  |
| Male | 18,222 (48%) |
| Female | 19,562 (51%) |
| Other | 392 (1.0%) |
| (Missing) | 136 (0.4%) |
| **Marital status** |  |
| Married | 20,360 (53%) |
| Separated | 727 (1.9%) |
| Divorced | 3,636 (9.5%) |
| Widowed | 1,978 (5.2%) |
| Never | 9,431 (25%) |
| Domestic Partner | 1,971 (5.1%) |
| (Missing) | 207 (0.5%) |
| **Employment** |  |
| Employed for an employer | 19,502 (51%) |
| Self-employed | 3,445 (9.0%) |
| Retired | 9,016 (24%) |
| Student | 1,145 (3.0%) |
| Homemaker | 2,049 (5.3%) |
| Unemployed and looking for a job | 1,777 (4.6%) |
| None of these/Other | 1,292 (3.4%) |
| (Missing) | 87 (0.2%) |
| **Religious service attendance** |  |
| At least 1/week | 2,633 (6.9%) |
| 1/week | 5,887 (15%) |
| 1-3/month | 2,819 (7.4%) |
| A few times a year | 8,870 (23%) |
| Never | 17,975 (47%) |
| (Missing) | 128 (0.3%) |
| **Education** |  |
| Up to 8 years | 210 (0.5%) |
| 9-15 years | 25,322 (66%) |
| 16+ years | 12,705 (33%) |
| (Missing) | 75 (0.2%) |
| **Immigration** |  |
| Born in this country | 34,865 (91%) |
| Born in another country | 3,020 (7.9%) |
| (Missing) | 427 (1.1%) |
| **Religious affiliation** |  |
| Christianity | 22,954 (60%) |
| Islam | 205 (0.5%) |
| Hinduism | 167 (0.4%) |
| Buddhism | 336 (0.9%) |
| Judaism | 638 (1.7%) |
| Sikhism | 24 (<0.1%) |
| Baha'i | 13 (<0.1%) |
| Jainism | 18 (<0.1%) |
| Shinto | 12 (<0.1%) |
| Taoism | 93 (0.2%) |
| Confucianism | 8 (<0.1%) |
| Primal, Animist, or Folk religion | 240 (0.6%) |
| Spiritism | 0 (0%) |
| Umbanda, Candomble, and other African-derived religions | 0 (0%) |
| Chinese folk/traditional religion | 0 (0%) |
| Some other religion | 1,267 (3.3%) |
| No religion/Atheist/Agnostic | 11,870 (31%) |
| (Missing) | 467 (1.2%) |
| **Race/Ethnicity** |  |
| Asian | 2,466 (6.4%) |
| Black | 4,501 (12%) |
| Hispanic | 6,724 (18%) |
| Other | 997 (2.6%) |
| White | 23,605 (62%) |
| (Missing) | 20 (<0.1%) |
| ^1^n (%) | |

***Table S22b. Proportions by demographic category for United States***

| Variable | Category | Mean | 95% CI | SE | Global p-value |
| --- | --- | --- | --- | --- | --- |
| Age group | 18-24 | 0.20 | (0.13, 0.27) | 0.04 | < .001 |
|  | 25-29 | 0.24 | (0.18, 0.29) | 0.03 |  |
|  | 30-39 | 0.18 | (0.15, 0.20) | 0.01 |  |
|  | 40-49 | 0.22 | (0.19, 0.24) | 0.01 |  |
|  | 50-59 | 0.26 | (0.24, 0.28) | 0.01 |  |
|  | 60-69 | 0.28 | (0.27, 0.29) | 0.01 |  |
|  | 70-79 | 0.31 | (0.30, 0.33) | 0.01 |  |
|  | 80 or older | 0.37 | (0.33, 0.41) | 0.02 |  |
| Gender | Male | 0.22 | (0.20, 0.23) | 0.01 | < .001 |
|  | Female | 0.27 | (0.26, 0.28) | 0.01 |  |
|  | Other | 0.29 | (0.16, 0.43) | 0.07 |  |
| Marital status | Married | 0.21 | (0.20, 0.22) | 0.01 | < .001 |
|  | Separated | 0.32 | (0.22, 0.42) | 0.05 |  |
|  | Divorced | 0.30 | (0.28, 0.33) | 0.01 |  |
|  | Widowed | 0.37 | (0.33, 0.40) | 0.02 |  |
|  | Never | 0.26 | (0.23, 0.28) | 0.02 |  |
|  | Domestic Partner | 0.27 | (0.22, 0.33) | 0.03 |  |
| Employment | Employed for an employer | 0.16 | (0.15, 0.17) | 0.01 | < .001 |
|  | Self-employed | 0.21 | (0.17, 0.25) | 0.02 |  |
|  | Retired | 0.35 | (0.33, 0.36) | 0.01 |  |
|  | Student | 0.24 | (0.14, 0.33) | 0.05 |  |
|  | Homemaker | 0.32 | (0.27, 0.36) | 0.02 |  |
|  | Unemployed and looking for a job | 0.36 | (0.26, 0.46) | 0.05 |  |
|  | None of these/Other | 0.62 | (0.53, 0.71) | 0.05 |  |
| Religious service attendance | At least 1/week | 0.22 | (0.19, 0.26) | 0.02 | < .001 |
|  | 1/week | 0.22 | (0.20, 0.23) | 0.01 |  |
|  | 1-3/month | 0.21 | (0.17, 0.25) | 0.02 |  |
|  | A few times a year | 0.24 | (0.22, 0.26) | 0.01 |  |
|  | Never | 0.27 | (0.25, 0.28) | 0.01 |  |
| Education | Up to 8 years | 0.28 | (0.05, 0.50) | 0.11 | < .001 |
|  | 9-15 years | 0.28 | (0.26, 0.29) | 0.01 |  |
|  | 16+ years | 0.18 | (0.17, 0.18) | 0.00 |  |
| Immigration status | Born in this country | 0.25 | (0.24, 0.26) | 0.01 | < .001 |
|  | Born in another country | 0.15 | (0.11, 0.19) | 0.02 |  |
| Religious affiliation | Christianity | 0.25 | (0.23, 0.26) | 0.01 | < .001 |
|  | Islam | 0.22 | (0.07, 0.38) | 0.08 |  |
|  | Hinduism | 0.17 | (0.05, 0.29) | 0.06 |  |
|  | Buddhism | 0.15 | (0.10, 0.20) | 0.03 |  |
|  | Judaism | 0.22 | (0.17, 0.27) | 0.02 |  |
|  | Sikhism | 0.20 | (0.00, 0.50) | 0.13 |  |
|  | Baha'i | 0.32 | * | * |  |
|  | Jainism | 0.73 | (0.02, 1.00) | 0.19 |  |
|  | Shinto | 0.07 | (0.00, 0.24) | 0.07 |  |
|  | Taoism | 0.13 | (0.00, 0.27) | 0.07 |  |
|  | Confucianism | 0.05 | * | * |  |
|  | Primal, Animist, or Folk religion | 0.35 | (0.14, 0.56) | 0.11 |  |
|  | Some other religion | 0.42 | (0.34, 0.51) | 0.04 |  |
|  | No religion/Atheist/Agnostic | 0.22 | (0.21, 0.24) | 0.01 |  |
| Race/ethnicity | Asian | 0.18 | (0.14, 0.22) | 0.02 | < .001 |
|  | Black | 0.23 | (0.20, 0.26) | 0.02 |  |
|  | White | 0.25 | (0.24, 0.26) | 0.00 |  |
|  | Other | 0.33 | (0.28, 0.39) | 0.03 |  |
|  | Hispanic | 0.24 | (0.20, 0.28) | 0.02 |  |

| ***Table S23.*** *Population weighted meta-analysis of results demographic group means.* | | | | |
| --- | --- | --- | --- | --- |
| Variable | Category | Proportion | 95% CI of Proportion | SE Analogue (CI Width/4) |
| Age group |  |  |  |  |
|  | 18-24 | 0.15 | (0.14,0.17) | 0.01 |
|  | 25-29 | 0.16 | (0.15,0.18) | 0.01 |
|  | 30-39 | 0.18 | (0.16,0.20) | 0.01 |
|  | 40-49 | 0.24 | (0.22,0.26) | 0.01 |
|  | 50-59 | 0.29 | (0.27,0.32) | 0.01 |
|  | 60-69 | 0.36 | (0.33,0.38) | 0.01 |
|  | 70-79 | 0.40 | (0.38,0.43) | 0.01 |
|  | 80 or older | 0.42 | (0.39,0.45) | 0.02 |
| Gender |  |  |  |  |
|  | Male | 0.21 | (0.19,0.24) | 0.01 |
|  | Female | 0.25 | (0.22,0.27) | 0.01 |
|  | Other | 0.12 | (0.10,0.14) | 0.01 |
| Marital status |  |  |  |  |
|  | Married | 0.23 | (0.21,0.25) | 0.01 |
|  | Separated | 0.26 | (0.24,0.29) | 0.01 |
|  | Divorced | 0.28 | (0.26,0.31) | 0.01 |
|  | Widowed | 0.40 | (0.37,0.43) | 0.01 |
|  | Domestic partner | 0.21 | (0.18,0.23) | 0.01 |
|  | Single, never married | 0.18 | (0.16,0.19) | 0.01 |
| Employment status |  |  |  |  |
|  | Employed for an employer | 0.19 | (0.17,0.21) | 0.01 |
|  | Self-employed | 0.20 | (0.18,0.22) | 0.01 |
|  | Retired | 0.40 | (0.37,0.42) | 0.01 |
|  | Student | 0.15 | (0.13,0.16) | 0.01 |
|  | Homemaker | 0.27 | (0.25,0.29) | 0.01 |
|  | Unemployed and looking for a job | 0.26 | (0.24,0.28) | 0.01 |
|  | None of these/other | 0.36 | (0.34,0.39) | 0.01 |
| Education |  |  |  |  |
|  | Up to 8 years | 0.28 | (0.25,0.30) | 0.01 |
|  | 9-15 years | 0.21 | (0.19,0.23) | 0.01 |
|  | 16+ years | 0.17 | (0.15,0.18) | 0.01 |
| Religious service attendance |  |  |  |  |
|  | >1/week | 0.24 | (0.22,0.27) | 0.01 |
|  | 1/week | 0.24 | (0.21,0.26) | 0.01 |
|  | 1-3/month | 0.23 | (0.21,0.25) | 0.01 |
|  | A few times a year | 0.21 | (0.19,0.24) | 0.01 |
|  | Never | 0.25 | (0.23,0.27) | 0.01 |
| Immigration status |  |  |  |  |
|  | Born in this country | 0.23 | (0.21,0.25) | 0.01 |
|  | Born in another country | 0.22 | (0.20,0.24) | 0.01 |


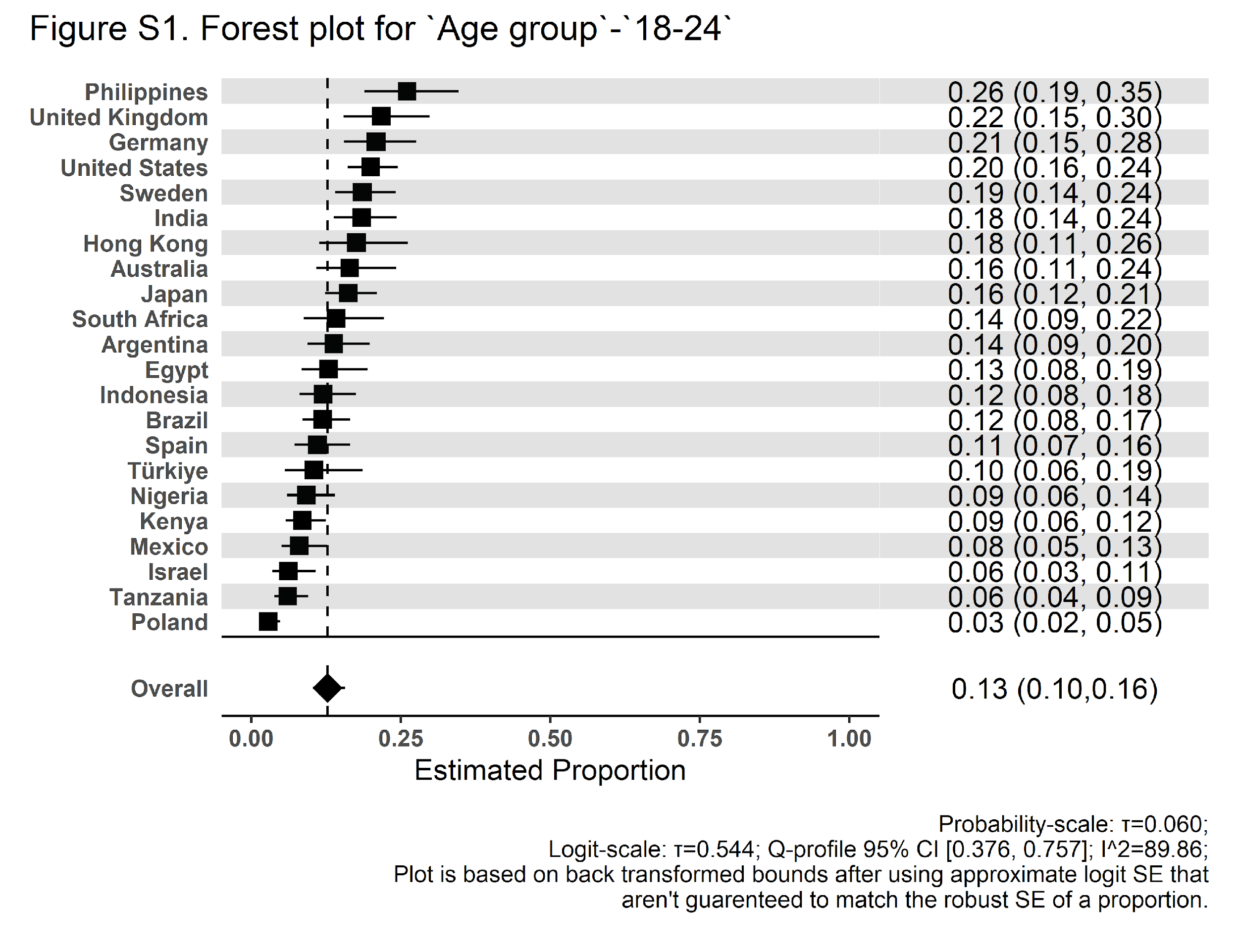

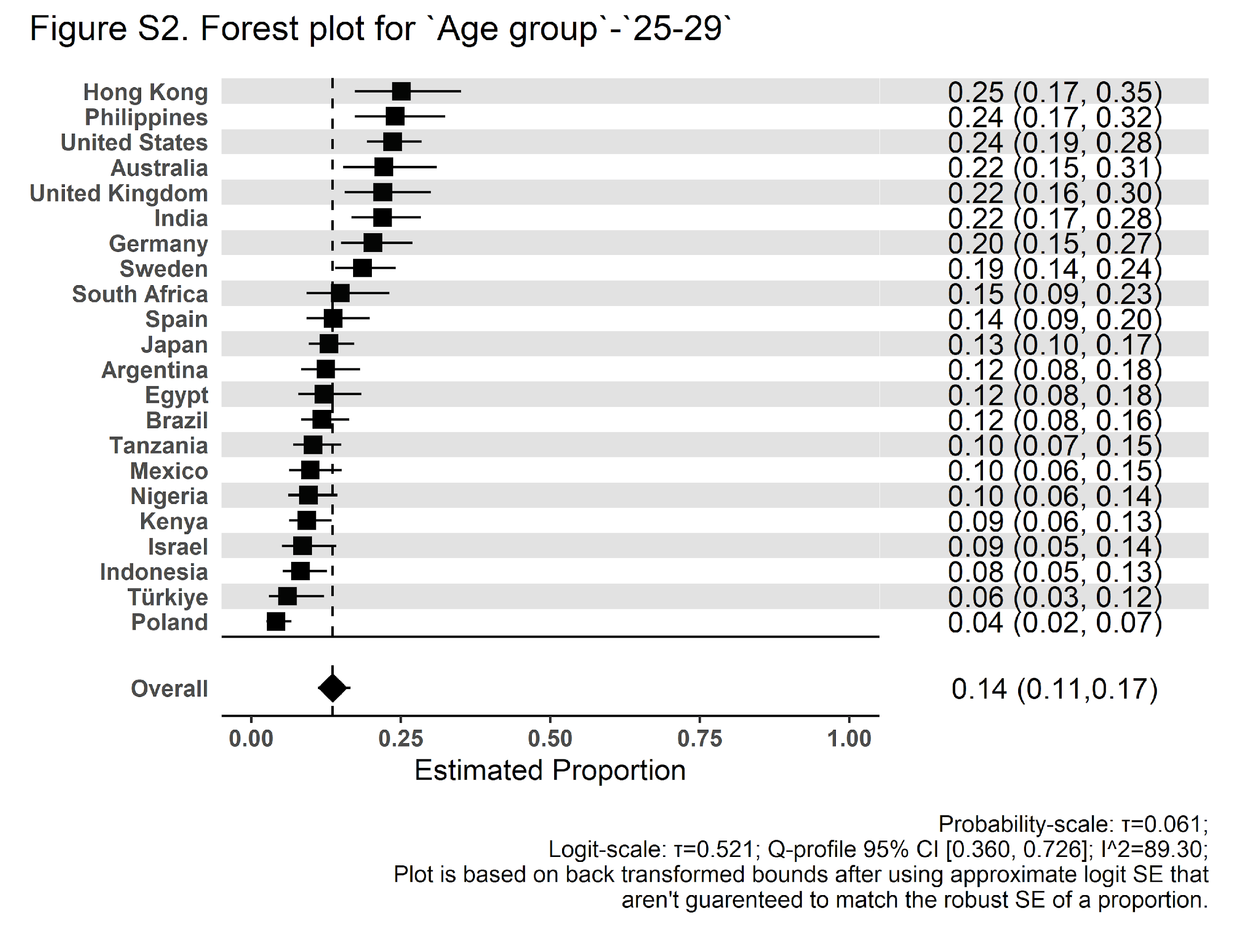

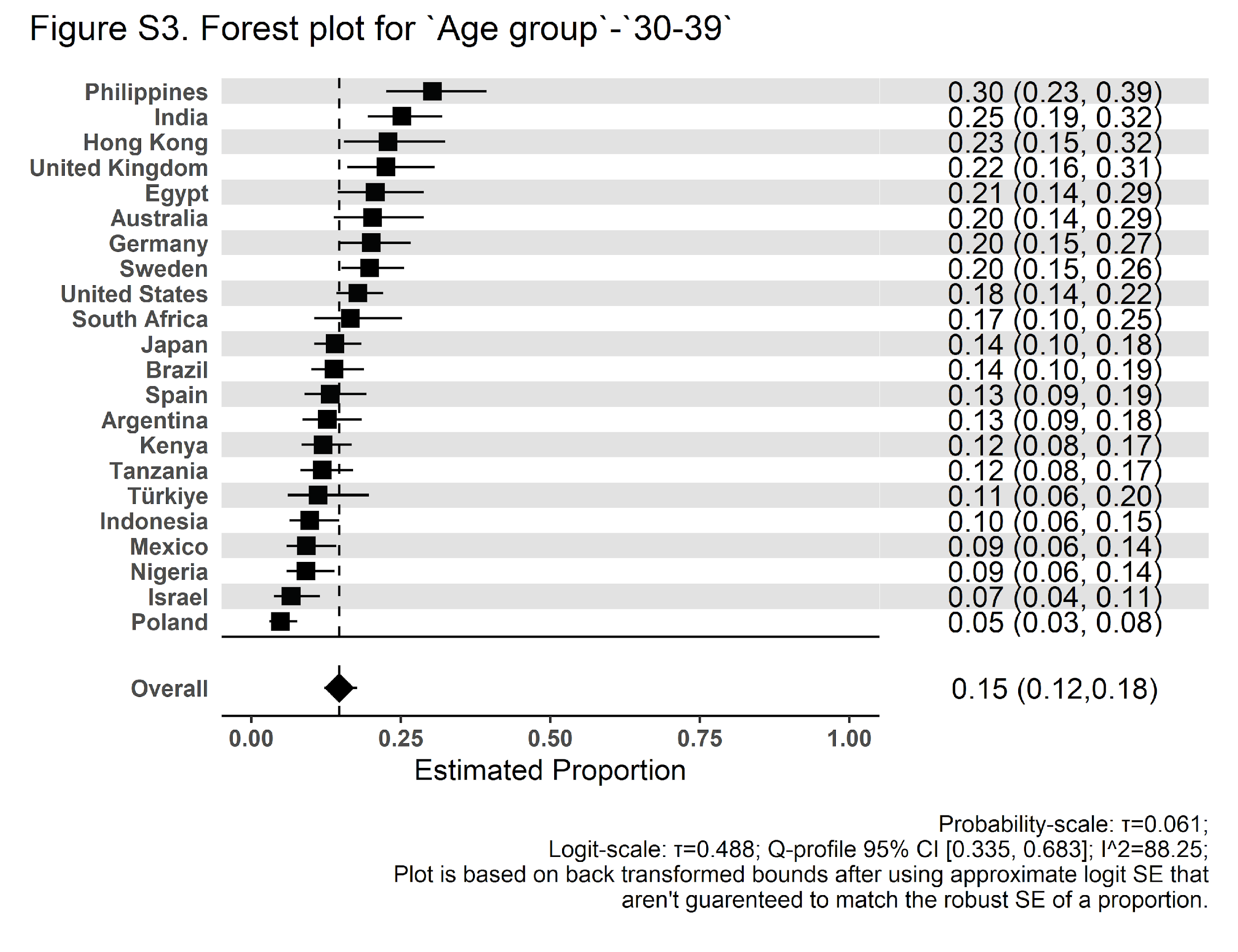

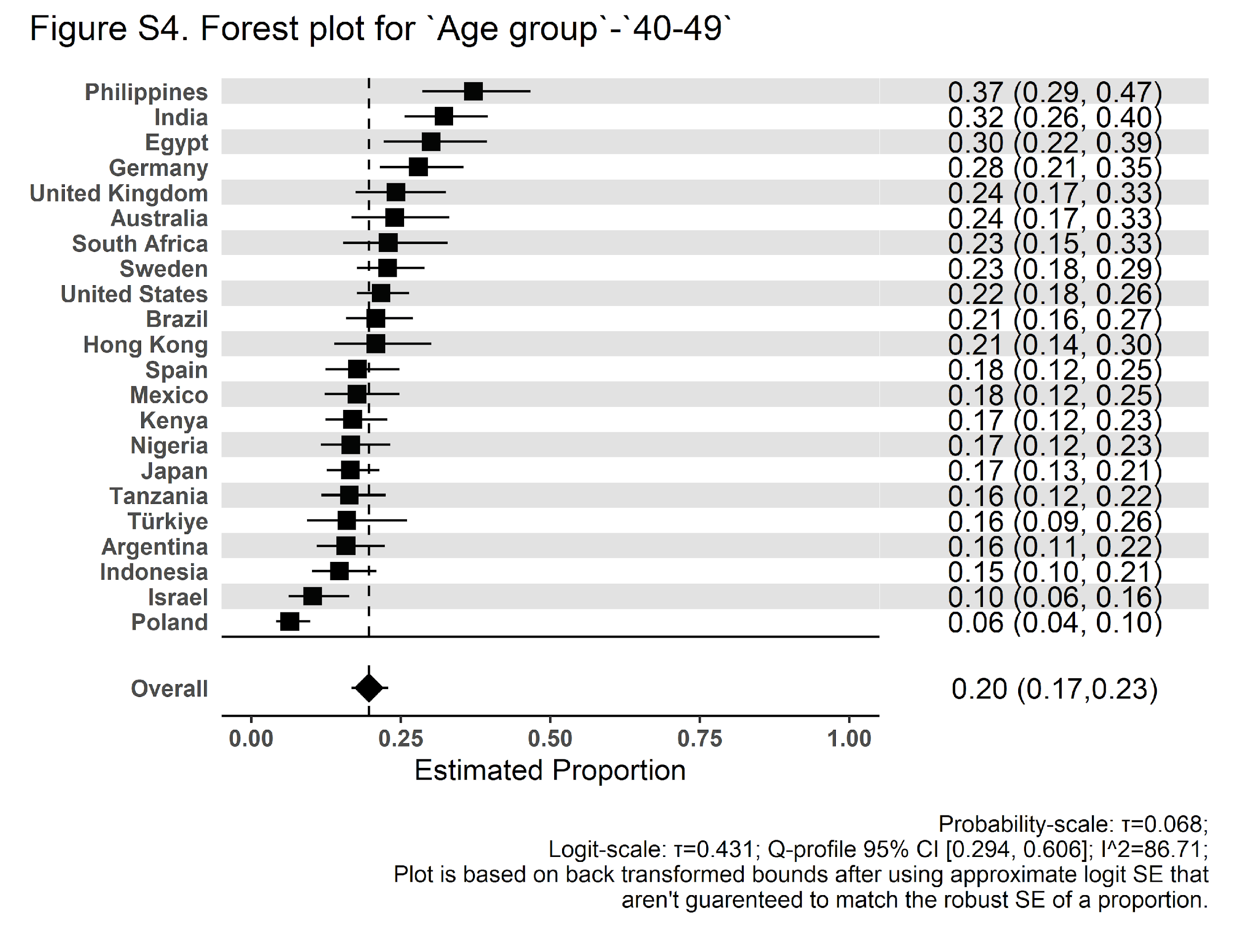

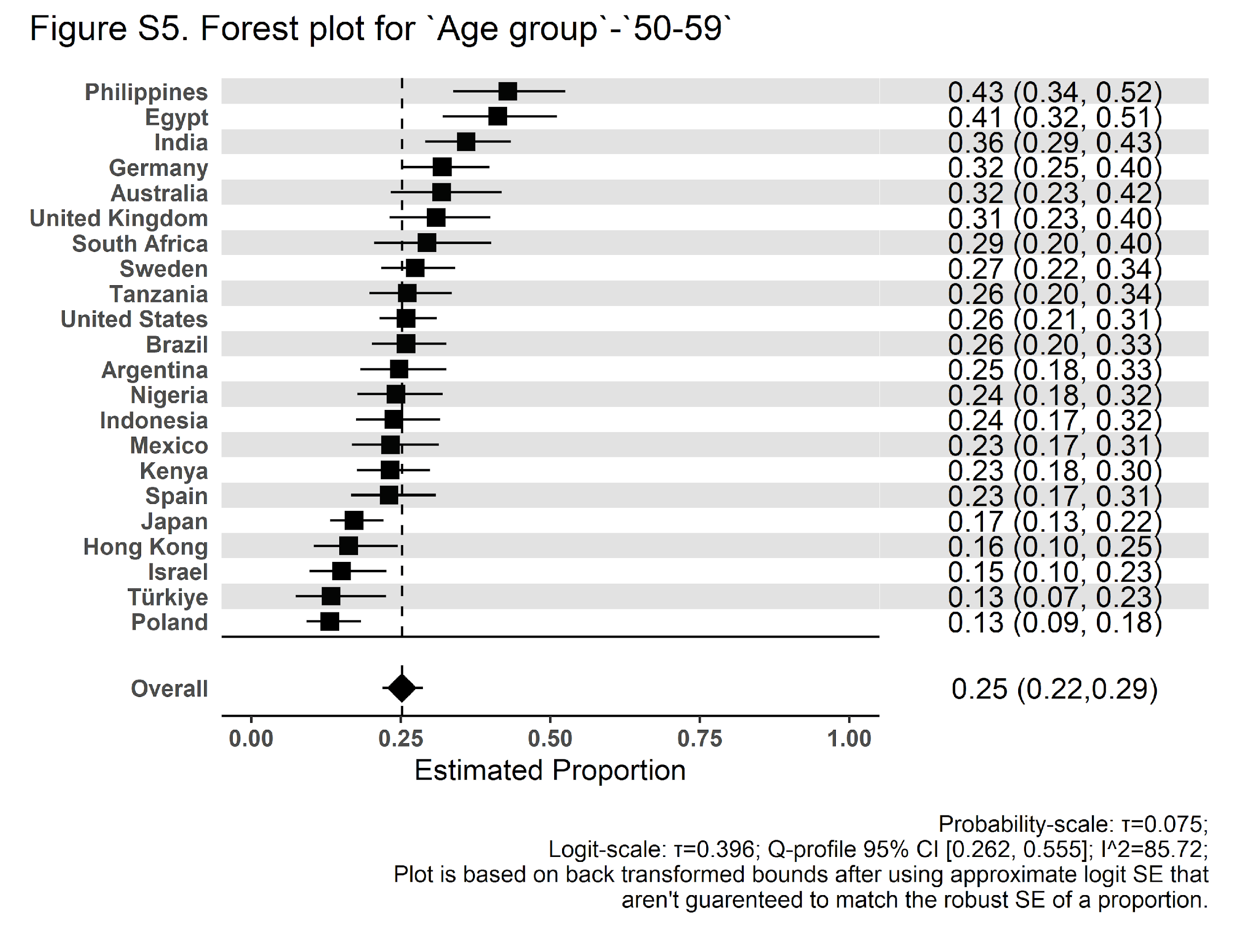

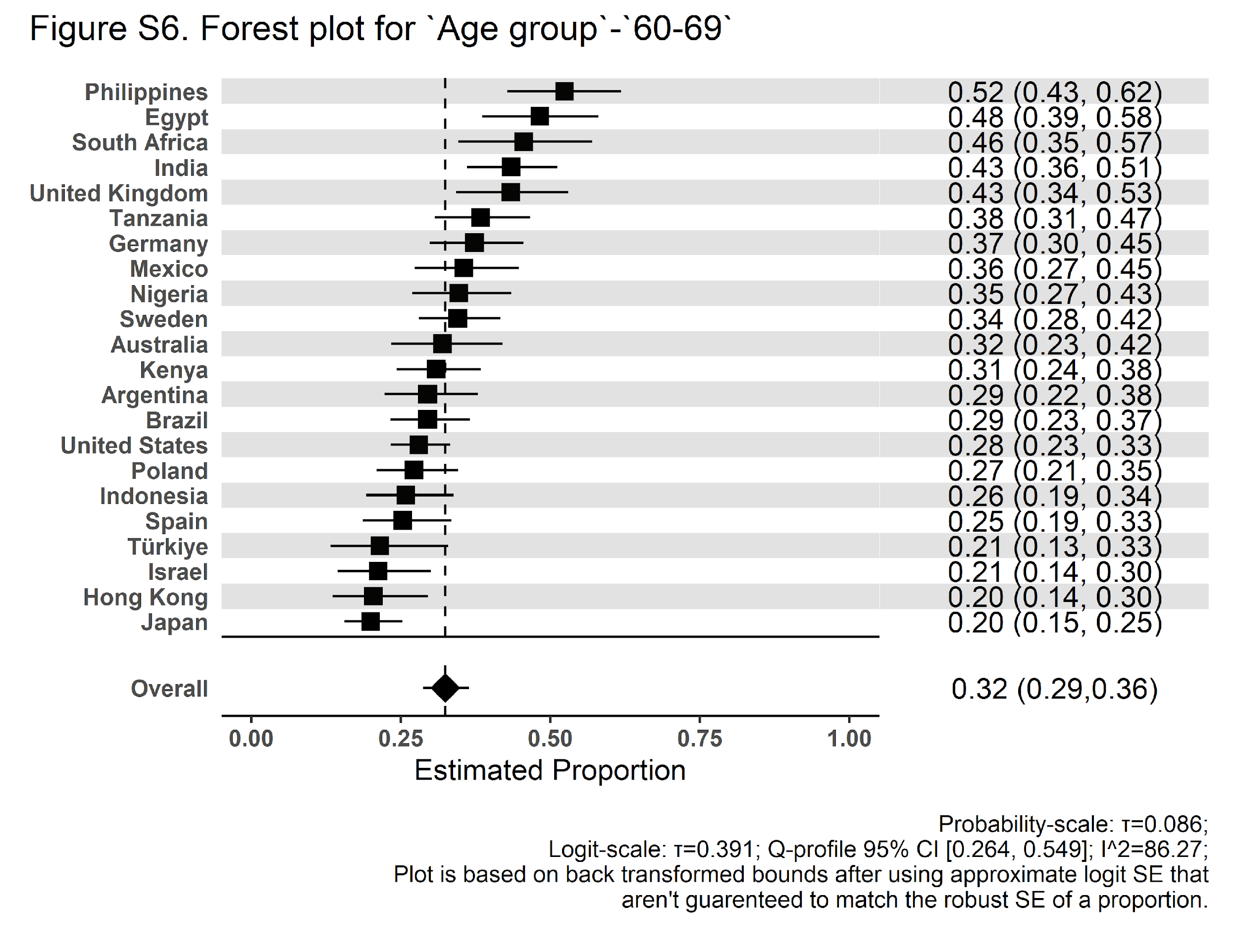

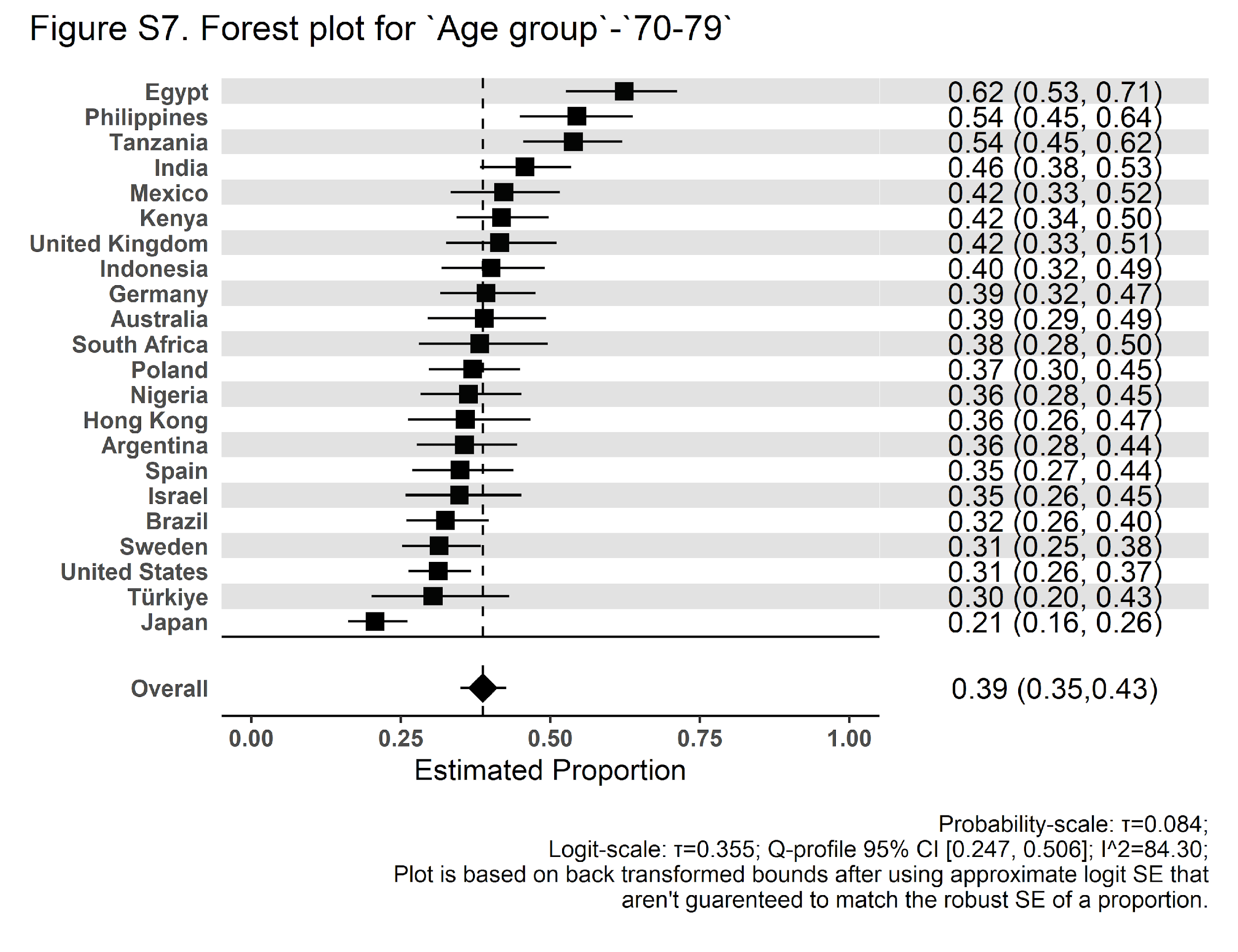

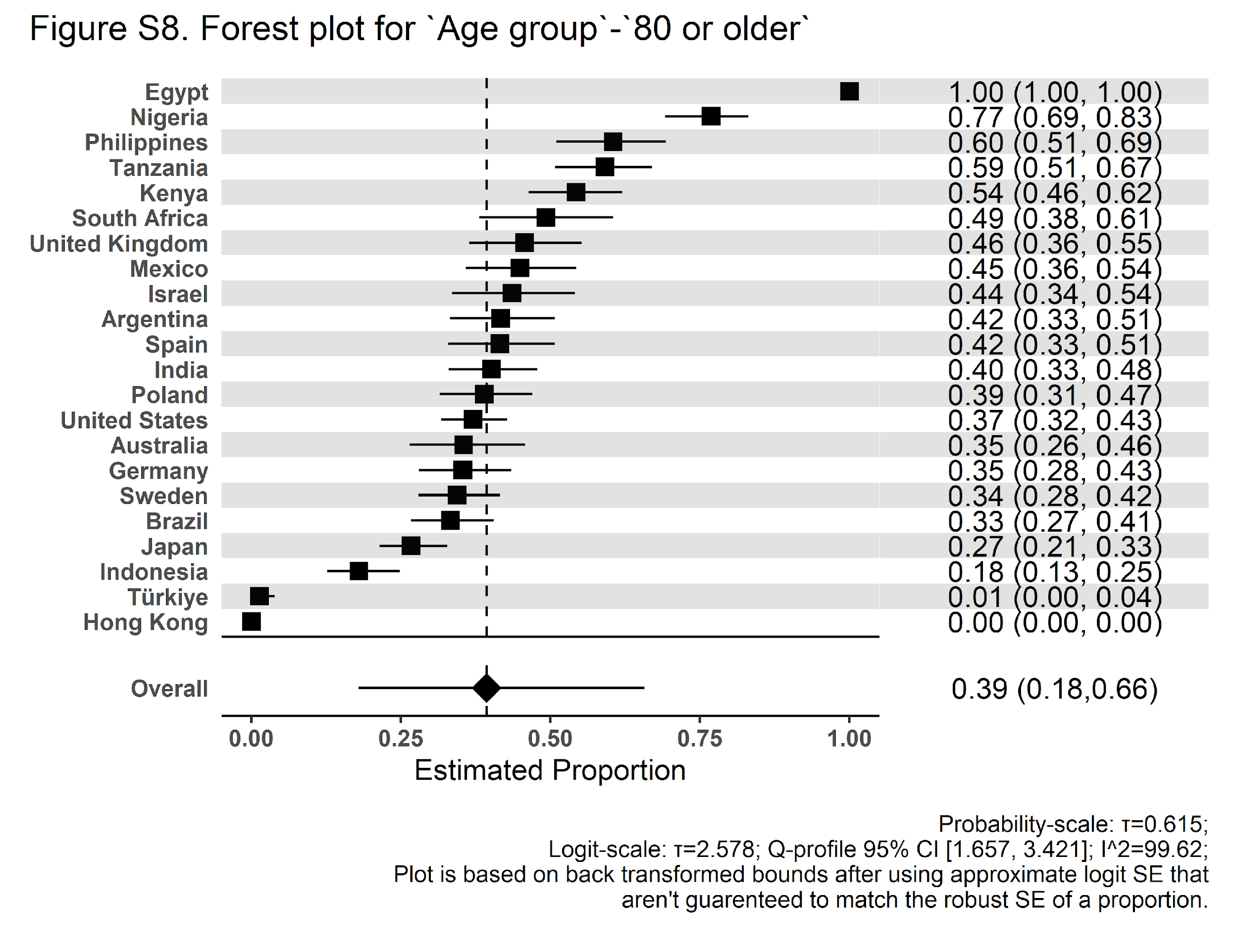

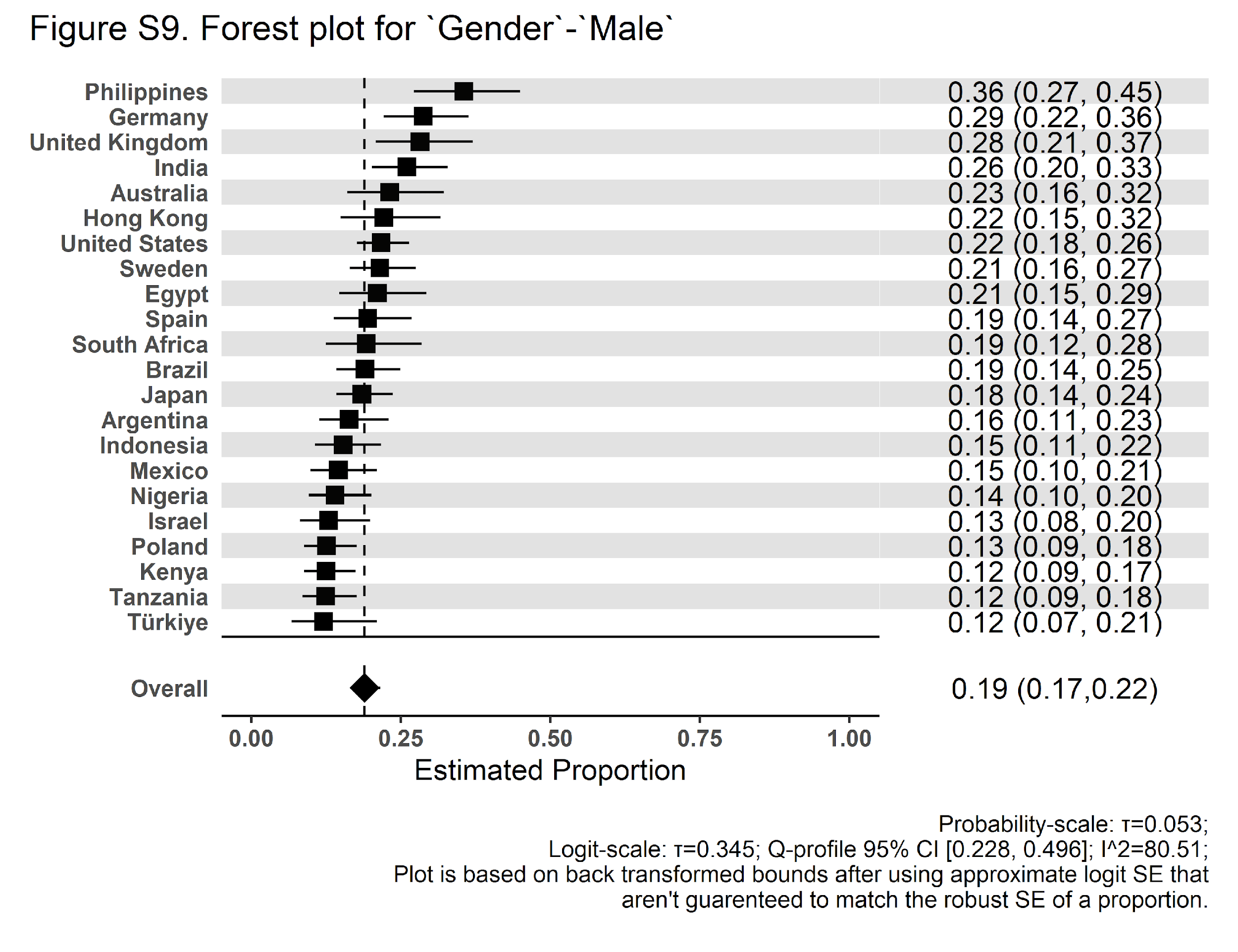

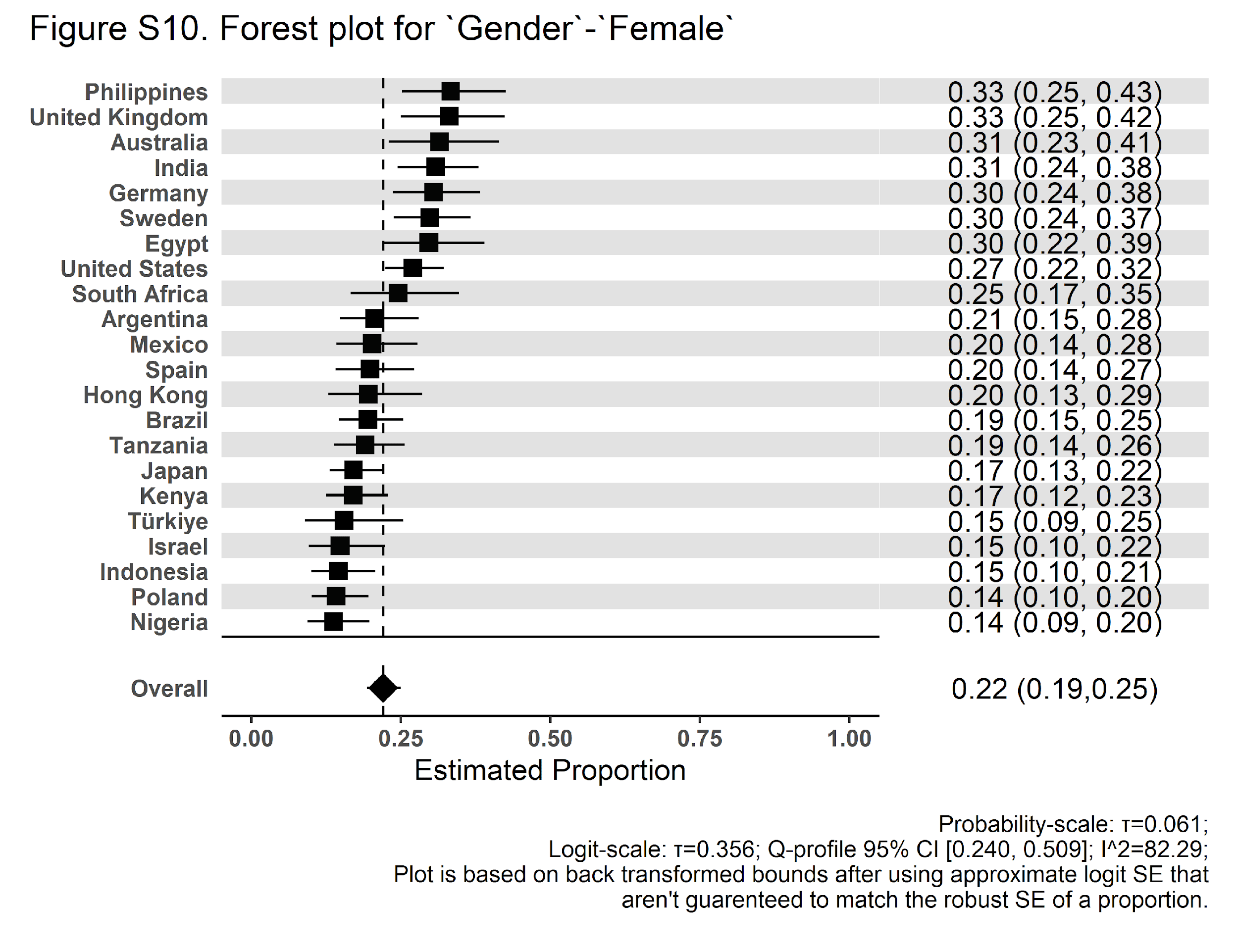

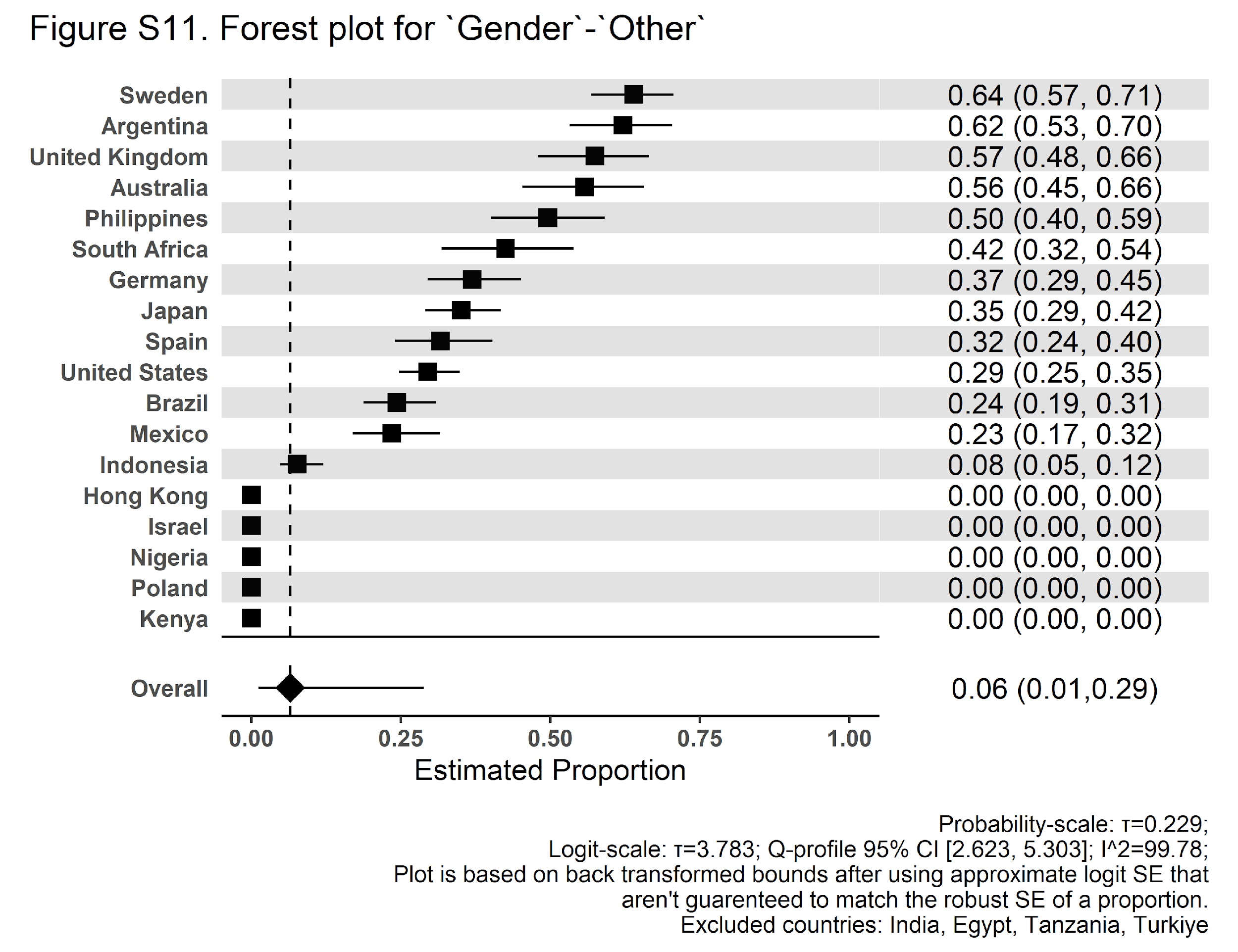

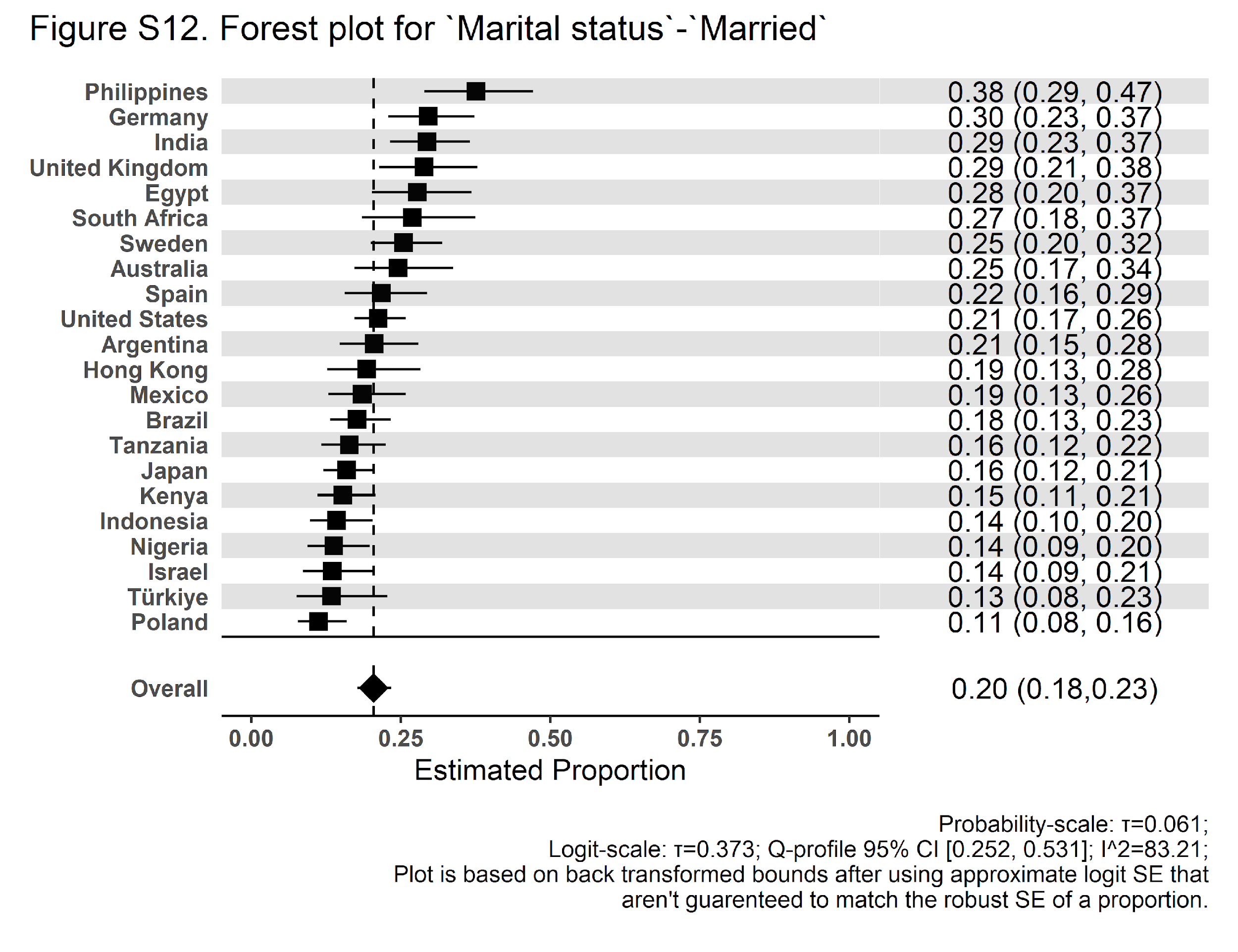

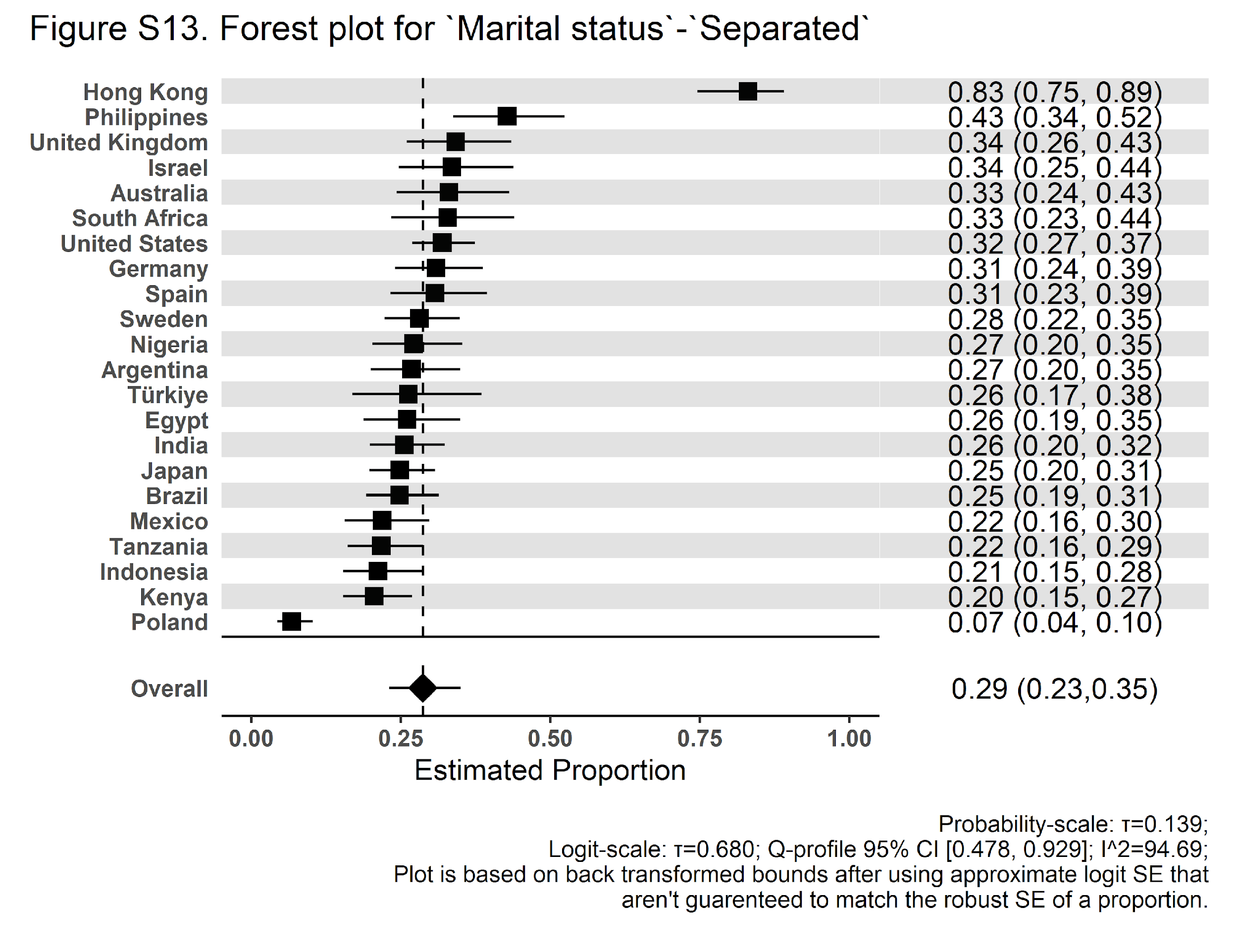

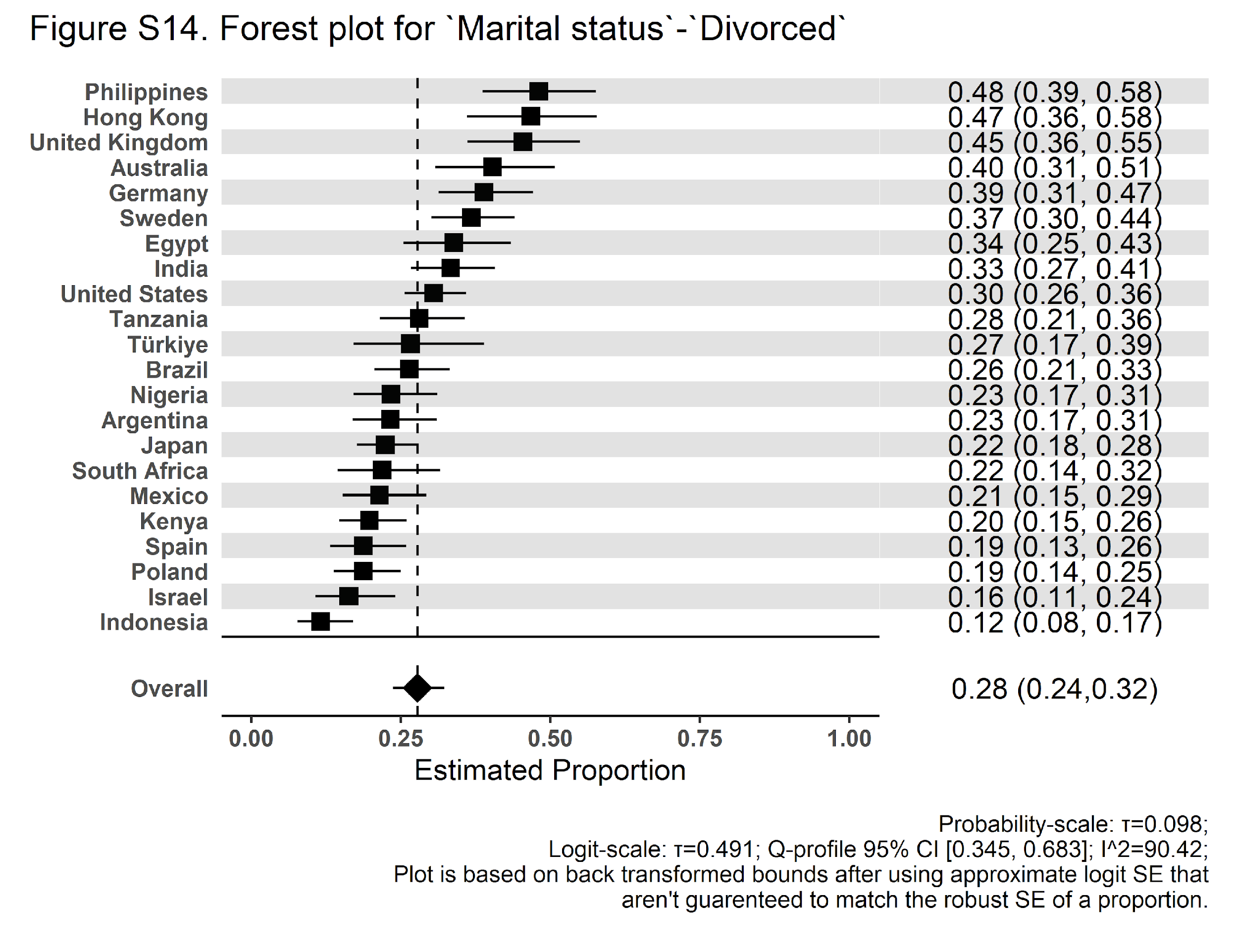

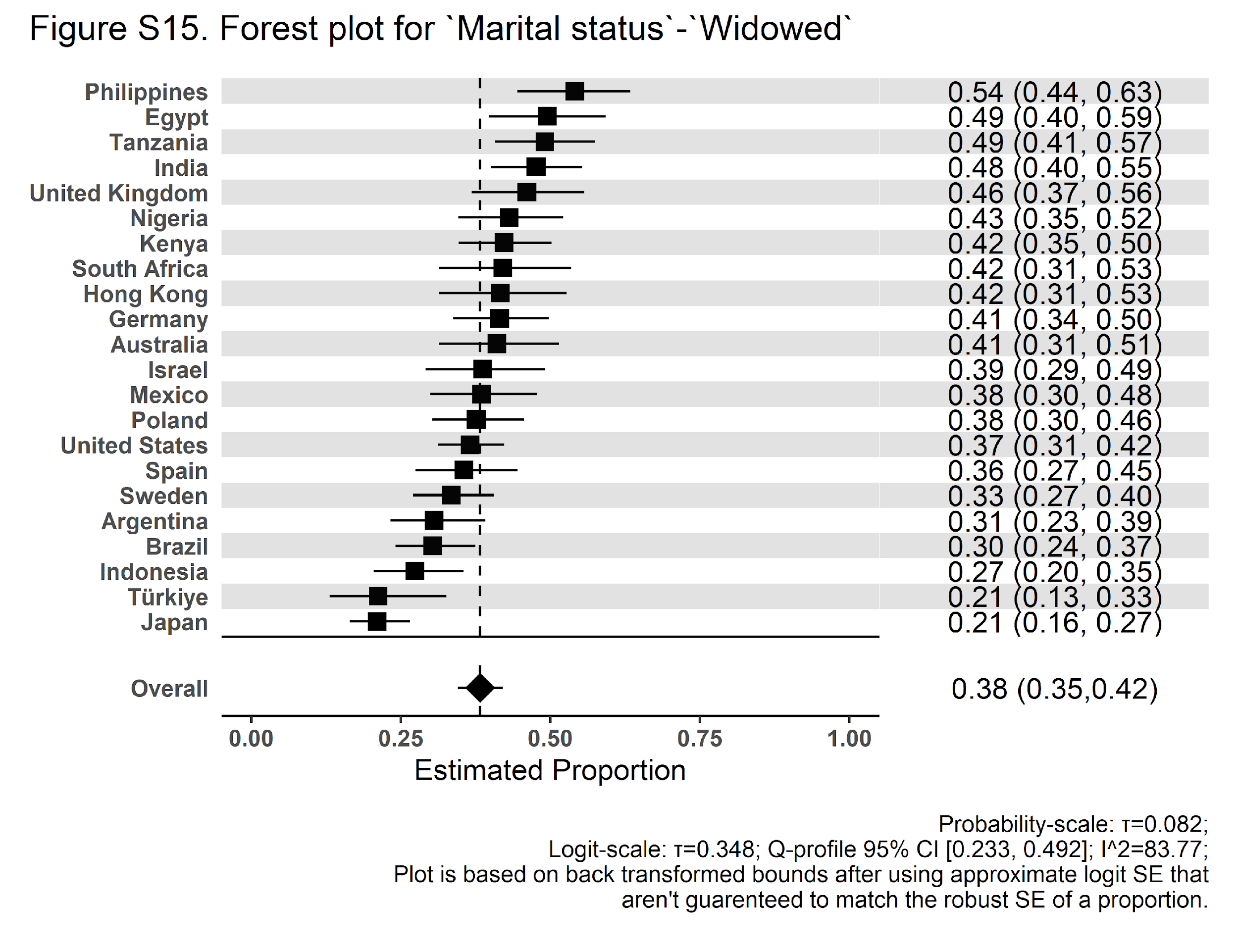

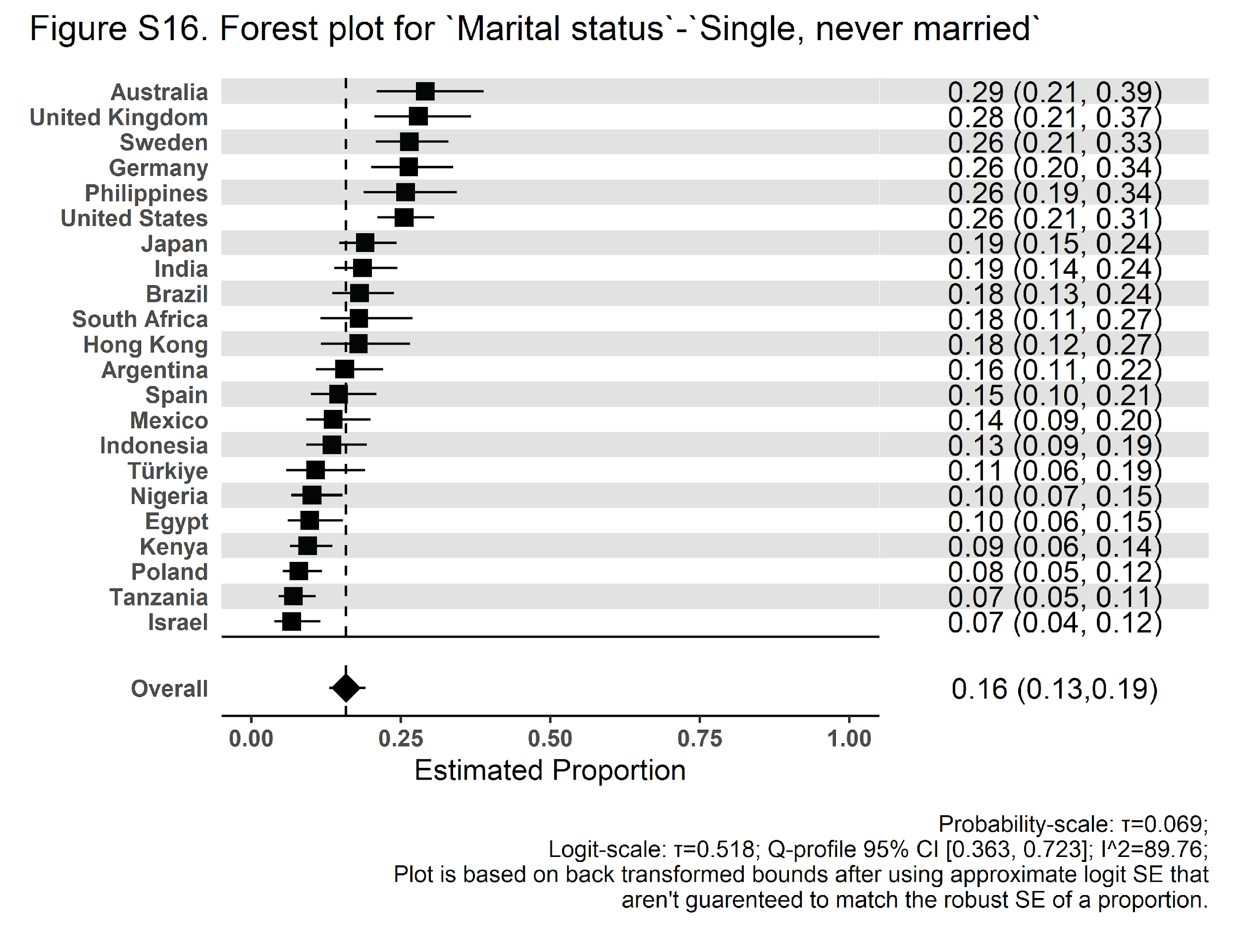

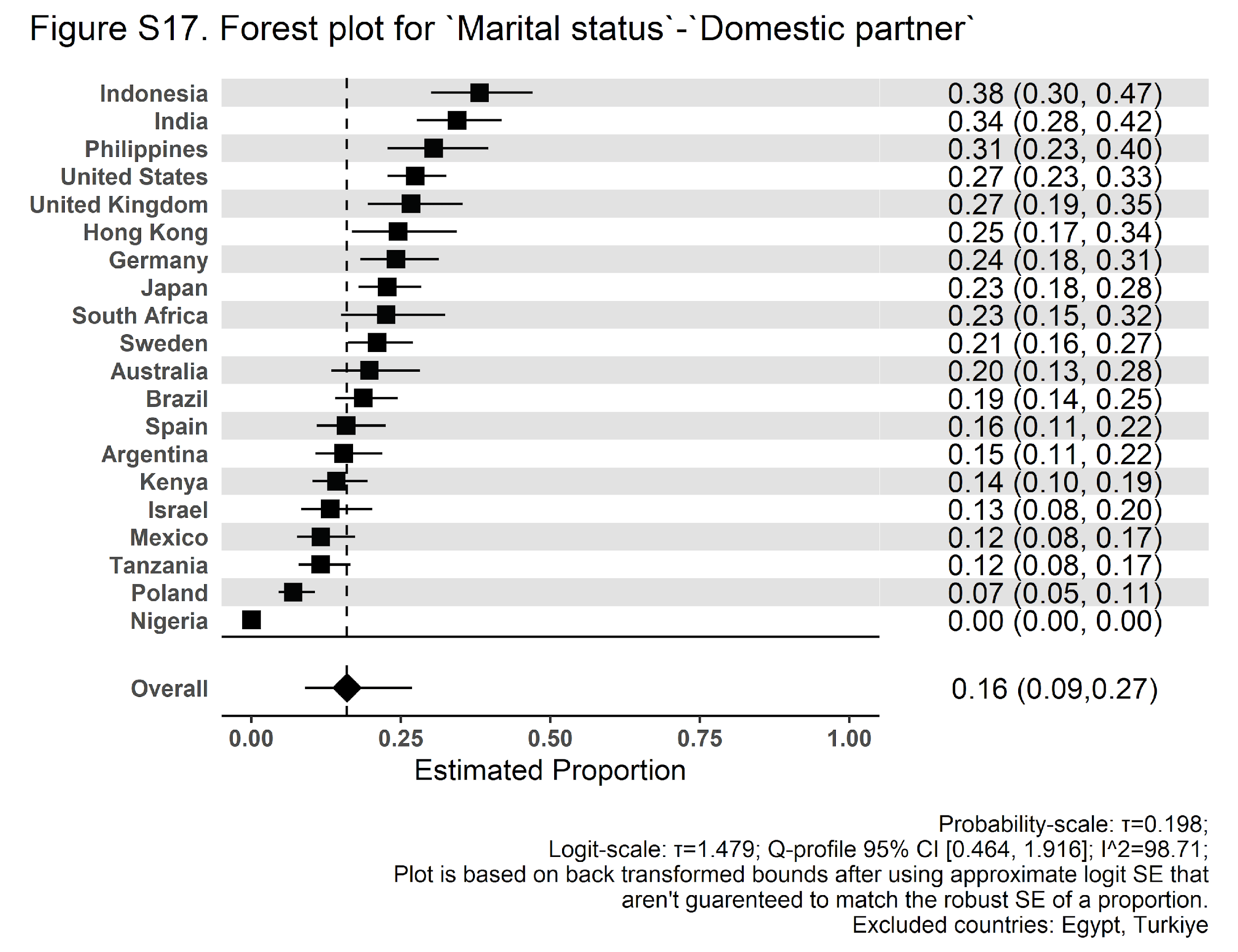

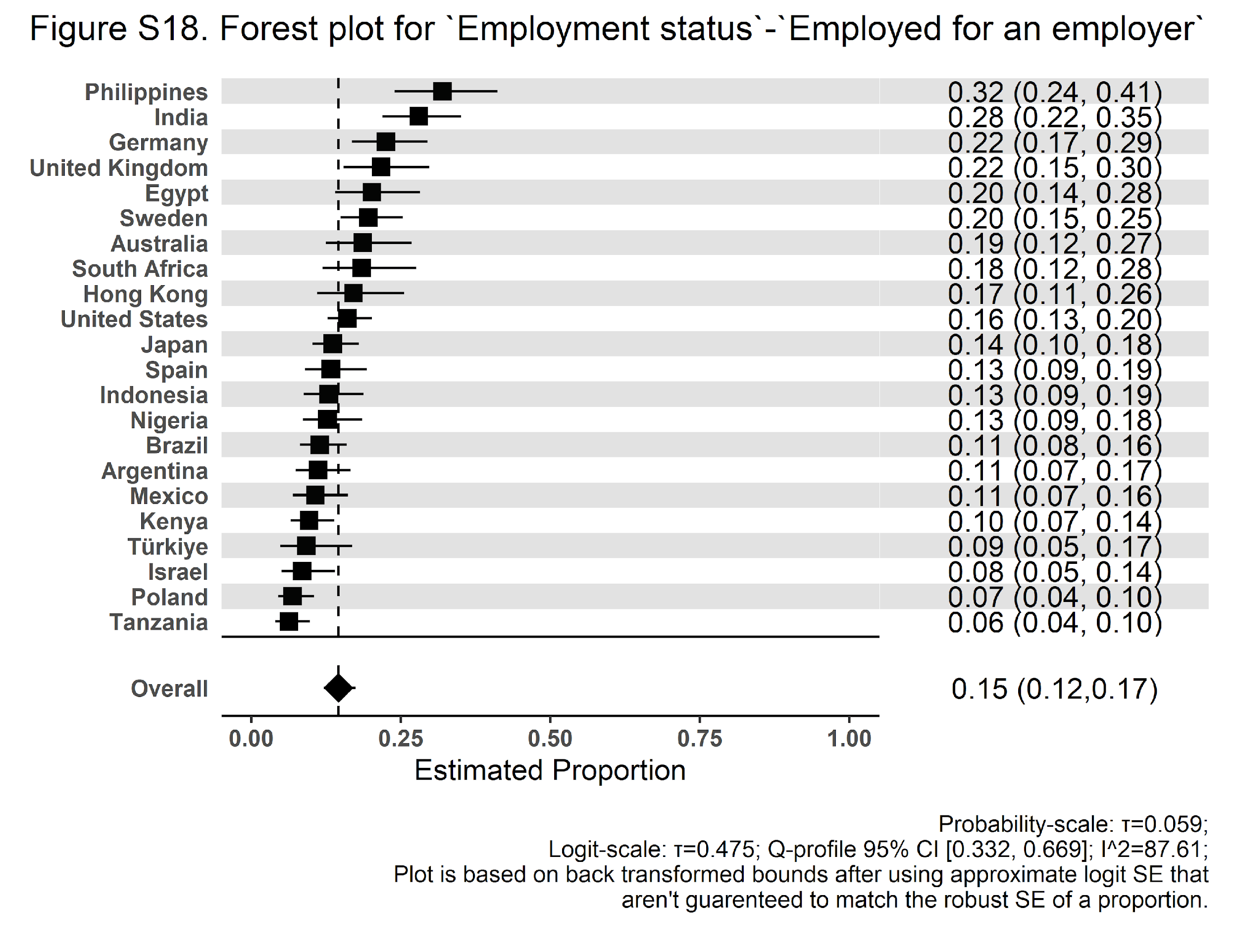

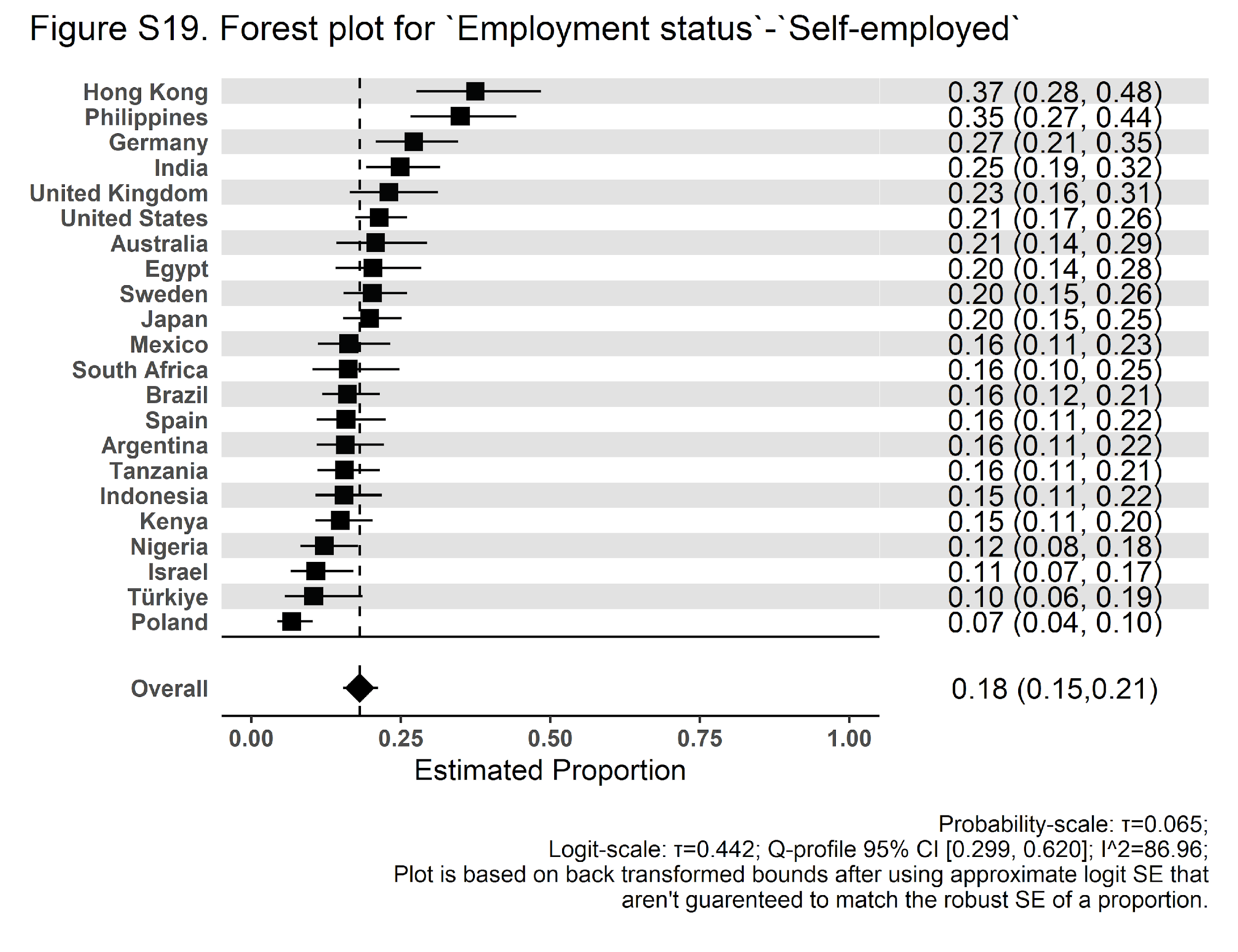

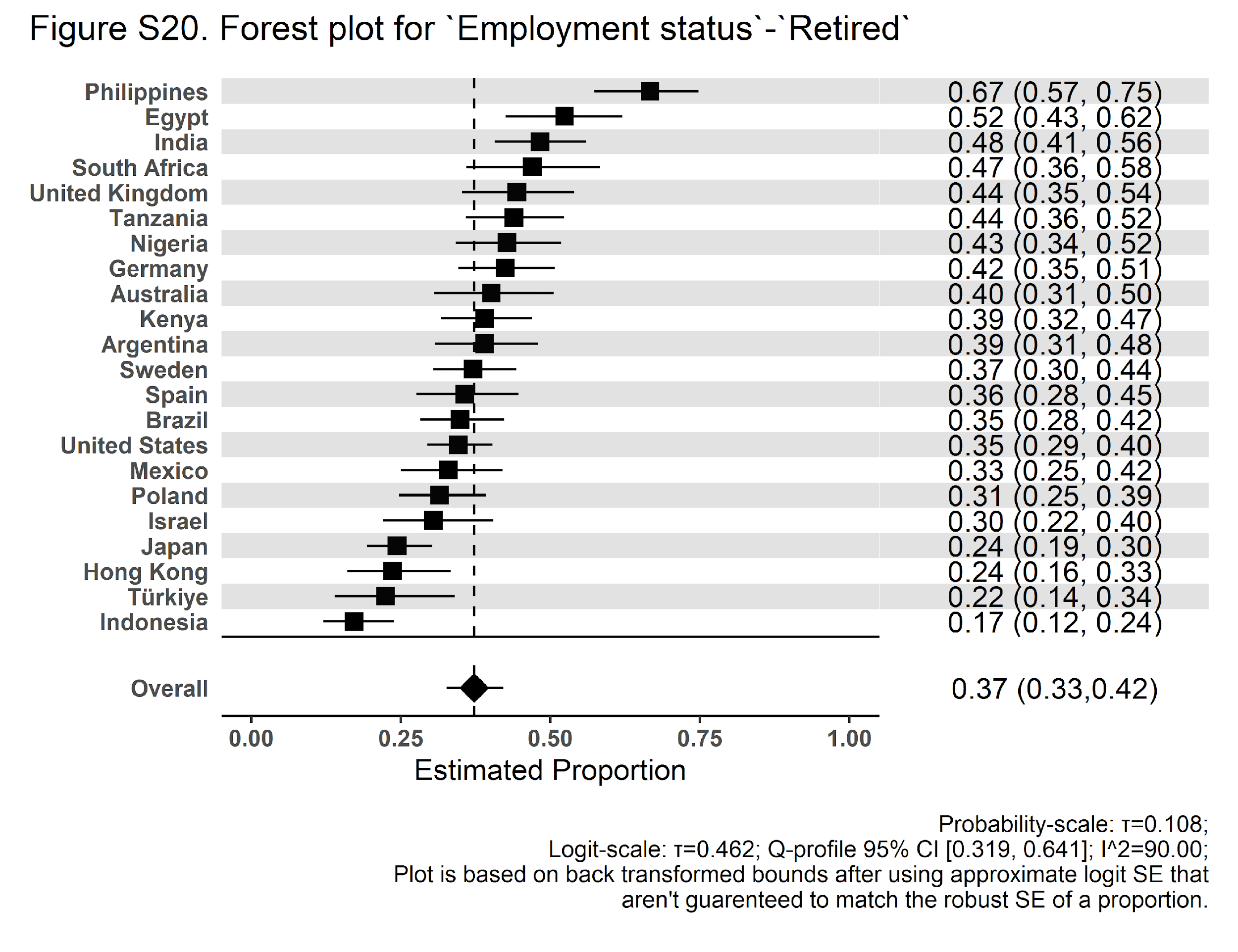

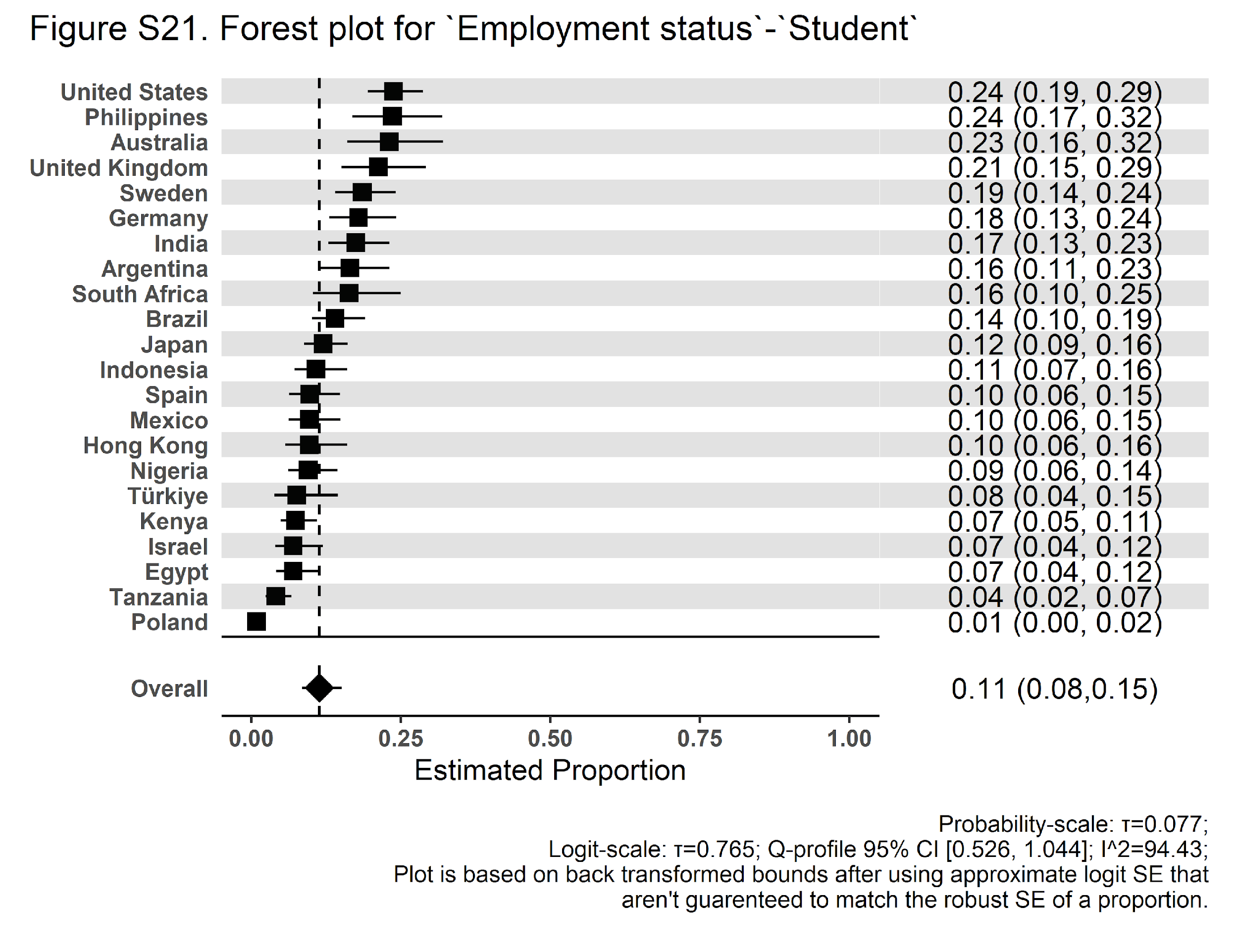

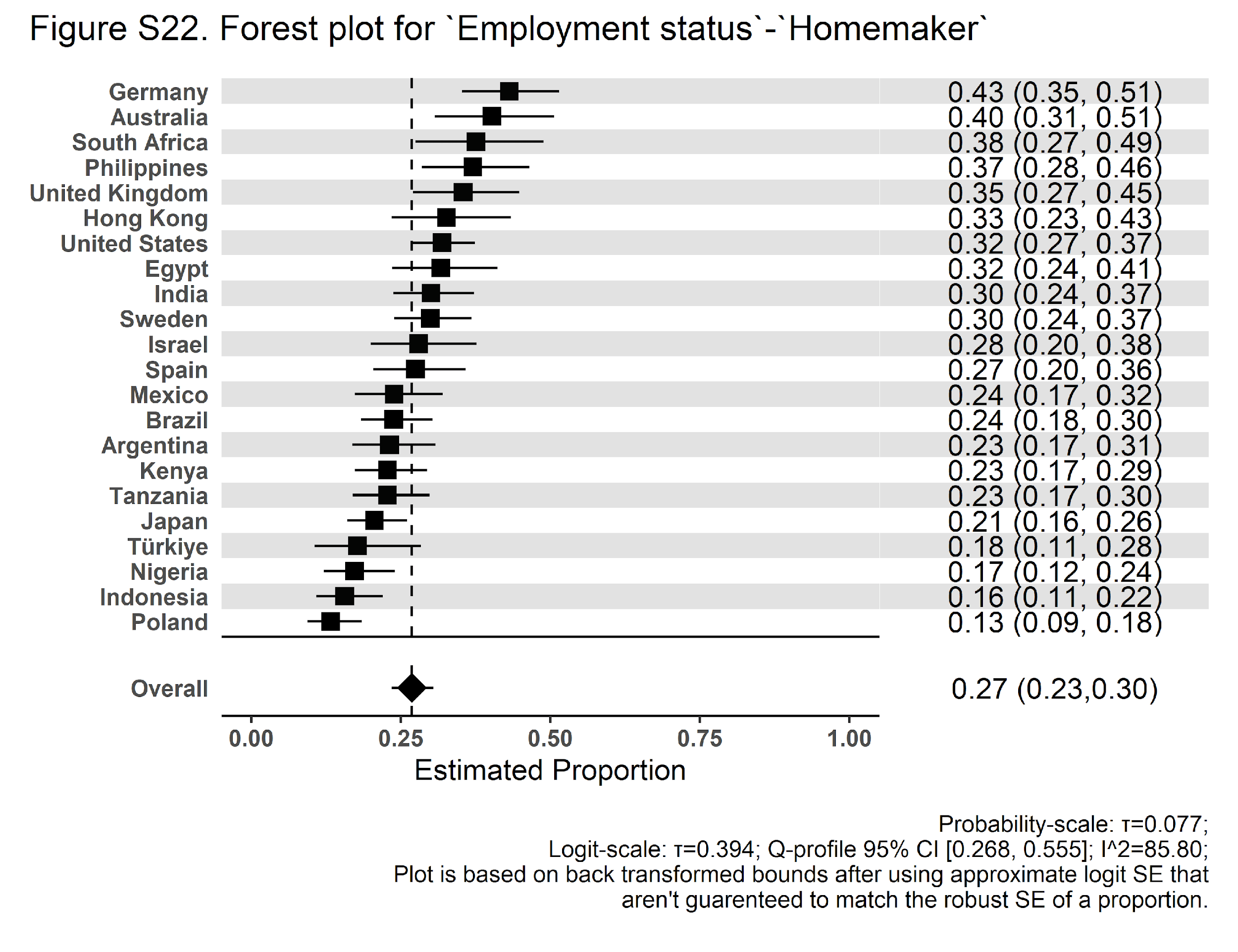

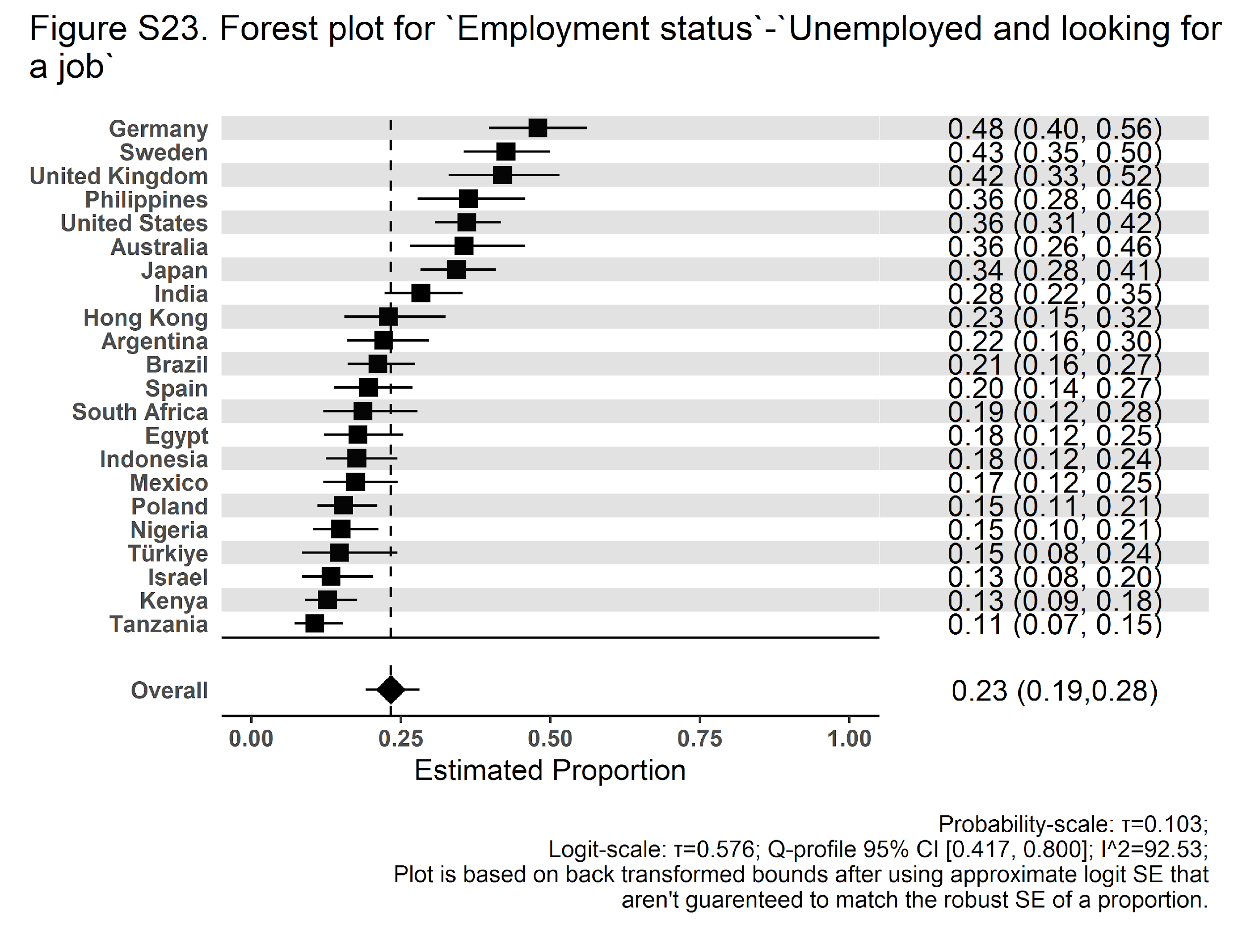

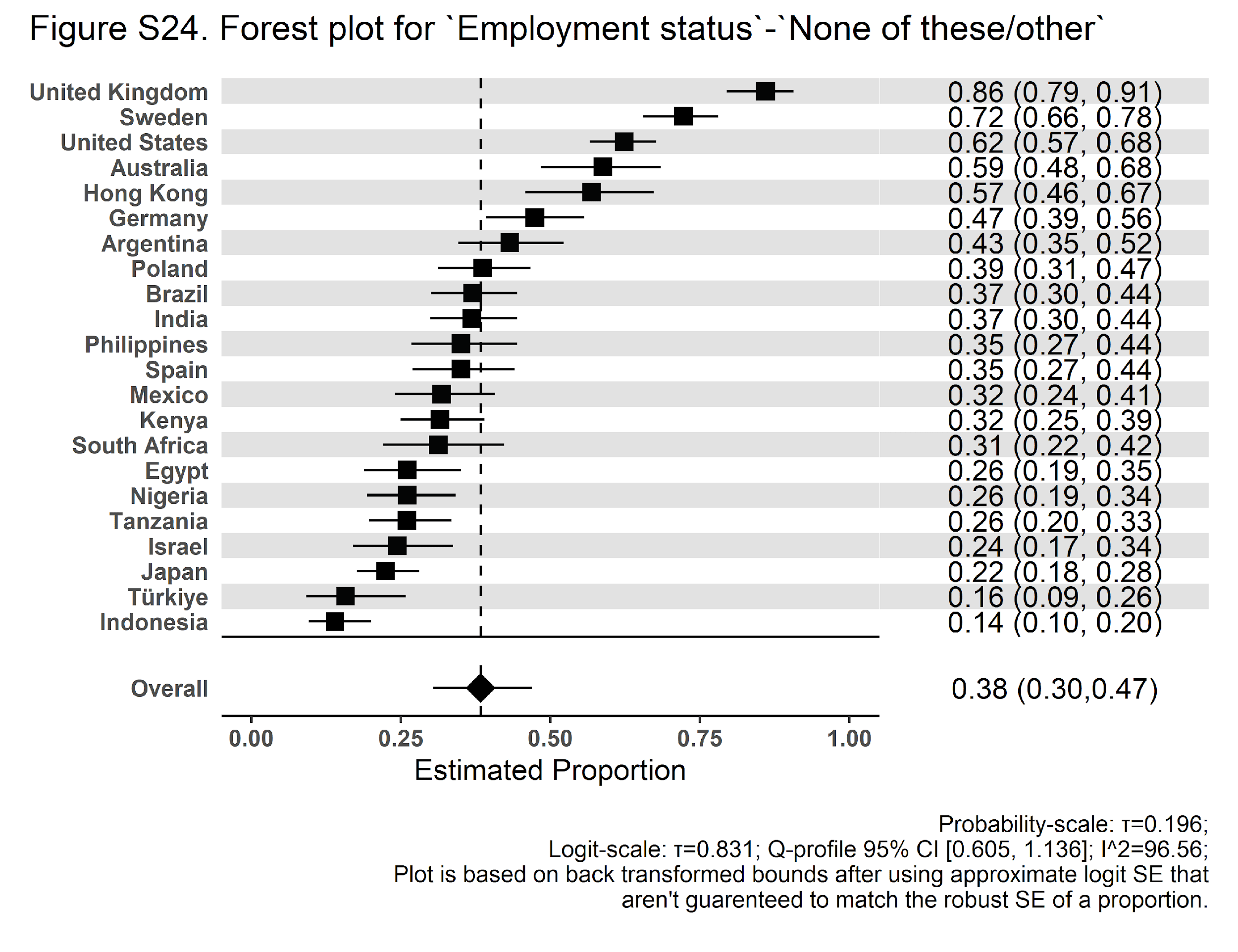

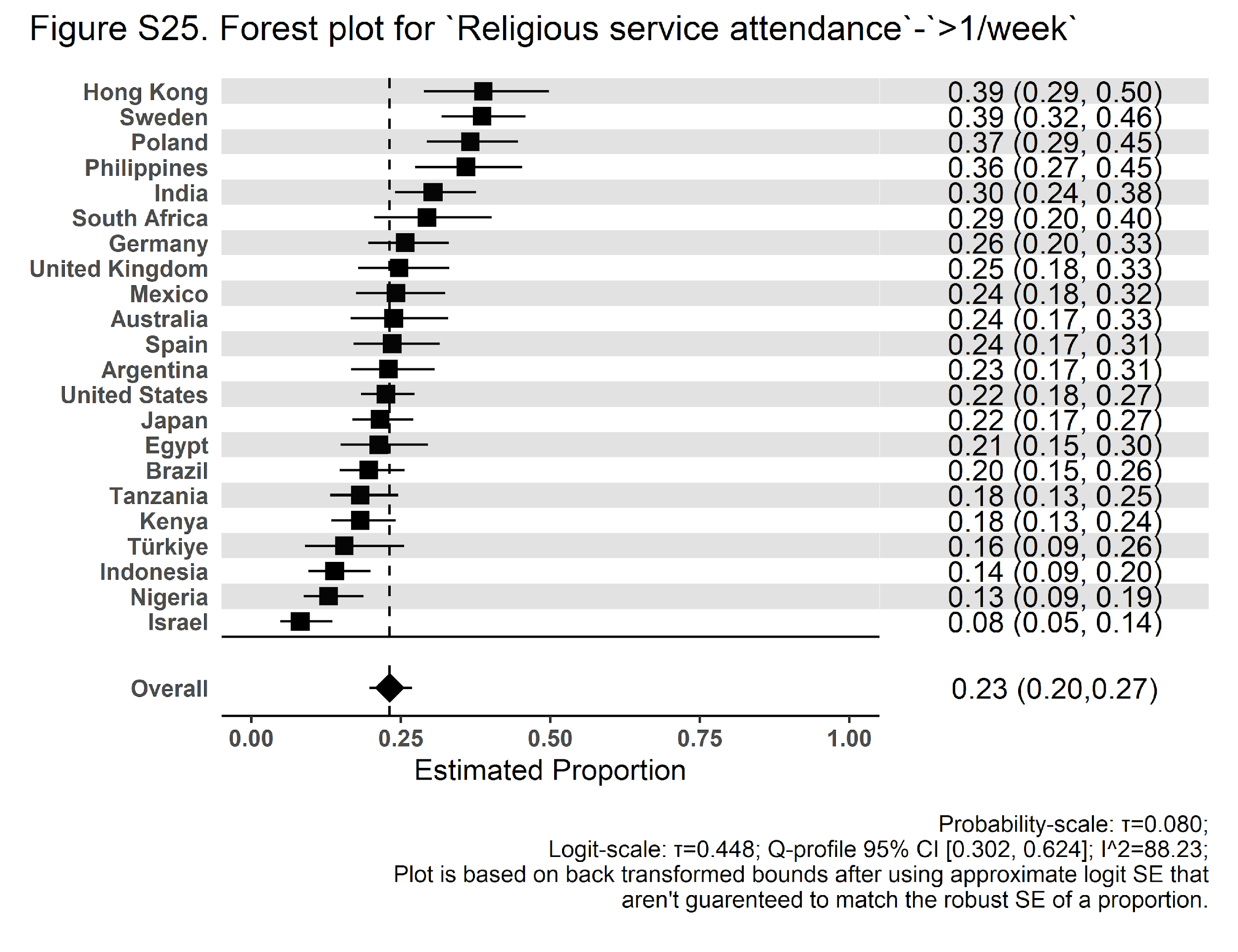

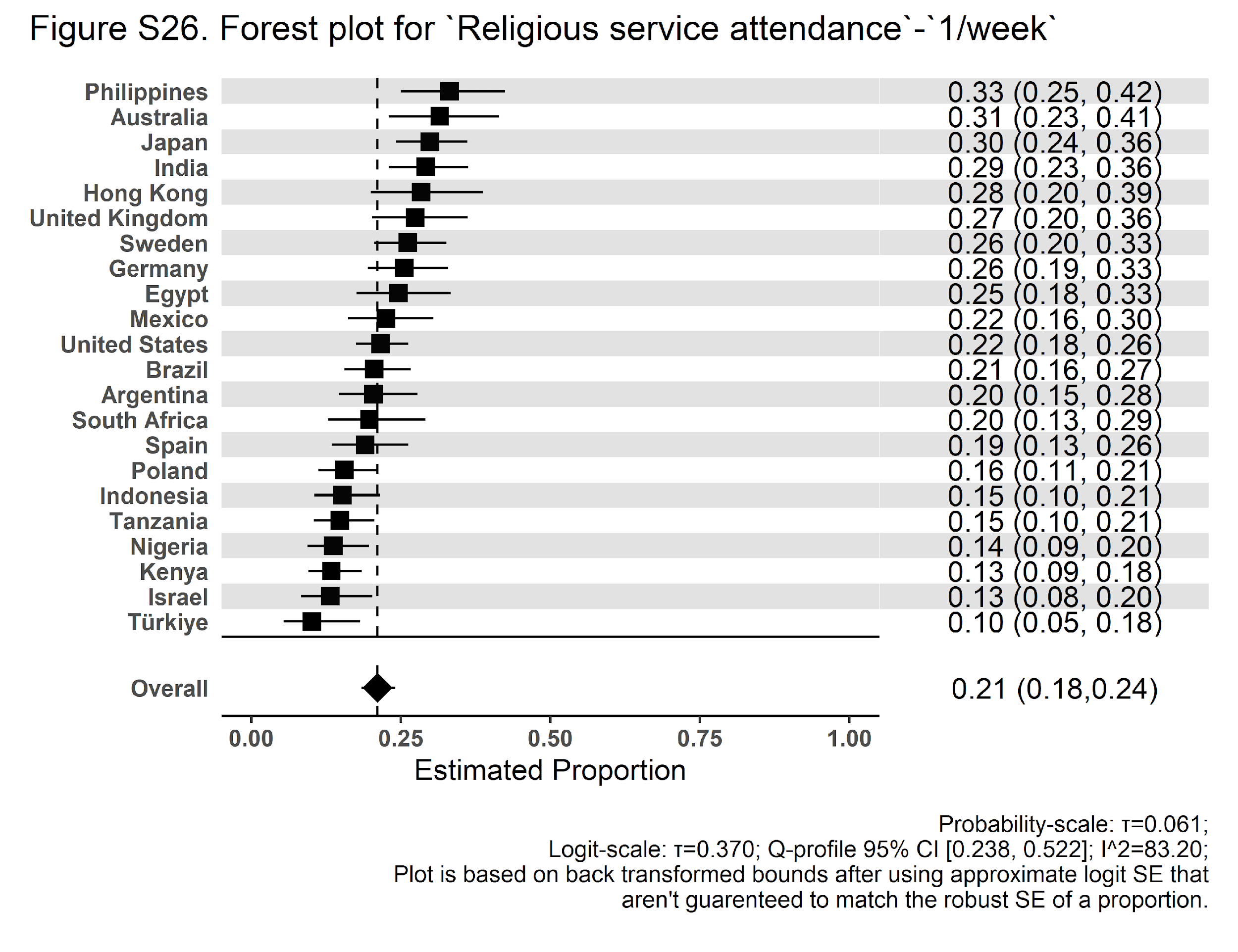

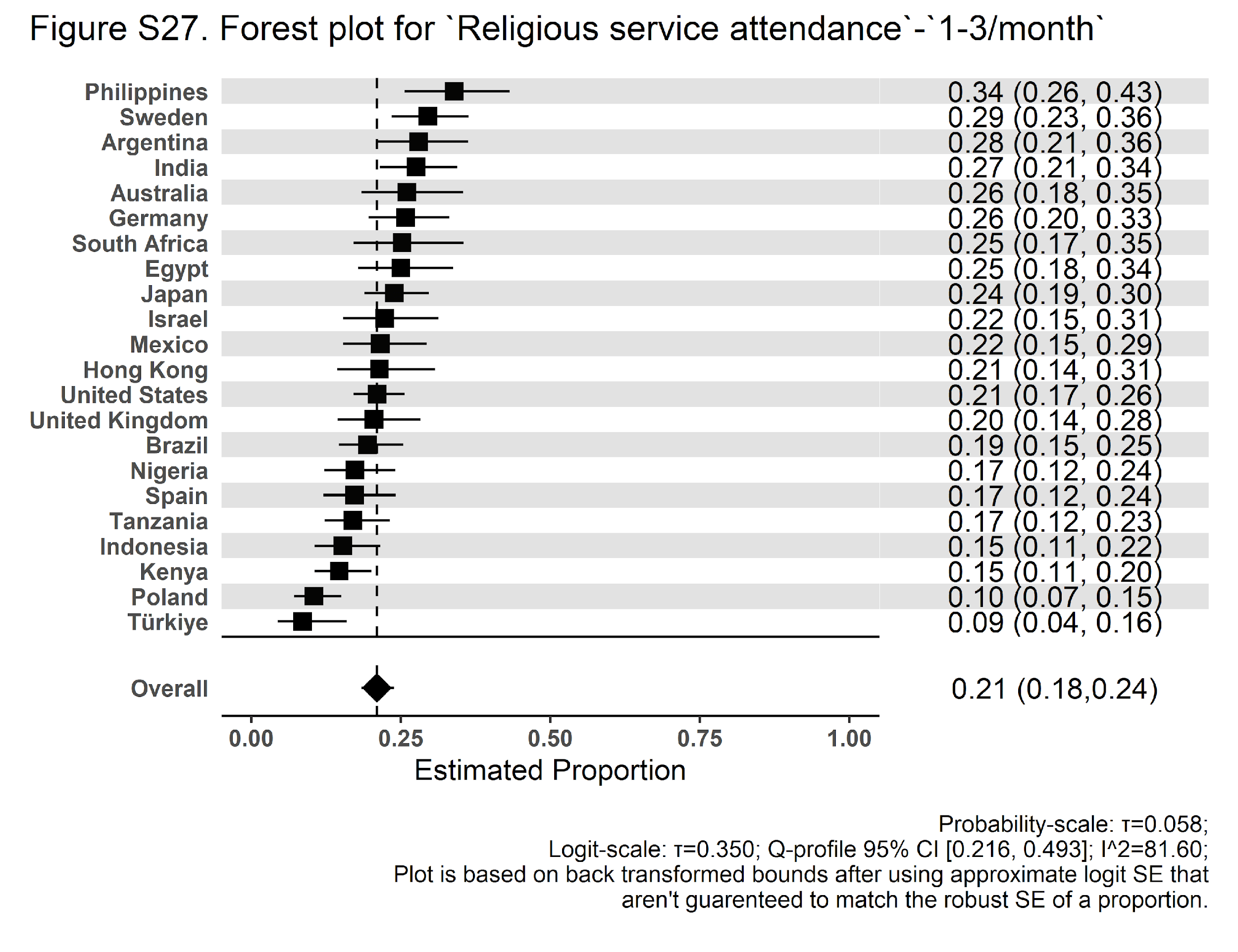

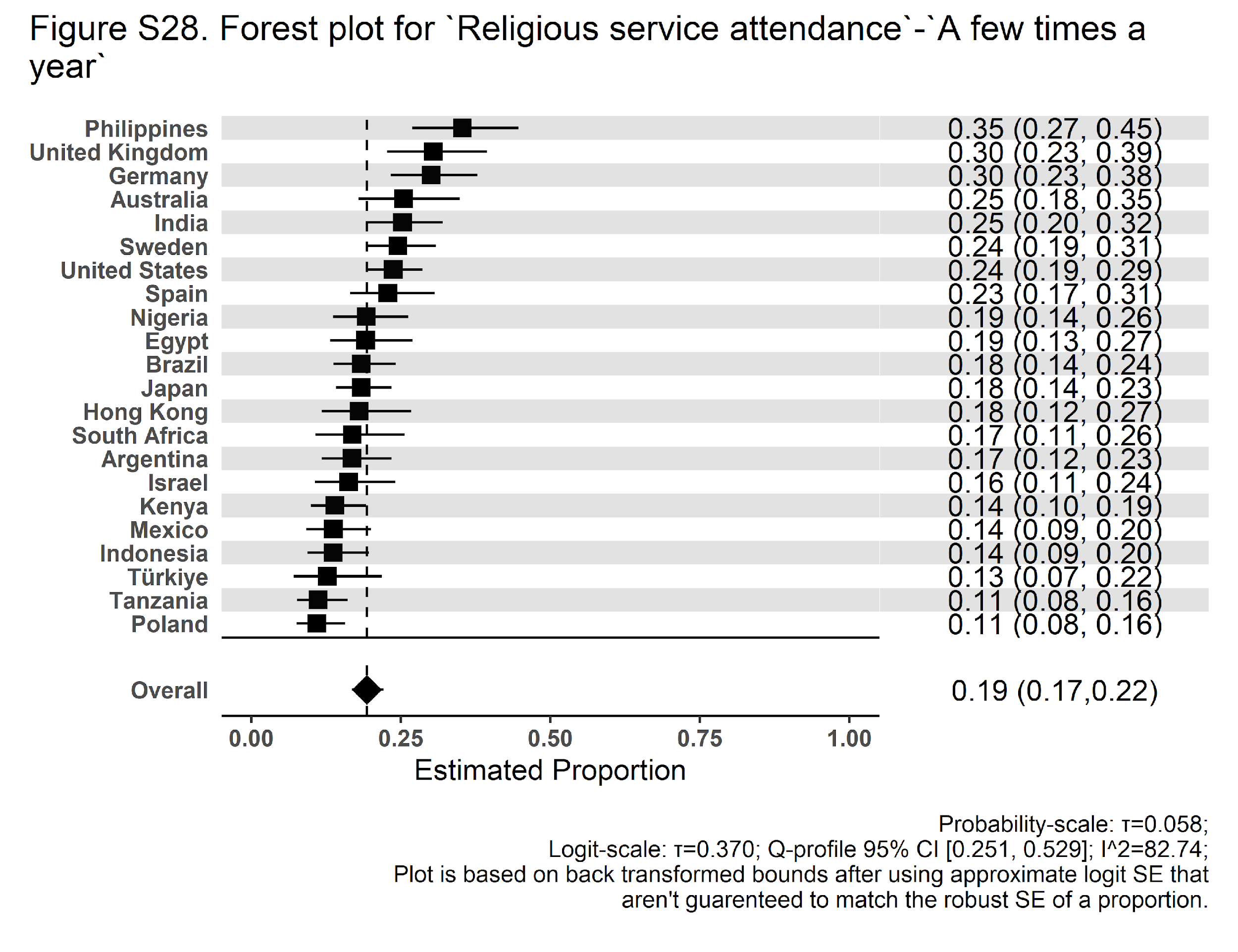

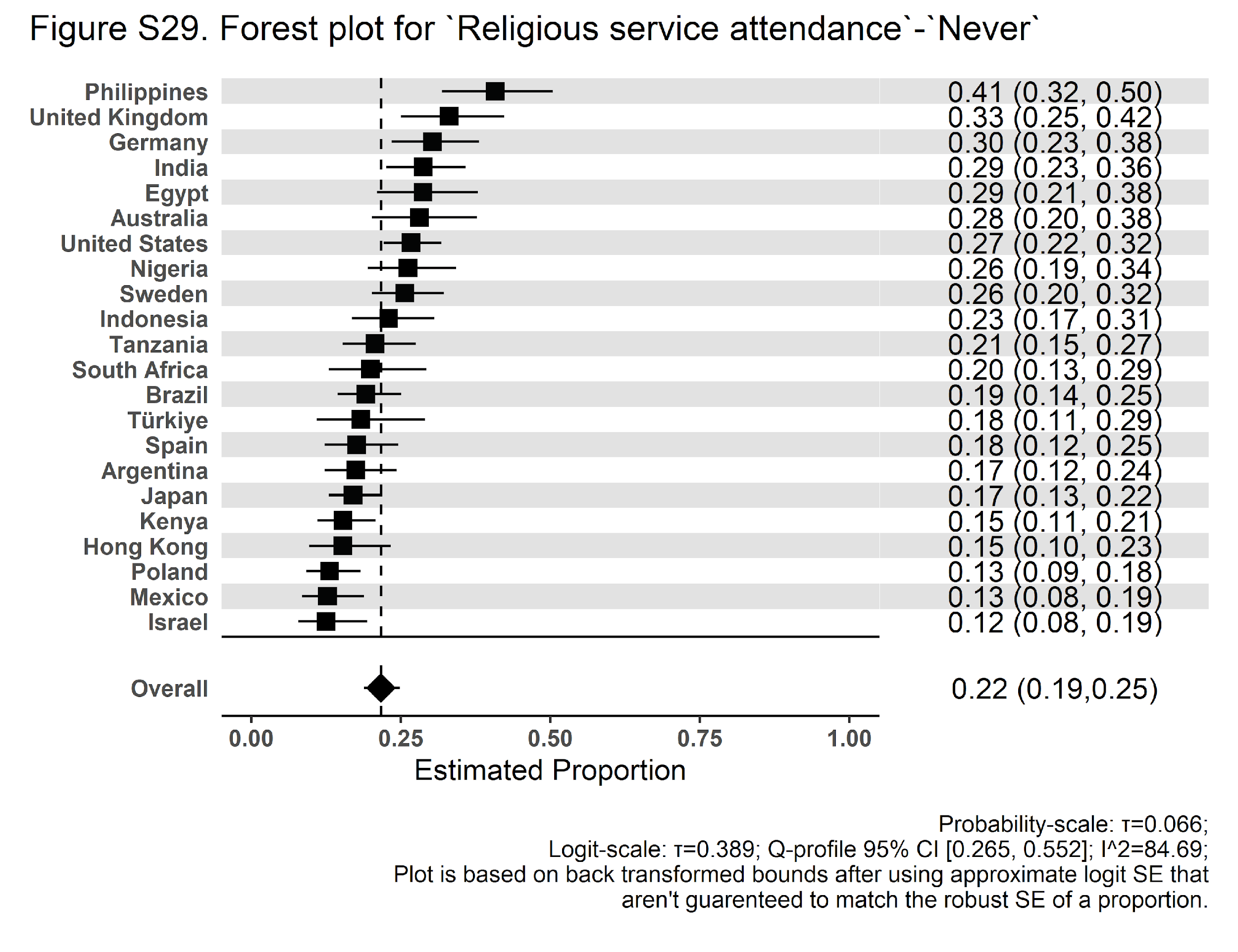

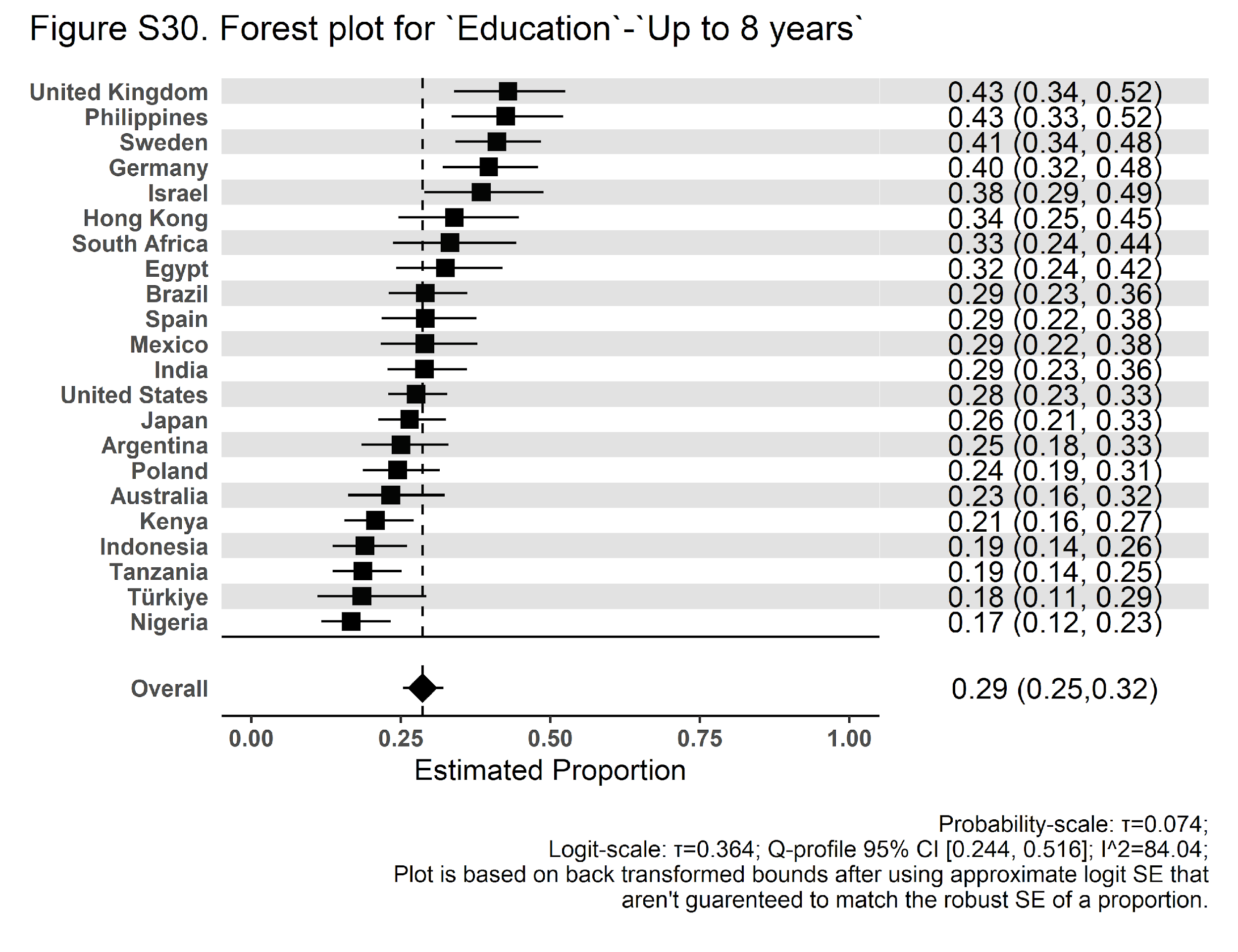

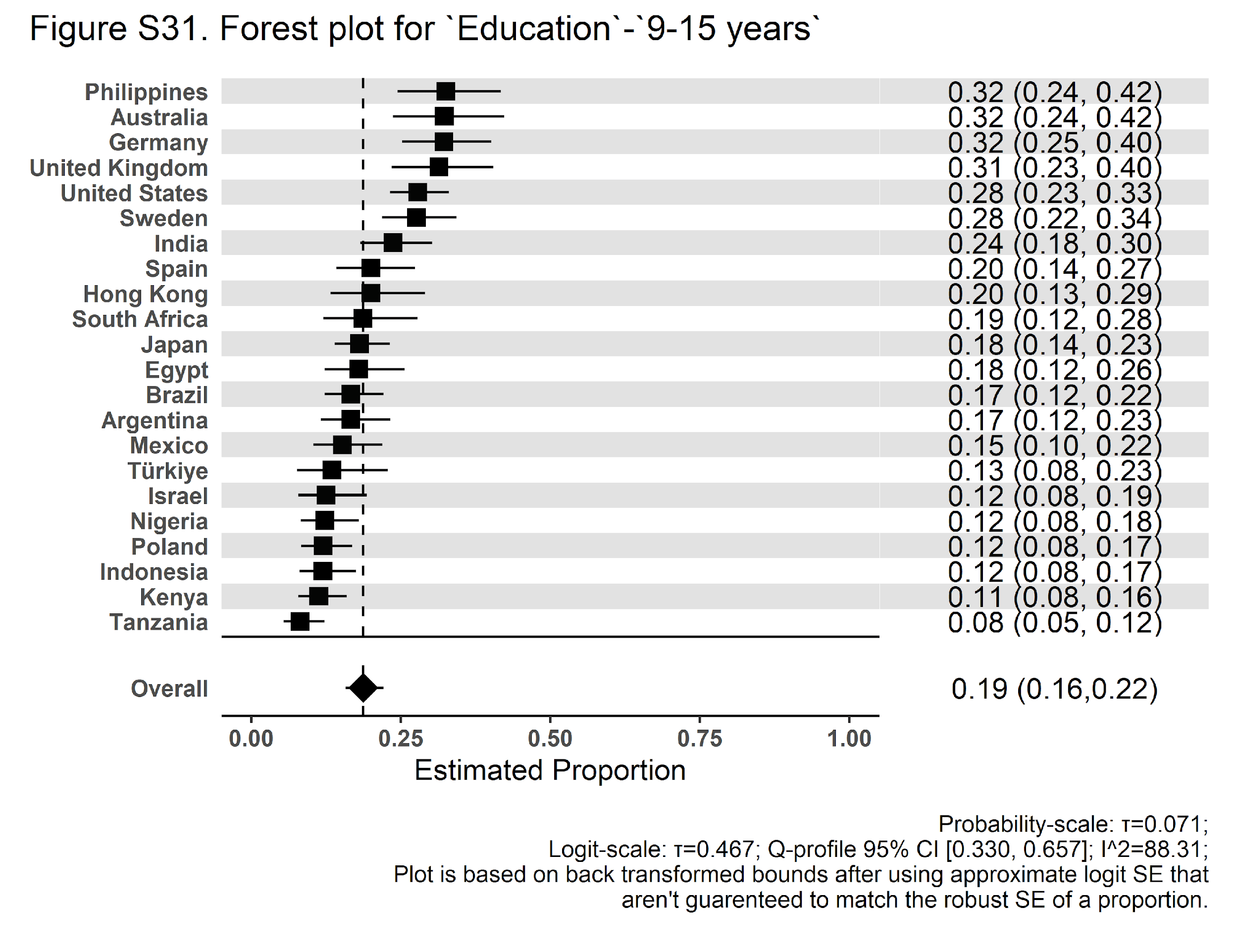

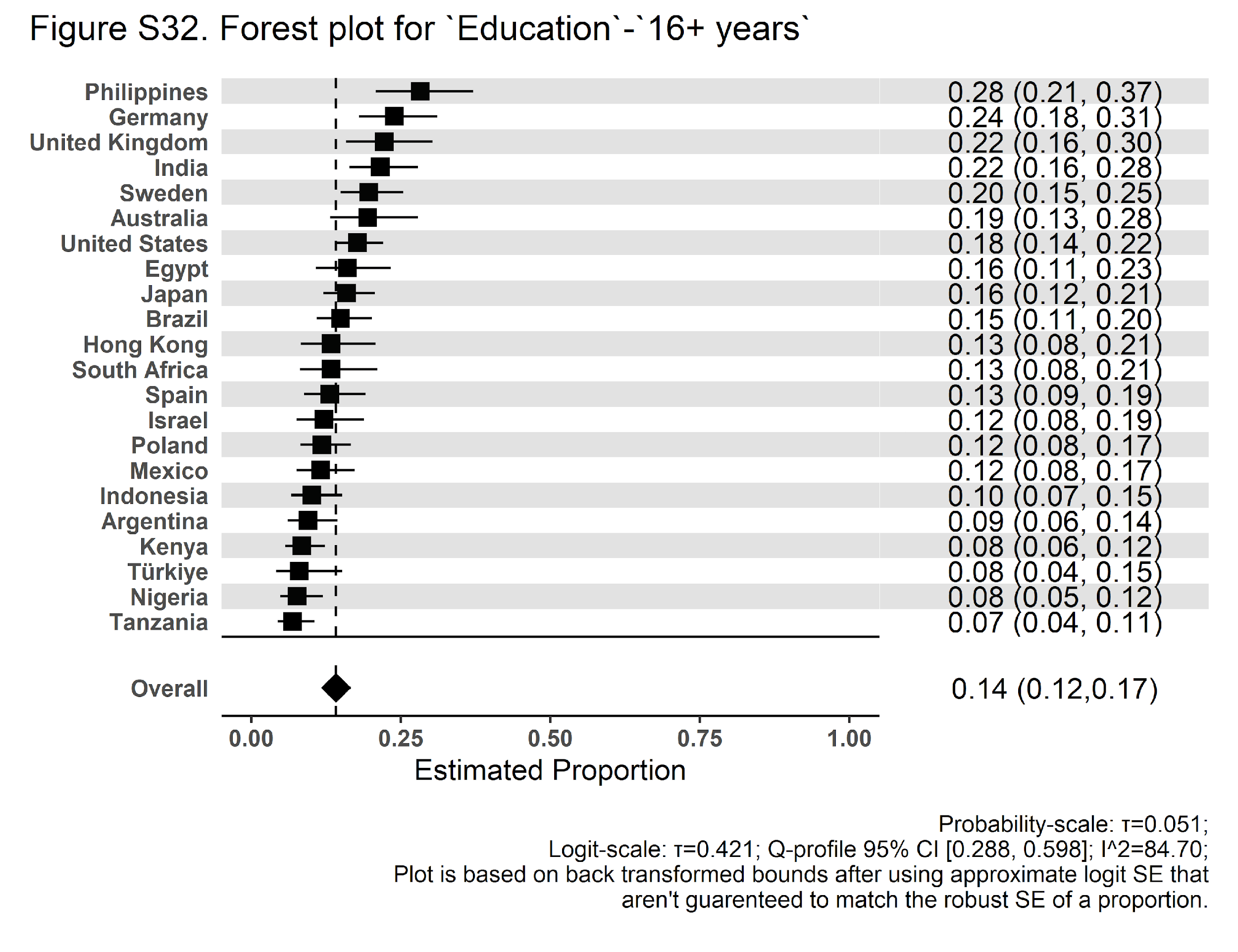

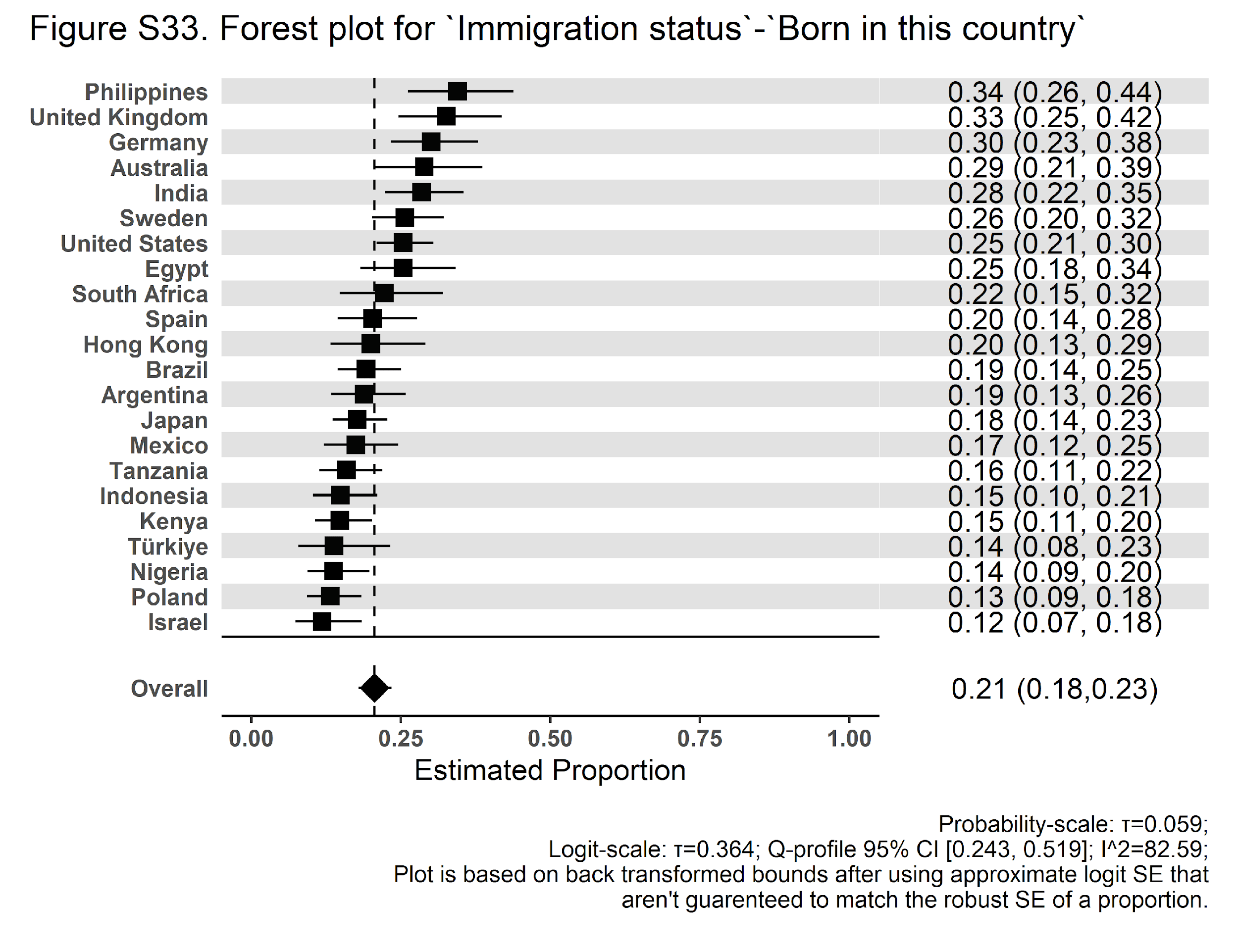

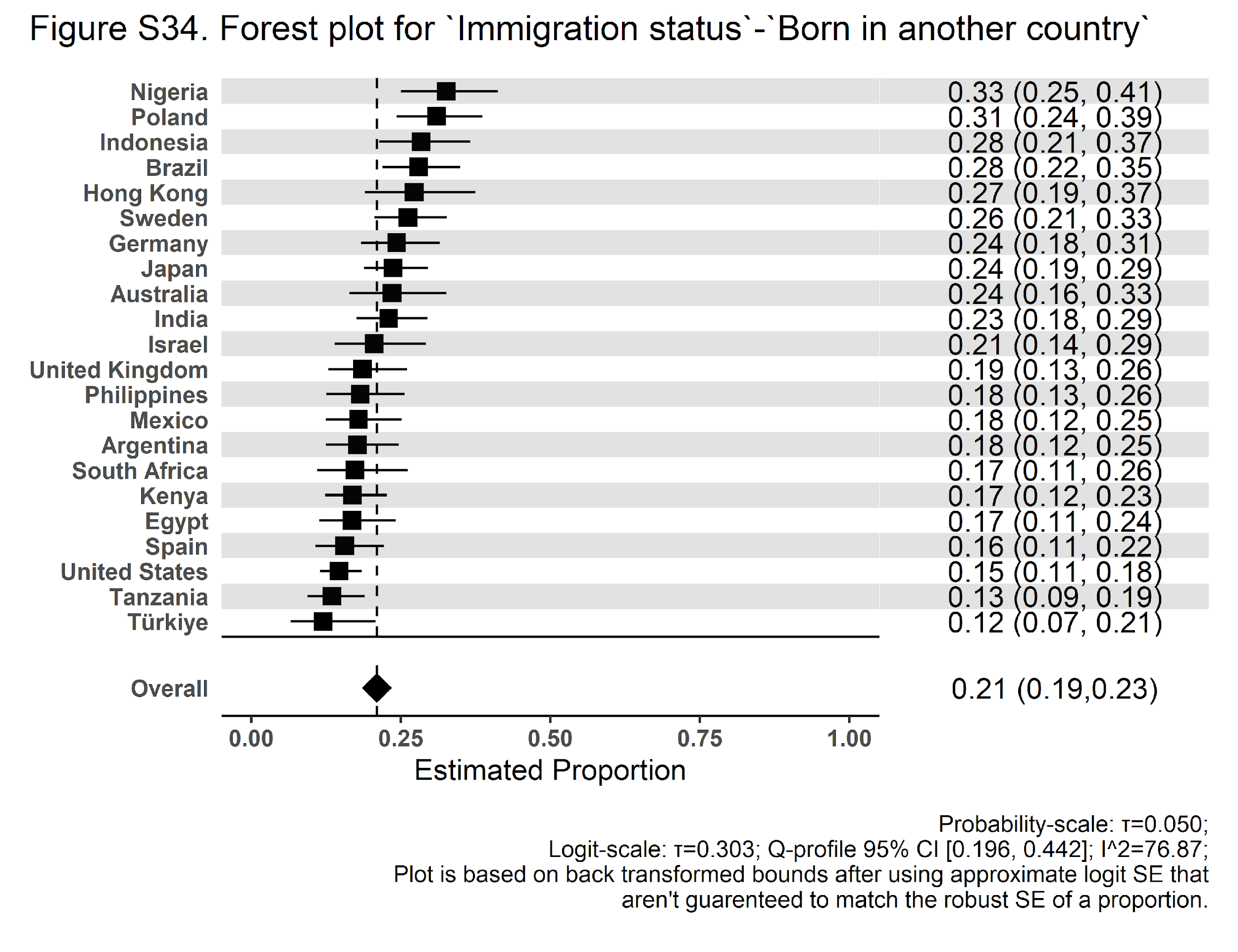

Supplement: Supplementary file 1 — Additional file 1. Table S1a: Nationally representative descriptive statistics for Argentina. Table S1b: Proportions by demographic category for Argentina. Table S2a: Nationally representative descriptive statistics for Australia. Table S2b: Proportions by demographic category for Australia. Table S3a: Nationally representative descriptive statistics for Brazil. Table S3b: Proportions by demographic category for Brazil. Table S4a: Nationally representative descriptive statistics for Egypt. Table S4b: Proportions by demographic category for Egypt. Table S5a: Nationally representative descriptive statistics for Germany. Table S5b: Proportions by demographic category for Germany. Table S6a: Nationally representative descriptive statistics for Hong Kong. Table S6b: Proportions by demographic category for Hong Kong. Table S7a: Nationally representative descriptive statistics for India. Table S7b: Proportions by demographic category for India. Table S8a: Nationally representative descriptive statistics for Indonesia. Table S8b: Proportions by demographic category for Indonesia. Table S9a: Nationally representative descriptive statistics for Israel. Table S9b: Proportions by demographic category for Israel. Table S10a: Nationally representative descriptive statistics for Japan. Table S10b: Proportions by demographic category for Japan. Table S11a: Nationally representative descriptive statistics for Kenya. Table S11b: Proportions by demographic category for Kenya. Table S12a: Nationally representative descriptive statistics for Mexico. Table S12b: Proportions by demographic category for Mexico. Table S13a: Nationally representative descriptive statistics for Nigeria. Table S13b: Proportions by demographic category for Nigeria. Table S14a: Nationally representative descriptive statistics for the Philippines. Table S14b: Proportions by demographic category for Philippines. Table S15a: Nationally representative descriptive statistics for Poland. Table S15b: Proportions by dem [file 44263_2025_190_MOESM1_ESM.docx]
